# Mesoniviruses are mosquito-specific viruses with extensive geographic distribution and host range

**DOI:** 10.1186/1743-422X-11-97

**Published:** 2014-05-20

**Authors:** Nikos Vasilakis, Hilda Guzman, Cadhla Firth, Naomi L Forrester, Steven G Widen, Thomas G Wood, Shannan L Rossi, Elodie Ghedin, Vsevolov Popov, Kim R Blasdell, Peter J Walker, Robert B Tesh

**Affiliations:** 1 Department of Pathology and Center for Biodefense and Emerging Infectious Diseases, University of Texas Medical Branch, Galveston, TX 77555-0609, USA; 2 Center for Tropical Diseases, University of Texas Medical Branch, Galveston, TX 77555-0609, USA; 3 Institute for Human Infection and Immunity, University of Texas Medical Branch, Galveston, TX 77555-0610, USA; 4 CSIRO Animal, Food and Health Sciences, Australian Animal Health Laboratory, Geelong, VIC 3220, Australia; 5 Department of Biochemistry and Molecular Biology, The University of Texas Medical Branch, Galveston, TX 77555-1079, USA; 6 Department of Computational Biology and Center for Vaccine Research, University of Pittsburgh, Pittsburgh, PA 15214, USA; 7 Current Address: NYU-Biology, Center for Genomics and Systems Biology, New York University, New York, NY 10032, USA

**Keywords:** Nidoviruses, Mesonivirus, Insect-specific, Phylogeny, Next generation sequencing

## Abstract

**Background:**

The family *Mesoniviridae* (order *Nidovirales*) comprises of a group of positive-sense, single-stranded RNA ([+]ssRNA) viruses isolated from mosquitoes.

**Findings:**

Thirteen novel insect-specific virus isolates were obtained from mosquitoes collected in Indonesia, Thailand and the USA. By electron microscopy, the virions appeared as spherical particles with a diameter of ~50 nm. Their 20,129 nt to 20,777 nt genomes consist of positive-sense, single-stranded RNA with a poly-A tail. Four isolates from Houston, Texas, and one isolate from Java, Indonesia, were identified as variants of the species *Alphamesonivirus-1* which also includes Nam Dinh virus (NDiV) from Vietnam and Cavally virus (CavV) from Côte d’Ivoire. The eight other isolates were identified as variants of three new mesoniviruses, based on genome organization and pairwise evolutionary distances: Karang Sari virus (KSaV) from Java, Bontag Baru virus (BBaV) from Java and Kalimantan, and Kamphaeng Phet virus (KPhV) from Thailand. In comparison with NDiV, the three new mesoniviruses each contained a long insertion (180 – 588 nt) of unknown function in the 5’ region of ORF1a, which accounted for much of the difference in genome size. The insertions contained various short imperfect repeats and may have arisen by recombination or sequence duplication.

**Conclusions:**

In summary, based on their genome organizations and phylogenetic relationships, thirteen new viruses were identified as members of the family *Mesoniviridae,* order *Nidovirales*. Species demarcation criteria employed previously for mesoniviruses would place five of these isolates in the same species as NDiV and CavV (*Alphamesonivirus-1*) and the other eight isolates would represent three new mesonivirus species (*Alphamesonivirus-5, Alphamesonivirus-6* and *Alphamesonivirus-7*). The observed spatiotemporal distribution over widespread geographic regions and broad species host range in mosquitoes suggests that mesoniviruses may be common in mosquito populations worldwide.

## Background

The recently established virus family *Mesoniviridae* (order *Nidovirales*) comprises a group of positive-sense, single-stranded RNA ([+]ssRNA) insect viruses [1]. To date, the six described mesoniviruses, Cavally (CavV), DakNong (DKNG), Hana (HanaV), Meno (MenoV), Nam Dinh (NDiV) and Nse (NseV) have all been isolated from naturally infected mosquitoes collected in just two countries: Côte d’Ivoire (West Africa) and Vietnam (Southeast Asia) [2-5]. Although these mosquito-associated viruses do not appear to infect vertebrates or to cause illness in humans or livestock, they are nonetheless of interest because of the structural and genetic similarities to other members of the order *Nidovirales*, namely viruses in the families *Coronaviridae, Arteriviridae,* and *Roniviridae*. Furthermore, the basal phylogenetic position of the mesoniviruses in relation to the *Coronaviridae* has led some authors to suggest that the coronaviruses, and possibly other viruses in the order *Nidovirales*, may have evolved in arthropods [3-5]. Indeed, members of the *Roniviridae* naturally infect marine shrimp and can cause severe pathology in these economically important arthropods [6].

In this communication, we report the isolation and characterization of 13 additional mesoniviruses from mosquitoes collected in Thailand, Indonesia and the United States of America (USA). These 13 viruses appear to represent four distinct species in the family *Mesoniviridae*, three of which are novel. Based on their wide geographic distribution, the limited sampling that has been done to date for these mosquito-specific viruses, and their broad species host range in mosquitoes, it seems likely that mesoniviruses are common in mosquito populations worldwide. The potential biological significance and effect of mesoniviruses on mosquito vector competence is also discussed.

## Results

### Virus morphology

**Figure 1:**
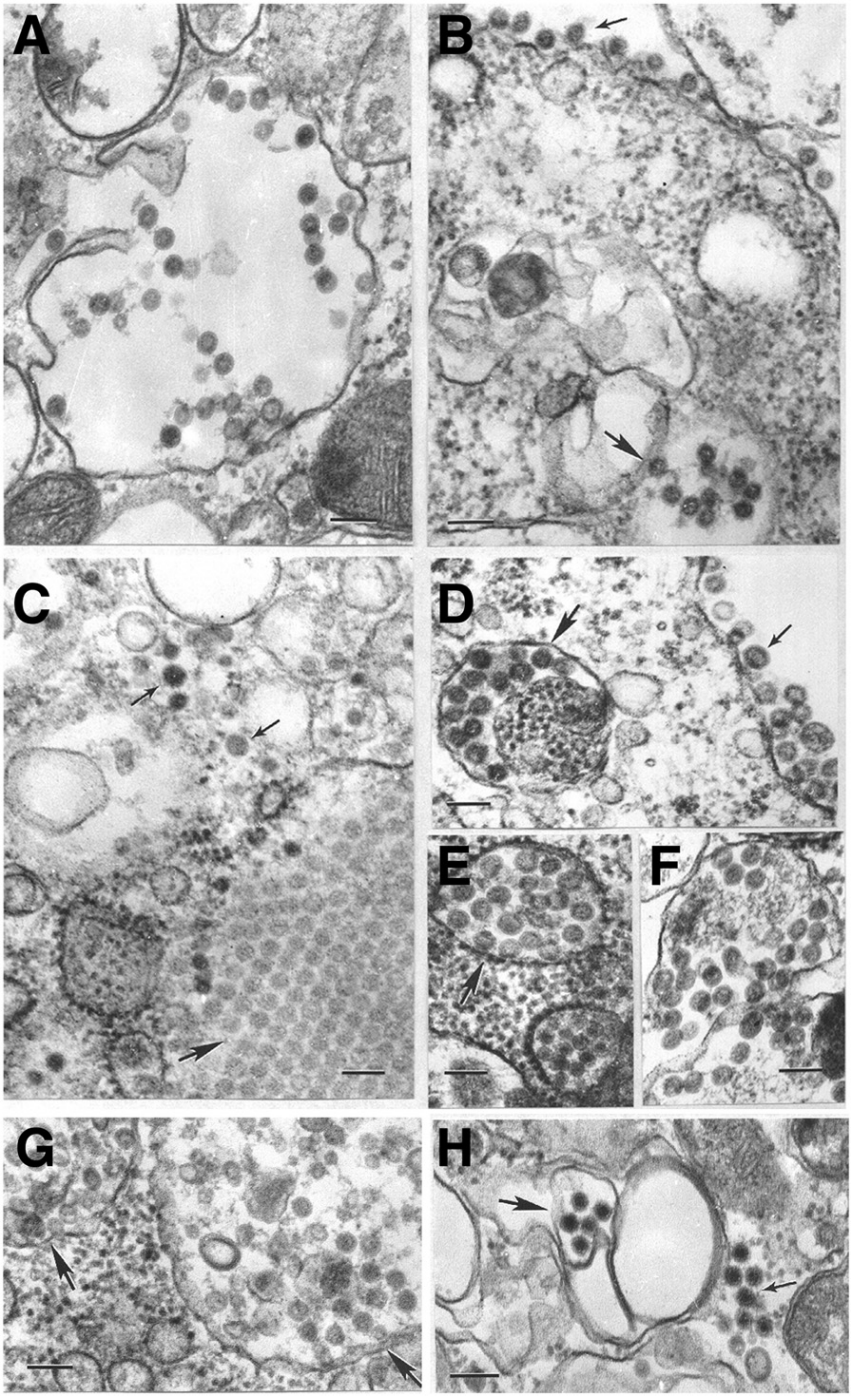
**Ultrastructure of mesoniviruses in C6/36 cells.** Bars = 100 nm. **(A)** virus particles of Nam Dinh strain V3872 inside a cytoplasmic vacuole; **(B)** virions of Bontag Baru JKT-9876 strain at the cell surface (thin arrow) and inside a cytoplasmic vacuole (thick arrow); **(C)** virions of Bontag Baru strain JKT-9876 free in the cytosol (thin arrows). Thick arrow indicates a portion of a paracrystalline agglomerate of empty virus particles in the host cell cytosol; **(D)** virions of Bontag Baru strain JKT-9853 at the surface of the cell (thin arrow) and inside a cytoplasmic vacuole (thick arrow); **(E)** virus particles of Karang Sari (JKT-10701) inside an expanded cistern of granular endoplasmic reticulum (thick arrow); **(F)** virus particles of Bontag Baru JKT-7774 inside a cytoplasmic vacuole; (**G** and **H**) virus particles of Kamphaeng Phet strain KP84-0156 inside cytoplasmic vacuoles (thick arrows) and free in cytosol (thin arrow in **H**).

All isolates had similar ultrastructure. Mature virions ~50 nm in diameter were located at the surface of infected C6/36 cells, either as individual particles or in groups (Figure 1B,D), similar to what has been reported recently [7]. They displayed a dense nucleocapsid core ~40 nm in diameter and surrounding envelope (Figure 1A,B). Mature virions of the same size could also be found inside intracytoplasmic vacuoles (Figure 1A,B,D,E,F,H**)**. Some infected cells had paracrystalline arrays consisting of empty and full virus particles but with less electron-density than mature virions (Figure 1C). At the periphery of these arrays, mature virions could be observed either free in the cytosol or inside vacuoles (Figure 1C). Moreover a recent report [8], suggests that the mesonivirus virion diameter of ~40-50 nm observed by us and others [7] may be significantly lower than its actual size, a discrepancy attributed to the method of preparation for alternative imaging technologies. Employing cryo-electron tomography, Warrilow et al. [8] demonstrated on the surface of some virions the presence of spike-like projections displaying a globular head attached to the virion surface through a low density stalk, similar to what has been observed in other members of the order *Nidovirales*[9].

### Genome sequence and organization

**Figure 2:**
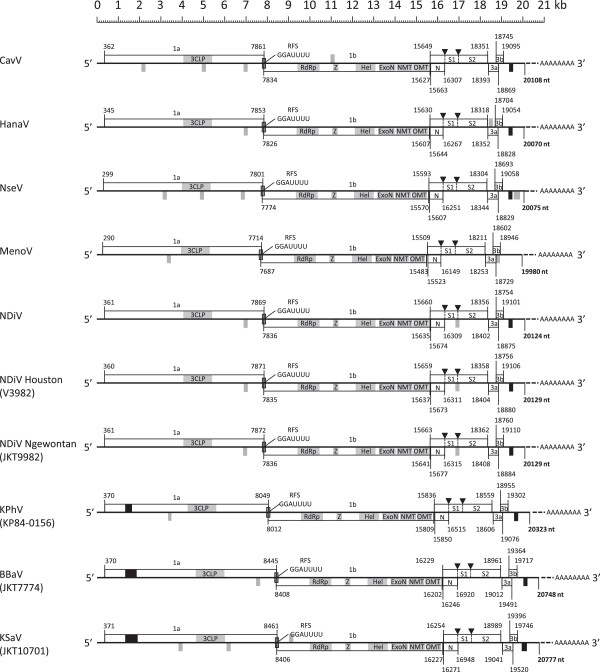
**Genome organisation of the mesoniviruses.** In ORF1a and ORF1b the putative ribosomal frame-shift (RFS) element and the locations of conserved functional domains, including the 3C-like serine protease (3CLP), RNA-dependent RNA polymerase (RdRp), zinc-binding (Z), helicase (Hel), exoribonuclease (ExoN), N7-methyltransferase (NMT) and 2’-O-methyltransferase (OMT) domains, are shown. Sequence insertions in ORF1a are shown as black blocks. In ORF2a encoding the S glycoprotein the sites of proteolytic cleavage to generate S1, S2 and the uncharacterised N-terminal fragment are shown as black inverted triangles. Putative ORF4 that is relatively conserved in the 3’terminal region of the genome is shown as black rectangles and other small ORFs >180 nt that occur variously in the 3’-terminal domains and elsewhere in the genome are shown as grey rectangles.

The complete nucleotide sequences of all 13 isolates were determined by high-throughput Illumina sequencing with end-finishing by 5’- and 3’-RACE. Excluding the 3’-poly [A] tail, the (+) ssRNA genomes ranged in size from 20,127 nt (V3872) to 20,777 nt (JKT-10701). The organization of each genome was similar to that described previously for the mesoniviruses (NDiV, CavV, HanaV, NseV and MenoV), featuring a long 5’-untranslated region (5’-UTR) of 359 to 370 nt, six major long open reading frames (ORFs), and a long terminal region of 1780 to 1804 nt preceding the poly[A] tail (Figure 2). An alignment that also included the five previously described mesoniviruses revealed block insertions in three groups of isolates: i) KP84-0156, KP84-0192 and KP84-0344 had a 180 nt insertion, ii) JKT-9853, JKT-9876, JKT-9891 and JKT-7774 had a 573 nt insertion, and iii) JKT-10701 had a 588 nt insertion. These insertions accounted for most of the observed difference in genome size in the mesoniviruses (Figure 2). Excluding this region, which commenced ~1300 nt from the 5’-terminus, the mesoniviruses shared global nucleotide sequence identity of ~43.7%. Maximum pairwise divergence was between MenoV and all other viruses (65.5% - 67.6% identity).

### Phylogenetic analysis and species determination

**Figure 3:**
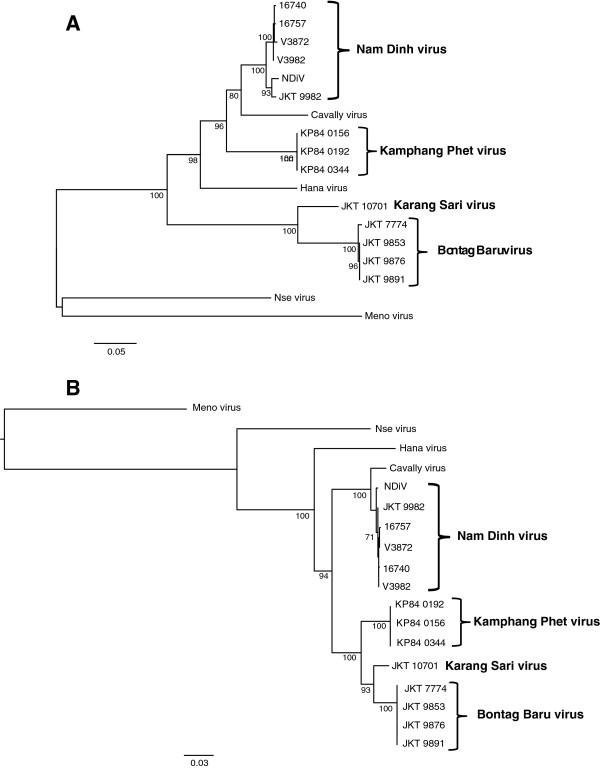
**Phylogenetic analysis of the mesonivirus proteins. (A)**. Maximum-likelihood phylogeny of the full-length proteins encoded in ORF2a (unprocessed S proteins). **(B)**. Maximum-likelihood analysis of conserved protein domains of ORF1ab (3CL^pro^, RdRp, HEL1). Scale bars indicate amino acid substitutions/site.

To determine the phylogenetic relationships of the newly identified insect viruses, maximum likelihood (ML) phylogenetic trees were constructed based on the amino acid alignments of ORF2a (unprocessed S protein) and a concatenated region of the highly conserved domains within ORF1ab (3CL^pro^, RdRp and ZnHel1). The phylogenies exhibited highly similar topologies and strongly indicated that the viruses identified in this study cluster within the previously identified insect-specific nidoviruses in the genus *Mesonivirus* (NDiV, CavV, HanaV, NseV and MenoV) (Figure 3).

A multiple sequence alignment of the 13 new genomes indicated high identity between multiple isolates, which was also confirmed by our phylogenetic analysis. Isolates KP84-0156, KP84-0192 and KP84-0344 differed only by ten nucleotide substitutions and a single 6-nt indel and formed a well-supported monophyletic clade in both phylogenies (Figure 3). These should be considered isolates of the same virus (designated Kamphang Phet virus - KPhV). Likewise, JKT-9853, JKT-9876 and JKT-9891 differed by only single nucleotide substitutions and JKT-7774 differed by 68 nucleotide substitutions and two indels of 3 and 6 nucleotides, and these four viruses formed a strongly supported monophyletic clade in the ML trees, suggesting that they can be considered isolates of the same virus (designated Botang Baru virus - BBaV). Isolates 16740, 16757, V3872 and V3982 were also closely related, differing by only 18–57 nucleotide substitutions (~0.1-0.3%) across the entire genome. The clade formed by these isolates in each ML tree clustered within a closely related monophyletic group that also included NDiV and isolate JKT-9982 (Figure 3). These can all be considered strains of NDiV. Finally, isolate JKT-10701 was clearly distinct from all other mesoniviruses in our phylogenetic analysis, and was considered a unique virus, Karang Sari (KSaV).

It has recently been suggested that pairwise evolutionary distances (PED) can be used as a species demarcation criterion in the genus *Mesonivirus*[6]. We calculated the PED between the 13 new isolates described in this study and those of five previously described mesoniviruses (CavV, HanaV, NDiV, NseV and MenoV) using the conserved protein domains of ORF1ab (3CL^pro^, RdRp and ZnHel1). Based on this analysis, NDiV (including the Ngewontan and Houston strains) and CavV can be considered a single species, which has previously been designated at *Alphamesonivirus-1*. The PED between each of these strains was substantially less than the suggested cutoff of 0.032 for species demarcation [6]. By this method, each of the other previously described mesoniviruses - HanaV MenoV, and NseV – would also be considered unique species, as the PED between these viruses and all other mesoniviruses were considerably greater than 0.032. These viruses could be assigned to the species *Alphamesonivirus-2, Alphamesonivirus-3* and *Alphamesonivirus-4*, respectively. Similarly, novel viruses BBaV, KSaV and KPhV would each also be assigned to separate mesonovirus species: *Alphamesonivirus-5, Alphamesonivirus-6* and *Alphamesonivirus-7*, respectively. Strikingly, the vast majority of the genetic diversity that was measured within the genus *Mesonivirus* was present at the inter-species level (Additional file 1: Figure S1). This pattern strongly supports the assignment of the current diversity of mesoniviruses into seven distinct species.

### Open reading frames

In mesoniviruses, ORF1a features a 3C-like protease (3CLpro) domain and flanking transmembrane domains, which are highly conserved amongst nidoviruses; these domains were identified in each of the new viruses. However, the block nucleotide sequence insertions resulted in a highly variable region near the N-terminus of ORF1a with insertions of various lengths ranging from 13 aa (MenV) to 196 aa (KSaV) (Additional file 2: Figure S2). The inserted sequences (which apparently are unique to the mesoniviruses) featured variations on a common sequence (SKRKGK) at the terminal points. There were also various short stretches of this sequence and other imperfectly repeated sequences within each insertion region suggesting possible origins through recombination or sequence duplication events.

As has been reported previously for other mesoniviruses, ORF1a is overlapped by ORF1b which contains highly conserved regions associated with the replicase complex including RNA-dependent RNA polymerase (RdRp), multinucleate zinc-binding module and associated helicase (ZnHel1), exoribonuclease (ExoN), N^7^-methyltransferase (NMT) and 2’-*O*-methyltransferase (OMT) domains. The ORF1a/ORF1b overlap includes a putative ‘slippery’ sequence (GGAUUUU), allowing ORF1b expression as a pp1ab polyprotein through a -1 ribosomal frame shift.

ORF2a encodes the S glycoprotein. In CavV, the S protein (p77) has been shown to be generated from the full-length ORF2a polyprotein by internal signalase cleavage at the site _220_NAHC|STRID and is further processed to generate cleavage products S1 (p23) and S2 (p57), each of which is a structural component of virions [5]. A MUSCLE alignment of all available mesonivirus ORF2a polyprotein sequences (Additional file 3: Figure S3) indicated that the signalase cleavage site is relatively variable conforming only to the sequence motif [C/S]|[L/A/S]TRIDL and that the S1-S2 cleavage site (R|WDSSYV) is highly conserved. The S protein, a class I transmembrane glycoprotein with a C-terminal transmembrane domain, features a set of 12 conserved cysteine residues which are likely to form 6 disulphide bridges, and 7 to 11 potential N-glycosylation sites, only 4 of which are conserved across all mesoniviruses. Surprisingly, an N-terminal signal peptide is strongly predicted for the MenoV S protein (between amino acids A^20^ and S^21^) but not for those of any of the other mesoniviruses (http://www.cbs.dtu.dk/services/SignalP). There is a relatively high level of amino acid sequence conservation in the S1 and S2 proteins (47.2% and 51.7% global identify, respectively). However, the N-terminal product of signalase cleavage of the ORF2a polyprotein displays very low sequence conservation amongst the mesoniviruses (7.4% global identity). This product, which is predicted to be positioned on the inside of the ER membrane (TMHMM server; http://www.cbs.dtu.dk/services/TMHMM-2.0) has not yet been identified in virions or infected cells.

In each of the mesoniviruses, the precise region of the genome encoding the highly variable N-terminal signalase cleavage product of the ORF2a polyprotein also contains an alternative open reading frame (ORF2b) which has been shown in CavV to encode the putative nucleoprotein p25 [5]. Our analysis indicated that the predicted molecular weights of the unmodified mesonivirus N proteins range from 23.69 kDa (NgeV) to 25.38 kDa (KSaV) and all are highly basic (pI >10). They display a moderate level of overall sequence conservation (34.1% global identity) due primarily to two highly conserved domains corresponding in KSaV to G^95^ to A^176^ (64.3% global sequence identity) and L^195^ to F^210^ (75.0% global sequence identity).

**Figure 4:**
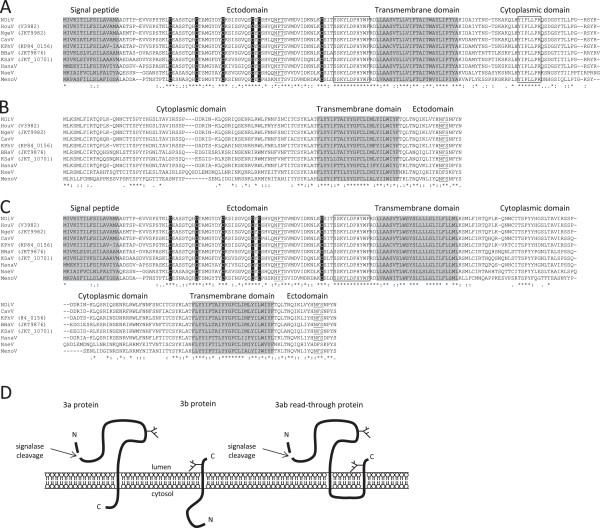
**Polypeptides encoded in the ORF3a/ORF3b region of mesonivirus genomes. (A)**. Clustal X alignment of the sequences of polypeptides encoded in ORF3a illustrating the predicted signal peptide, exodomain, transmembrane domain and cytoplasmic domain, two domains of highly conserved sequence (boxed), conserved cysteine residues (black shading) and a potential N-glycosylation site (underlined). **(B)**. Clustal X alignment of the sequences of polypeptides encoded in ORF3b illustrating the cytoplasmic domain, transmembrane domain and ectodomain and a potential N-glycosylation site (underlined). **(C)**. Clustal X alignment of the sequences of polypeptides that would be generated by -1 ribosomal frame-shift at the predicted ‘slippery’ sequence (CACUUUU) in the overlap region. **(D)**. Schematic representation of the predicted membrane topology of the putative 3a, 3b and 3ab glycoproteins using SignalP and TMHMM and NetNGlyc (http://www.expasy.org).

The third coding region, commencing immediately downstream of ORF2a, contains two overlapping long open reading frames (ORF3a and ORF3b) each encoding small hydrophobic proteins (Figure 4). A Clustal X alignment of the mesonivirus ORF3a proteins and individual structural analyses using SignalP and TMHMM and NetNGlyc (http://www.expasy.org) indicated that each is a class I transmembrane glycoprotein with a predicted N-termimal signal peptide, an ectodomain containing a conserved set of 6 cysteine residues and a single conserved N-glycosylation site, a transmembrane domain and a C-terminal cytoplasmic domain (Figure 4A, 4D). The cysteine-rich region of the ectodomain displays moderately high sequence conservation including a stretch of 13 totally conserved amino acids adjacent to the transmembrane domain that is unusually rich in large aromatic residues (Y, W, F). The cytoplasmic domain is highly variable in sequence, except for the completely conserved motif (H/S)YIPLLPR. No similar motif has been detected in a search of available eukaryote sequences. The ORF3b proteins are each predicted to be class II transmembrane proteins with an N-terminal cytoplasmic domain, a transmembrane domain and a short C-terminal ectodomain (Figure 4B, 4D). A single N-glycosylation site was predicted in the ectodomain of all the mesoniviruses, except NseV. The ORF3b proteins display poor overall sequence conservation and have few features that suggest a biological function.

The long 3’-terminal region of all mesoniviruses, except MenoV, contained a small open reading frame, which was previously designated as ORF4. A Clustal × multiple sequence alignment of the putative proteins indicated that, although they varied in size (45 aa to 61 aa), there was a remarkable level of sequence identity, particularly in the N-terminal portion of the protein (Additional file 4: Figure S4). However, there are no obvious structural characteristics that suggest a function and it is not known if these proteins are expressed in infected cells.

Various other short ORFs (<150 nt) were detected in the 3’-terminal regions of each mesonivirus but none was obviously conserved. Alternative ORFs of >180 nt (i.e., 60 amino acids) were also detected within other ORFs, including in ORF2a in the original strain of NDiV (72 aa) and the Ngewontan (72 aa) and Houston (61 aa) strains, in which the 5’ region is missing (Figure 2). These ORFs share a relatively high degree of nucleotide sequence conservation (92%) but a lower level of amino acid sequence conservation (79%) and no obvious structural characteristics indicate the possible function of the encoded proteins. Another short ORF that displayed some degree of sequence conservation was found in the 3’ region of ORF1a for all viruses except MenoV and KPhV (Figure 2).

### Transcription regulatory sequences

Mesoniviruses have been shown previously to express a 3’-nested set of polyadenylated sub-genomic mRNAs produced by copy-choice mediated leader-body fusion at sites located immediately upstream of alternative transcription regulatory sequence (TRS) elements which occur in the 5’-UTR and in the regions preceding ORF2a/ORF2b and ORF3a/ORF3b [5]. Each of the previously identified leader-body fusion sites and TRS elements [*AU*xx*UACUACUACUA* and *AGA*x(x)*ACUCUCCCA*] were completely conserved across all new mesoniviruses examined in this study.

### Ribosomal frame-shift sites

**Figure 5:**
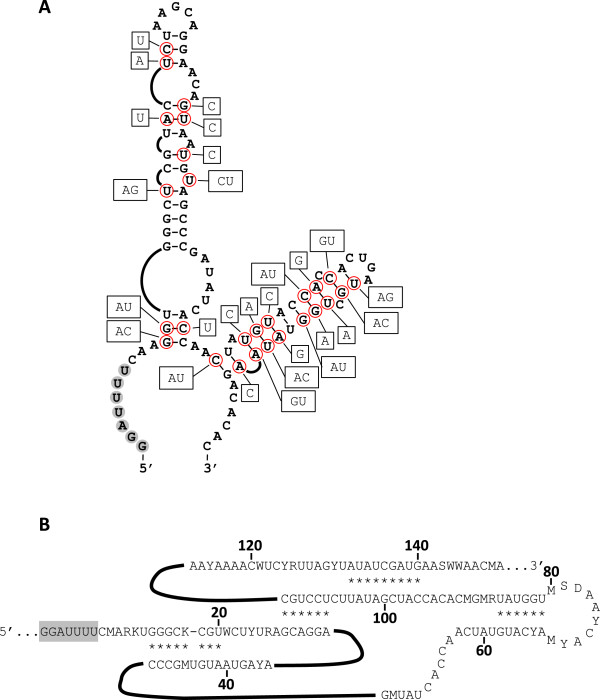
**Predicted structures of the mesonivirus ORF1a/ORF1b ribosomal frame-shift element (RFS) element. (A)**. Stem-loop structure predicted previously for NDiV [3] using pseudoknotsRG software, illustrating nucleotides that are substituted in various viruses (red circles). Nucleotide substitutions (which are primarily non-compensatory) are shown in the boxes. The corresponding sequence alignment is shown in Additional file 4: Figure S4. **(B)**. An alternative conserved pseudoknot structure predicted from the multiple sequence alignment using ipknot software. The putative ‘slippery’ sequence (GGAUUUU) at the ribosomal frame-shift site is shaded in grey.

In nidoviruses, translation of ORF1b characteristically occurs through a ribosomal frame-shift at a ‘slippery’ sequence in the ORF1a/ORF1b overlap region to allow read-through synthesis of a polyprotein (pp1ab). This is usually facilitated by an RNA pseudoknot in the sequence immediately downstream of the frame-shift site. However, a previous analysis of the NDiV sequence failed to identify a predicted pseudoknot structure, suggesting that a stem-loop structure (predicted using pknotsRG; http://bibiserv.techfak.uni-bielefeld.de/pknotsrg/) in the same region of the genome may facilitate the frame-shift [3]. However, our analysis using pknotsRG of the 13 new mesonivirus sequences and those of CavV, HanaV, NDiV, NseV and MenoV indicated that, although the slippery sequence (GGAUUUU) is completely conserved, the stem-loop structure predicted for NDiV is not predicted as the minimum free energy structure for any of the other viruses. Indeed, an alignment of the corresponding region of the genome of all mesoniviruses (Additional file 5: Figure S5) indicated that there was poor conservation of this sequence and few compensatory mutations that would preserve the stem-loop structure (Figure 5a). As functional pseudoknot structures are usually conserved, an alignment of all mesonivirus sequences extending 150 nt from the start of the ‘slippery sequence’ was analyzed using the IPknot server (http://rna.naist.jp/ipknot) which predicts the consensus secondary structure from a multiple sequence alignment (by using integer programming to select the maximum expected accuracy (MEA) structure) [10]. As shown in Figure 5b, the IPknot algorithm predicted a conserved pseudoknot structure, which conformed to all nucleotide substitutions in the available mesoniviruses.

Analysis of the region of the ORF3a/ORF3b overlap indicated that it has a similar format to that of the ORF1a/ORF1b overlap region, featuring a long overlap region and potential slippery sequence (CACUUUU) that could result in read-through by a -1 ribosomal frame-shift. Structural analyses using SignalP and TMHMM and NetNGlyc (http://www.expasy.org) indicated that a frame-shift at this site would result in a double-membrane-spanning glycoprotein (p3ab) of approximately 30–33 kDa (depending on whether one or two N-glysosylation sites are occupied) with both the N-terminal and C-terminal domains located in the extracellular lumen (Figure 4c, d). A previous analysis of CavV proteins by mass spectrometry identified peptide sequences from proteins migrating in gels at 17, 18 19 and 20 kDa that corresponded to the 3a protein (designated the M protein) and these were considered to be variously glycosylated forms; no protein corresponding to the ORF3b gene product was detected [5]. As for the ORF1a/ORF1b region, analysis of the genome region downstream of the putative ribosomal frame-shift element (RFS) element using pknotsRG identified various potential stem-loops but no commonly predicted minimum free energy structure. Analysis of a multiple sequence alignment of the region using the IPknot server also failed to predict a convincing pseudoknot structure.

## Discussion and conclusions

In this study we have identified 13 mesonivirus isolates and characterized their genome organization and phylogenetic relationships. Based on species demarcation criteria employed previously for mesoniviruses [1], five of these new isolates would be assigned to the same species as NDiV and CavV (*Alphamesonivirus-1*), other previously described mesoniviruses - HanaV MenoV, and NseV - would be assigned to three new species (*Alphamesonivirus-2, Alphamesonivirus-3* and *Alphamesonivirus-4*, respectively), and eight of the new isolates would represent three new species (*Alphamesonivirus-5, Alphamesonivirus-6* and *Alphamesonivirus-7*) [6]. However, we consider this basis for species demarcation, which employs only a genetic standard of pairwise sequence divergence to assign viruses to a species, should be re-evaluated following further assessment of the ecology of these viruses and their potential for genetic recombination which may provide a more informed analysis of suitable species demarcation criteria.

The isolates of viruses assigned to the species *Alphamesonivirus-1* illustrate the wide geographic distribution and mosquito host range of some mesoniviruses. The original four isolates of NDiV were made in northern and central Vietnam from *Culex vishnui* and *Cx. tritaeniorhynchus* mosquitoes collected indoors during a surveillance program for Japanese encephalitis virus (JEV) [3]. Our NDiV isolate from Java, Indonesia (Ngewontan strain) was also obtained from a pool of *Cx. vishnui* mosquitoes in 1981. The four NDiV isolates from Houston, Texas (Houston strain) were made from *Cx. quinquefasciatus* and *Aedes albopictus* collected outdoors within the Houston metropolitan area during West Nile virus (WNV) surveillance in 2004 and 2010, respectively. Interestingly, all of these isolates were from mosquitoes captured in or near human dwellings and from species that feed on humans. The close similarity of isolates from Houston and Vietnam may suggest a recent translocation, possibly during the Vietnam conflict when Houston hosted a major air base for embarkation/disembarkation. Isolates of Kampaeng Phet, Botang Baru and Karang Sari viruses, which may be considered three new mesonivirus species, have a geographic distribution extending at least from central Thailand to Kalimantan and Java in Indonesia, and have been isolated from at least two species of *Culex* mosquitoes.

Another consideration is the potential effect of mesonivirus infection on the susceptibility and vector competence of mosquitoes for viral pathogens of vertebrates. For example, both *Cx. vishnui* and *Cx. tritaeniorhynchus* are important vectors of JEV in Asia, and *Cx. quinquefasciatus* is the major vector of WNV in Houston. Recent experimental studies with *Ae. aegypti* mosquitoes infected with certain strains of the *Wolbachia* indicate that the presence of the symbiont bacterium interferes with dengue virus replication and decreases vector competence, possibly by upregulating or priming the mosquito’s innate immune system [11,12]. Similar results have been reported for *Wolbachia*-infected *Ae. aegypti* and chikungunya virus [11] and with *Wolbachia*-infected *Cx. quinquefaciatus* and WNV [13]. If a bacterial endosymboint can alter a mosquito’s vector competence for arboviruses, it seems plausible that a viral symbiont could have a similar effect [14]. This is an important area for future investigation.

Due to the continuous efforts of the virus discovery program of World Reference Center for Emerging Viruses and Arboviruses (WRCEVA), we have continued to isolate mesoniviruses from various insect vectors collected from widespread geographic locations (e.g., Nepal, Colombia and South Florida) suggesting that these viruses are more common than previously thought. Zirkel *et al*. [4] suggested that these viruses may have their origins in pristine rainforests and emergence may have been facilitated through anthropogenic-induced modifications (e.g., altered land use, deforestation).

The detailed analysis and comparison of mesonivirus genome architecture conducted here has revealed some unexpected characteristics. Firstly, the presence of block insertions of up to 588 nt in the 5’ terminal quadrant of ORF1a of several mesoniviruses has not been reported previously. The function of this region is presently unknown in mesoniviruses and other nidoviruses and so the structural and functional consequences of these insertions, which contain various imperfect repeats is unclear. Although sharing similar genome architecture, nidoviruses vary greatly in genome size. A previous analysis of the evolution of nidovirus genomes concluded that genome expansion has occurred in a wave-like fashion in which the three major coding regions (ORF1b, ORF1a and the 3’ORFs) expanded consecutively in a hierarchy that reflects the roles of their encoded proteins in the virus replication cycle [15]. This implies that nidoviruses have an inherent capacity for genome expansion, most likely associated with the transitional retention of sequences that serve as a resource for the evolution of new functions. The block insertions detected in ORF1a appear to be functionally redundant and their potential role in such evolutionary processes is presently unclear.

The comparative analysis also revealed that the stem-loop structure which had previously been identified at the RFS site of NDiV is not conserved in the other mesoniviruses and so may not be responsible for activating the -1 ribosomal frame shift. Secondary structure predictions using IPknot on the aligned sequences downstream of the conserved ‘slippery’ sequence site revealed a conserved pseudoknot structure that conformed to all nucleotide substitutions. However, the predicted structure featured only four relatively short regions of complementarity and no estimations of minimum free energy for the represented structures are available through this algorithm. A possible ‘slippery’ sequence (CACUUUU) was also detected in the ORF3a/ORF3b overlap region but no conserved stem-loop or pseudoknot structure was predicted by IPknot in the downstream sequence. It is, therefore, unclear whether ORF3b is expressed by internal initiation or as a read-through extension of ORF3a, which would generate a double-membrane spanning protein. Functional analysis of each of the regions corresponding to the RFS in ORF1a/ORF1b and the putative RFS in ORF3a/ORF3b would help resolve the mechanisms of mesonivirus gene expression.

In conclusion, we have identified and characterized several new mesoniviruses from mosquitoes of human medical importance sampled over time from widespread geographic regions. Several important questions related to their transmission, maintenance in insect hosts in nature, their potential impact of infection on the insect’s behavior, fertility, fecundity and survival, their evolution, their mechanisms of gene expression and their potential to be developed as biological control agents, warrant further investigation.

## Methods

### Cells and viruses

All viruses used in this study were obtained from the WRCEVA at the University of Texas Medical Branch. Some were isolated by the authors (RBT and HG), during arbovirus field studies; the remainder were isolated by other investigators and sent to the WRCEVA for identification and further characterization. All isolations were originally made in mosquito cell cultures (C6/36 or AP-61). The proposed names, original sources and geographic origins and GenBank Accession numbers of the sequences obtained for the 13 viruses included in our study are listed below and in Table 1.

**Table 1:** Origin of the 13 Mesoniviruses viruses described in this study

**Proposed name identification**	**Strain designation**	**Proposed virus species**	**GenBank accession no.**	**Host species**	**Collection locality**	**Collection date**
Ngewontan	JKT-9982	*Alphamesonivirus–1*	KC807169	*Culex vishnui*	Ngewontan, Java, Indonesia	1981
Houston	V3872	*Alphamesonivirus–1*	KC807175	*Culex quinquefasciatus*	Houston, Texas, USA	2004
Houston	V3982	*Alphamesonivirus–1*	KC807176	*Culex quinquefasciatus*	Houston, Texas, USA	2004
Houston	16740	*Alphamesonivirus–1*	KC807177	*Aedes albopictus*	Houston, Texas, USA	2010
Houston	16757	*Alphamesonivirus–1*	KC807178	*Aedes albopictus*	Houston, Texas, USA	2010
Bontag Baru	JKT-7774	*Alphamesonivirus–5*	KC807173	*Culex vishnui*	Bontag Baru, Kalmantan, Indonesia	1981
Bontag Baru	JKT 9891	*Alphamesonivirus–5*	KC807167	*Culex vishnui*	Karang Sari, Java, Indonesia	1981
Bontag Baru	JKT-9853	*Alphamesonivirus–5*	KC807168	*Culex tritaeniorhynchus*	Cilacap, Java, Indonesia	1981
Bontag Baru	JKT-9876	*Alphamesonivirus–5*	KC807166	*Culex vishnui*	Cilacap, Java, Indonesia	1981
Karang Sari	JKT-10701	*Alphamesonivirus–6*	KC807171	*Culex vishnui*	Karang Sari, Java, Indonesia	1981
Kamphaeng Phet	KP 84-0192	*Alphamesonivirus–7*	KC807174	Mosquito pool^a^	Kamphaeng Phet, Thailand	1985
Kamphaeng Phet	KP 84-0344	*Alphamesonivirus–7*	KC807172	Mosquito pool^a^	Kamphaeng Phet, Thailand	1984
Kamphaeng Phet	KP 84-0156	*Alphamesonivirus–7*	KC807170	Mosquito pool^a^	Kamphaeng Phet, Thailand	1984

^a^Species of the mosquito pool was not provided.

JKT-10701 was isolated from a pool of *Culex vishnui* mosquitoes collected on 11/26/1981 at Karang Sari, Cilacap (Central Java) Indonesia. The initial isolation was made at the Naval Medical Research Unit #2 (NAMRU-2) in Jakarta.

JKT-7774 was isolated at NAMRU-2 from a pool of 50 *Culex vishnui* collected at Bontag Baru, East Kalimantan, Indonesia in February 1981. Strains JKT-9876, JKT-9891 and JKT-9853 were also isolated at NAMRU-2 appear to be almost identical to isolate JKT-7774 in the phylogenetic tree. Since JKT-7774 was the first virus in this group to be isolated, it should be the prototype.

JKT-9876 was isolated at NAMRU-2 from a pool of *Culex vishnui* collected at Cilacap, Central Java, Indonesia on 10/13/1981.

JKT-9891 was isolated at NAMRU-2 from a pool of *Culex vishnui* mosquitoes collected on 10/14/1981 at Karang Sari, Cilacap (Central Java), Indonesia.

JKT-9853 was isolated at NAMRU-2 from a pool of *Culex tritaeniorhynchus* collected at Cilacap, Central Java, Indonesia on 10/6/1981.

KP84-0192, KP84-0344 and KP84- 0156 were isolated from pools of mosquitoes (species not given) collected at Kamphaeng Phet, Thailand in 1984. Original isolations were made at the Armed Forces Research Institute for Medical Sciences, Bangkok.

JKT-9982 was isolated at NAMRU-2 from a pool of *Culex vishnui* mosquitoes collected at Ngewotan, Central Java, Indonesia on 11/17/1981.

V3872 and V3982 – Both viruses were isolated at UTMB from pools of *Culex quinquefasciatus* mosquitoes collected in the summer of 2004 as part of an arbovirus surveillance program in Houston (Harris County), Texas, USA.

TVP16740 and TVP16757 – These two viruses were also isolated at UTMB from pools of *Aedes albopictus* mosquitoes collected in June 2010 as part of the Harris County arbovirus surveillance program in Houston, Texas.

Before sequencing, all virus stocks were grown in cultures of the C6/36 clone of *Ae. albopictus* cells [16], obtained from the American Type Culture Collection (ATCC), Manassas, VA. Infection was characterized by detachment of cells and cell lysis.

### Nucleotide sequence accession numbers

The following mesonivirus genome sequences were determined in this study: Karang Sari virus, strain JKT-10701 (KC807171); Bontang virus, strain JKT-9891 (KC807169); strain JKT-9853 (KC807167); strain JKT-9876 (KC807168); strain JKT-7774 (KC807166); Kamphang Phet virus, strain KP 84–0192 (KC807173); strain KP 84–0344 (KC807174); strain KP 84–0156 (KC807172); NDiV Ngewotan strain JKT-9982 (KC807170); NDiV Houston strain V3872 (KC807175), V3982 (KC807176), 16740 (KC807177), and 16757 (KC807178).

### Transmission electron microscopy

For ultrastructural analysis in ultrathin sections infected cells were fixed for at least 1 hr in a mixture of 2.5% formaldehyde prepared from paraformaldehyde powder, and 0.1% glutaraldehyde in 0.05 M cacodylate buffer pH 7.3 to which 0.03% picric acid and 0.03% CaCl_2_ were added. The monolayers were washed in 0.1 M cacodylate buffer, cells were scraped off and processed further as a pellet. The pellets were post-fixed in 1% OsO_4_ in 0.1 M cacodylate buffer pH 7.3 for 1 h, washed with distilled water and *en bloc* stained with 2% aqueous uranyl acetate for 20 min at 60°C. The pellets were dehydrated in ethanol, processed through propylene oxide and embedded in Poly/Bed 812 (Polysciences, Warrington, PA). Ultrathin sections were cut on Leica EM UC7 ultramicrotome (Leica Microsystems, Buffalo Grove, IL), stained with lead citrate and examined in a Philips 201 transmission electron microscope at 60 kV.

### Next generation sequencing

#### *Library construction*

Viral RNA (0.05-1.7 μg) was fragmented by incubation at 94°C for 8 min in 19.5 ul of fragmentation buffer (Illumina 15016648). First and second strand synthesis, adapter ligation and amplification of the library were performed using the Illumina TruSeq RNA Samplec Preparation kit under conditions prescribed by the manufacturer (Illumina). Samples were tracked using the “index tags” incorporated into the adapters as defined by the manufacturer.

#### *Sequence analysis*

Cluster formation of the library DNA templates was performed using the TruSeq PE Cluster Kit v3 (Illumina) and the Illumina cBot workstation using conditions recommended by the manufacturer. Paired end 50 base sequencing by synthesis was performed using TruSeq SBS kit v3 (Illumina) on an Illumina HiSeq 1000 using protocols defined by the manufacturer. Cluster density per lane was 645–980 k/mm^2^ and post filter reads ranged from 148–178 million per lane. Base call conversion to sequence reads wasperformed using CASAVA-1.8.2. Virus sequences were edited and assembled using the SeqMan and NextGen modules of the DNAStar Lasergene 7 program (Bioinformatics Pioneer DNAStar, Inc., Madison, WI). In certain cases, prefiltering of reads to remove host sequence enhanced the assembly process. Assembly was carried out using a fasta file of *Aedes albopictus* sequences to remove host DNA from the assembly thus reducing the number of contigs present.

#### *Genomic analysis*

The compiled sequences had their relationship to other viruses determined by a BLASTX search. The open reading frames were determined using Enzyme X (Nucleobytes, Inc., Aalsmeer). Relationships to the other two insect-specific nidoviruses were determined using MacVector (Cary, NC) using their DNA identity matrix software. The presence of conserved protein domains was determined using the SMART webserver [17,18].

### Phylogenetic analysis

A region corresponding to the full-length product encoded by ORF2a (unprocessed S proteins) was used to determine the relationships within the family *Mesoniviridae* along with a concatenated region of the highly conserved domains within ORF1ab (3CL^pro^, RdRp and ZnHel1). ORF2ab alignments were determined using the MUSCLE algorithm [19] as amino acids before being toggled back to the nucleotides while maintaining the alignment. Concatenated ORF1ab alignments were performed at the protein level. A maximum likelihood (ML) tree for ORF2a was constructed in MEGA 5.2 using the Jones-Taylor-Thornton substitution model of nucleotide substitution, uniform substitution rates among sites and the Nearest Neighbor-Interchange heuristic method and a very strong branch swap filter. An optimal ML tree was then estimated using the appropriate model and a heuristic search with tree-bisection-reconstruction branch swapping and 10 replicates, estimating variable parameters from the data, where necessary. A maximum likelihood phylogeny of the conserved domains of ORF1ab was constructed using PhyML 3.0 [20], the WAG + Gamma model of amino acid substitution, and the NNI + SPR method of branch swapping. One thousand bootstrap replicates were calculated for each dataset under the same models and expressed as a percentage.

Calculation of the PED between the highly conserved protein domains of ORF1ab (3CL^pro^, RdRp and ZnHel1) of the thirteen new isolates described in this study and those of five previously described mesoniviruses (CavV, HanaV, NDiV, NseV and MenoV) was performed using the ML method in the program TreePuzzle [21] and the WAG model of amino acid substitution. Sliding window analysis was used to calculate the mean amino acid divergences within and between the seven mesonivirus species described in this study. Percent divergences were calculated for each of the three largest ORFs in the genome (ORF 1ab, ORF2a, ORF2b) using the program SSE [22].

## Abbreviations

+ssRNA: single-stranded RNA; CavV: Cavally virus; DKNG: DakNong virus; HanaV: Hana virus; MenoV: Meno virus; DNiV: Nam Dinh virus; NseV: NSe virus; USA: United States of America; KPhV: Kamphang Phet virus; BBaV: Botang Baru virus; KSaV: Karang Sari virus; ORF: Open reading frame; 3CLpro: 3C-like protease; RdRp: RNA-dependent RNA polymerase; ZmHel1: Multinucleate zinc-binding module and associated helicase; ExoN: Exoribonuclease; NMT: N^7^-methyltransferase; OMT: 2’-*O*-methyltransferase; TRS: Transcription regulatory sequence; MEA: Maximum expected accuracy; RFS: Ribosomal frame-shift element; ML: Maximum-Likelihood; JEV: Japanese encephalitis virus; WNV: West Nile virus; WRCEVA: World Reference Center for Emerging Viruses and Arboviruses.

## Competing interests

The authors do hereby declare that they have no competing interests in this scientific work.

## Authors’ contributions

NV, HG, SGW, TGW, SLR, EG, RBT performed the laboratory experiments. NFL, PJW, CF performed the phylogenetic analyses; VP performed electron microscopy; PJW, KRB, CF performed the genomic analyses. NV, PJW, NFL, SGW, RBT contributed to final the manuscript preparation. All authors have read and approved the final manuscript.

## Supplementary Material

Additional file 1: Figure S1 Sliding window analysis of the pairwise amino acid distances within and between the seven putatively designated mesonivirus species for ORF1ab (replicase proteins), ORF2a (S) and ORF2b (N). Click here for file

Additional file 2: Figure S2 A Clustal X multiple sequence alignment of mesonivirus pp1ab polyproteins illustrating region containing the block insertions (yellow shading) and various imperfectly repeated sequences that occur at the boundary and within the blocks of inserted sequence. Click here for file

Additional file 3: Figure S3 A Clustal X multiple sequence alignment of the polypeptides encoded in ORF2a (S proteins) of the mesoniviruses. A predicted signal peptide in MenoV and predicted transmembrane domains in all mesoniviruses are shaded in aqua, predicted N-glycosylation sites are shaded in green, cysteine residues are shaded in yellow and the sites of proteolytic cleavage to generate glycoproteins S1 and S2 and the unidentified N-terminal fragment are shaded in purple. Click here for file

Additional file 4: Figure S4 A Clustal X multiple alignment of the sequences of putative polypeptides encoded on ORF4 which occurs in the 3’-terminal regions of all mesoniviruses except MenoV. To emphasize the alignment, the KSaV ORF4 protein has been shown to commence at the next available methionine residue located 34 amino acids downstream of the predicted initiation codon. Click here for file

Additional file 5: Figure S5 A Clustal X multiple sequence alignment of region immediately downstream of the ORF1a/ORF1b RFS that has been predicted previously to adopt a stem-loop structure in NDiV. The alignment illustrates sequence variations in nucleotides predicted in NDiV to be involved in base pairs that sustain the structure. Click here for file
