# Supplementary material for: Mesoniviruses are mosquito-specific viruses with extensive geographic distribution and host range
Source: Virol J. 2014 May 20;11:97. doi: 10.1186/1743-422X-11-97 (PMC4038087; doi:10.1186/1743-422X-11-97)

Supplementary Figure S1. Sliding window analysis of the pairwise amino acid distances within and between the seven putatively designated mesonivirus species for ORF1ab (replicase proteins), ORF2a (S) and ORF2b (N).

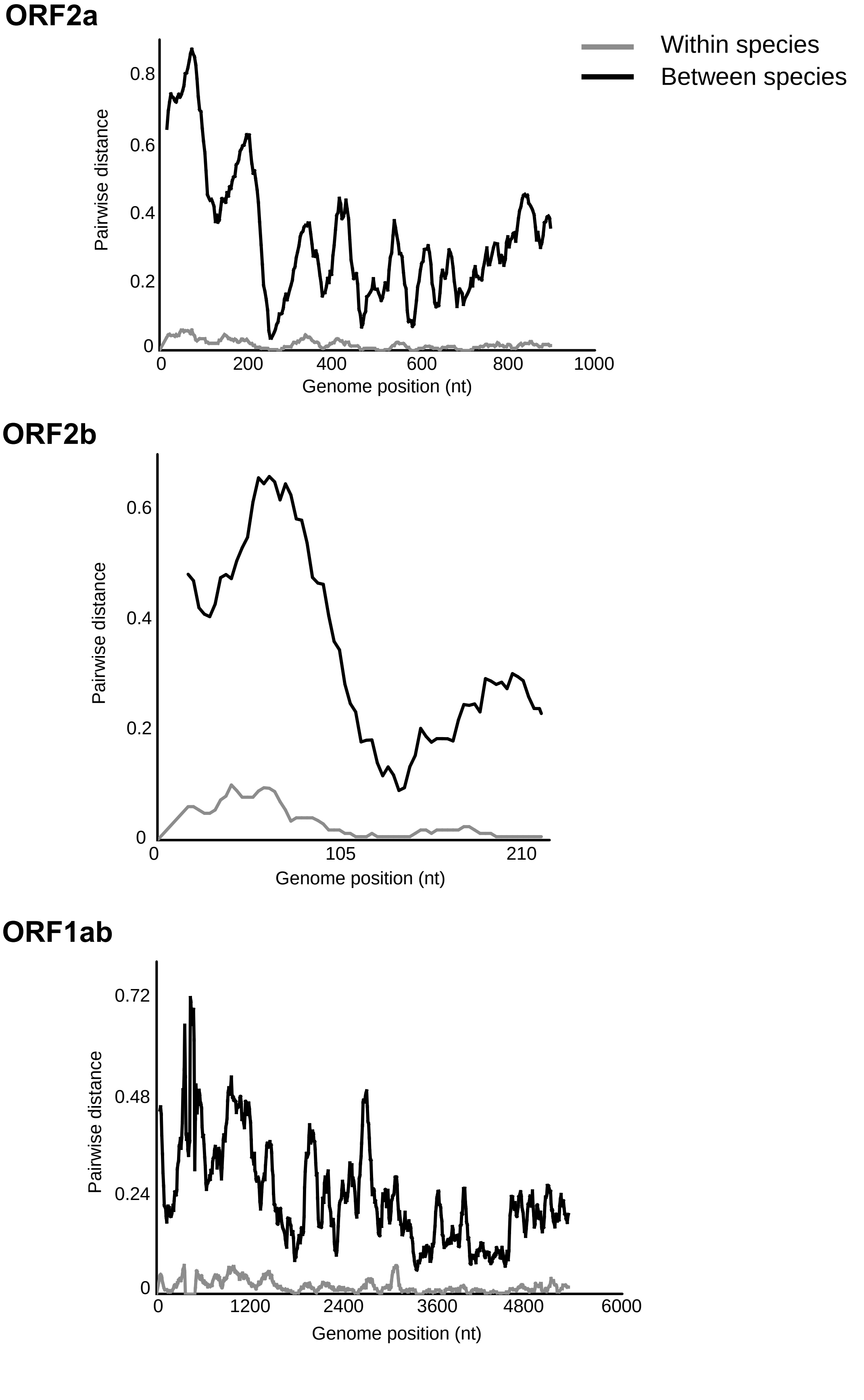

Supplement: Additional file 1: Figure S1 — Sliding window analysis of the pairwise amino acid distances within and between the seven putatively designated mesonivirus species for ORF1ab (replicase proteins), ORF2a (S) and ORF2b (N). [file 1743-422X-11-97-S1.pdf]
