# Supplementary material for: Mesoniviruses are mosquito-specific viruses with extensive geographic distribution and host range
Source: Virol J. 2014 May 20;11:97. doi: 10.1186/1743-422X-11-97 (PMC4038087; doi:10.1186/1743-422X-11-97)
Supplement: Additional file 2: Figure S2 — A Clustal X multiple sequence alignment of mesonivirus pp1ab polyproteins illustrating region containing the block insertions (yellow shading) and various imperfectly repeated sequences that occur at the boundary and within the blocks of inserted sequence. [file 1743-422X-11-97-S2.pdf]

Supplementary Figure S2. A Clustal X multiple sequence alignment of mesonivirus pp1ab polyproteins illustrating region containing the block insertions (yellow shading) and various imperfectly repeated sequences that occur at the boundary and within the blocks of inserted sequence

```
HouV (16757)       MTYHDYALKDNVVLKRDQKLALDNFVTEVFQFWTPVLTTLLLLA-YALRKIMHNPFVGPISDNPLKRALQWIIFVFTRRNLYYQTPVF
HouV (V3872)       MTYHDYALKDNVVLKRDQKLALDNFVTEVFQFWTPVLTTLLLLA-YALRKIMHNPFVGPISDNPLKRALQWIIFVFTRRNLYYQTPVF
HouV (16740)       MTYHDYALKDNVVLKRDQKLALDNFVTEVIQFWTPVLTTLLLLA-YALRKIMHNPFVGPISDNPLKRALQWIIFVFTRRNLYYQTPVF
HouV (V3982)       MTYHDYALKDNVVLKRDQKLALDNFVTEVIQFWTPVLTTLLLLA-YALRKIMHNPFVGPISDNPLKRALQWIIFVFTRRNLYYQTPVF
NDiV               MTYHDYALKDNVVLKRDQKLALDNFVTEVIQFWTPILTTLLLLA-YALRKIMHNPFVGPISDNPLKRALQWIIFVFTRRNLYYQTPVF
NgeV (JKT9982)     MTYHDYALKDNVVLKRDQKLALDNFVTEVIQFWTPILTTLLLLV-YALRKIMHNPFVGPISDNPLKRALQWIIFVFTRRNLYYQTPVF
CavV               MTYHDYALKDNAVLERDHKLALDNLVTNVIQYWTPILTMLLLAI-YILKKIMQNPFVGPVSDNPLKRALQWIIFVFTRRNLYYQTPVF
HanaV              MTYHDYALKDNVVLERDQQLAAENYVINVLSCLFAILTGLLVLP-YGL-KIVKNPFAGPVSDNFLKRFLQRIIFVFTRQYIYYQVPVF
KPhV (KP84_0156)   MTYHDYAFGDNVVLERDQAIEFECCIIKIFKFWLPLFLFMLFVFNNIIKHIIHYPFVGPVSDNVLKRFIQWITFVCTRRYMYYQVPNF
KPhV (KP84_0192)   MTYHDYAFGDNVVLERDQAIEFECCIIKIFKFWLPLFLFMLFVFNNIIKHIIHYPFVGPVSDNVLKRFIQWITFVCTRRYMYYQVPNF
KphV (KP84_0344)   MTYHDYAFGDNVVLERDQAIEFECCIIKIFKFWLPLFLFMLFVFNNIIKHIIHYPFVGPVSDNVLKRFIQWITFVCTRRYMYYQVPNF
BBaV (JKT9876)     MTYHEYAFGDNVVTERDHVLEVESFIIKIFKFWLPITLLMVSVYNNIIRRIICYSFVGPVSDNILKRFLQWIIFVFTRRYLYYQVPIF
BBaV (JKT9891)     MTYHEYAFGDNVVTERDHVLEVESFIIKIFKFWLPITLLMVSVYNNIIRRIICYSFVGPVSDNILKRFLQWIIFVFTRRYLYYQVPIF
BBaV (JKT9853)     MTYHEYAFGDNVVTERDHVLEVESFIIKIFKFWLPITLLMVSVYNNIIRRIICYSFVGPVSDNILKRFLQWIIFVFTRRYLYYQVPIF
BBaV (JKT7774)     MTYHEYAFGDNVVTERDHALEVESFIIKIFKFWLPITLLMVSVYNNIIRRIICYSFVGPVSDNILKRFLQWIIFVFTRRYLYYQVPIF
KSaV (JKT10701)    MTYHEYAFGDNVVTERDHALEVEGFIIKIFKFWLPIFLVMVFVYNNIIRRIMHYSFVGPVSDNVLKRFLQWIIFVFTRRYVYYQVPTF
NseV               MTYHDFANKDNVALARDQALATDAFYIKVFGIWTPITLAIIYLI----GHKLKNPFSGPVADNLLKRLLQKFIYLFTRKYLYYQIPSY
MenoV              MTYHDYAAYDNVVLQRDHITNTQTFYINMLTYWTPLSILSVFCI-YLLKRFMPNPLSGPVNDNTLKTLIQRIHYFFTRKYYYYQVPVY
                   ****::*  **..  **:    :    :::    .:    :        : :  .: **: ** **  :* : :. **:  *** * :

HouV (16757)       ARDESRLNIFLHNDFARLDRNTLNGYCKICNLYGHN-HTDKHNPTIDALVLAKTCKLLRYNDKVTKPLAYTVHNIRAYEKNTKTFADT
HouV (V3872)       ARDESRLNIFLHNDFARLDRNTLNGYCKICNLYGHN-HTDKHNPTIDALVLAKTCKLLRYNDKVTKPLAYTVHNIRAYEKNTKTFADT
HouV (16740)       ARDESRLNIFLHNDFARLDRNTLNGYCKICNLYGHN-HTDKHNPTIDALVLAKTCKLLRYNDKVTKPLAYTVHNIRAYEKNTKTFADT
HouV (V3982)       ARDESRLNIFLHNDFARLDRNTLNGYCKICNLYGHN-HTDKHNPTIDALVLAKTCKLLRYNDKVTKPLAYTVHNIRAYEKNTKTFADT
NDiV               ARDESRLNIFLHNDFARLDRNTLNGYCKLCNLYGHN-HTDKHNPTIDALVLAKTCKLLRYNDKVTKPLAYTVHNIRAYEKNTKTFADT
NgeV (JKT9982)     ARDESRLNIFLHNDFARLDRNTLNGYCKICNLYGHN-HTDKHNPTIDALVLAKTCKLLRYNDKVTKPLAYTVHNIRAYEKNTKTFADT
CavV               TRDESRLNVFLHNDFARLDRNTLNGYCKICNLYGHN-HTEKHNPTIDALVLAKTCKLLRYNDKVTKPLAYTVHNIRAYEKNTKTFADT
HanaV              ARDESRLNIFLHNDFARLDRNNLNGYCTKCNLYGHT-NTEKHNPTIDALVLAKTCKILRYNDKVTKPLAYTVHNIRAYEKNTKTFTDI
KPhV (KP84_0156)   ARDESRLNIFLHNDFARIDRNTLNGYCKICNLYGHT-HTDKHNPTIDALVLAKTCKLLRYNDKVTKPLAYTVHNIRAYEKNTKSFVET
KPhV (KP84_0192)   ARDESRLNIFLHNDFARIDRNTLNGYCKICNLYGHT-HTDKHNPTIDALVLAKTCKLLRYNDKVTKPLAYTVHNIRAYEKNTKSFVET
KphV (KP84_0344)   ARDESRLNIFLHNDFARIDRNTLNGYCKICNLYGHT-HTDKHNPTIDALVLAKTCKLLRYNDKVTKPLAYTVHNIRAYEKNTKSFVET
BBaV (JKT9876)     ARDESRLNIFLHNDFARLDRNTLTGYCNTCNLYGHT-DTDKHNPTIDALVLAKACKLIRYNDKVTKPLAYTVHNIRAYEKSTKIFVET
BBaV (JKT9891)     ARDESRLNIFLHNDFARLDRNTLTGYCNTCNLYGHT-DTDKHNPTIDALVLAKACKLIRYNDKVTKPLAYTVHNIRAYEKSTKIFVET
BBaV (JKT9853)     ARDESRLNIFLHNDFARLDRNTLTGYCNTCNLYGHT-DTDKHNPTIDALVLAKACKLIRYNDKVTKPLAYTVHNIRAYEKSTKIFVET
BBaV (JKT7774)     ARDESRLNIFLHNDFARLDRNTLTGYCNTCNLYGHT-DTDKHNPTIDALVLAKACKLIRYNDKVTKPLAYTVHNIRAYEKSTKIFVET
KSaV (JKT10701)    ARDESRFNIFLHNDFARLDRNTLNGYCNICNLYGHT-DTDKHNPTIDALVLAKTCKLIRYNDKVTKPLAYTVHNIRAYEKNTKAFVET
NseV               TRDEFRTNIFLSSDFARVDRTNLVGYCKKCNLYGHVDQSPKHTITFDSLILAKTCKLLSYNEKLSDLKTFTIHNIKSFEKNSKLFSEN
MenoV              ARDESRQNVFLTPDYARIDRVNLTEFCTRCNLYGHNTQSAKHVNTIDALILANVCKIRKFNKDLKQVQAYTVHNIKAFEHNPKLFTEI
                   :*** * *:**  *:**:** .*   :*. ******  .: **  *:*:*:**:.**:  :*..:..  ::*:***:::*:..* * :

HouV (16757)       FGTTTTNIPTKYALAPKKAVSELTTIESNLGPIYVNNTIAYPHLGFIAYDNKQHLQELLANVTVVLDTLMVYTQYELDDAT-MNIRKS
HouV (V3872)       FGTTTTNIPTKYALAPKKAVSELTTIESNLGPIYVNNTIAYPHLGFIAYDNKQHLQELLANVTVVLDTLMVYTQYELDDAT-MNIRKS
HouV (16740)       FGTTTTNIPTKYALAPKKAVSELTTIESNLGPIYVNNTIAYPHLGFIAYDNKQHLQELLANVTVVLDTLMVYTQYELDDAT-MNIRKS
HouV (V3982)       FGTTTTNIPTKYALAPKKAVSELTTIESNLGPIYVNNTIAYPHLGFIAYDNKQHLQELLANVTVVLDTLMVYTQYELDDAT-MNIRKS
NDiV               FGTTTTNIPTKYALAPKKAVSELTTIESNLGPIYVNNTIAYPHLGFIAYDNKQHLQELLANVTVVLDTIMVYTQYELDDAT-MNIRKS
NgeV (JKT9982)     FGTTTTNIPTKYALAPKKAVSELTTIESNLGPIYVNNTIAYPHLGFIAYDNKQHLQELLANVTVVLDTLMVYTQYELDDAT-MNIRKS
CavV               FGTTTTNIPTKYALAPKKAVADLTTIESNLGPIYVNNTIAYPHLGFIAYNNKQHLQELLANVTVVLDTVMVYTQYELDDAT-INIRKS
HanaV              FGNTTTNIPTKYALAPKKALCNLNSIESNLGPIYVNNTIAYPHLGLIAYNDKQHLQELLANLAVVLDTFMVYTQYELDDTT-INVRKS
KPhV (KP84_0156)   FGTNTTNIPTKYALAPKKAVSGLTTIESNLGPIYVNNTIAYPHLGFIAYDNKQHLQELLANLTIVLDTFMVYTQYELDDTT-INIRKS
KPhV (KP84_0192)   FGTNTTNIPTKYALAPKKAVSGLTTIESNLGPIYVNNTIAYPHLGFIAYDNKQHLQELLANLTIVLDTFMVYTQYELDDTT-INIRKS
KphV (KP84_0344)   FGTNTTNIPTKYALAPKKAVSGLTTIESNLGPIYVNNTIAYPHLGFIAYDNKQHLQELLANLTIVLDTFMVYTQYELDDTT-INIRKS
BBaV (JKT9876)     FGTNTTNIPTKYALAPKKAVCELTTIESNLGPIYVNNTIAYPHLGFIAYDNKQQLQDLLANLTIVLDTFMVYTQHELDDTT-INIRKS
BBaV (JKT9891)     FGTNTTNIPTKYALAPKKAVCELTTIESNLGPIYVNNTIAYPHLGFIAYDNKQQLQDLLANLTIVLDTFMVYTQHELDDTT-INIRKS
BBaV (JKT9853)     FGTNTTNIPTKYALAPKKAVCELTTIESNLGPIYVNNTIAYPHLGFIAYDNKQQLQDLLANLTIVLDTFMVYTQHELDDTT-INIRKS
BBaV (JKT7774)     FGTNTTNIPTKYALAPKKAVCELTTIESNLGPIYVNNTIAYPHLGFIAYDNKQQLQDLLANLTIVLDTFMVYTQHELDDTT-INIRKS
KSaV (JKT10701)    FGTNTTNIPTKYALAPKKAVCELTTIASNLGPIYVNNTIAYPHLGFIAYDNKQHLQELLANLTIVLDTLMVYTQYELDDTT-INIRKS
NseV               FGNPTTIIPSKFALAPKAAVSQLTTIEANIGLIYVNNTAQYPHLGFIAYNNQEQLEQQVAILSTVIDNLMVYTTYSINDVTTMNLHTS
MenoV              FGTPVSIIPSRFALAPKSTLATLKPKSTNLGQIYINDTIEHPHIGFFAYENQEQLNDLIAQLSLVLDHLMVYTDLPIPDVPTMQLYKD
                   **. .: **:::***** ::. *..  :*:* **:*:*  :**:*::**:::::*:: :* :: *:* .****   : *.. ::: ..

HouV (16757)       DITLSFVNDFDLTNALTNELKDPRTPWLLKAKKTSNKASEQDDDTEAEDTQKNKRK--------------------------------
HouV (V3872)       DITLSFVNDFDLTNALTNELKDPRTPWLLKAKKTSNKASEQDDDTEAEDTQKNKRK--------------------------------
HouV (16740)       DITLSFVNDFDLTNALTNELKDPRTPWLLKAKKTSNKASEQDDDTEAEDTQKNKRK--------------------------------
HouV (V3982)       DITLSFVNDFDLTNALTNELKDPRTPWLLKAKKTSNKASEQDDDTEAEDTQKNKRK--------------------------------
NDiV               DITLSFVNDFDLTNALTNELKDPRTPWLLKAKKTSNKASEQDDDTEAEDTQKNKRK--------------------------------
NgeV (JKT9982)     DITLSFVNDFDLTNALTNELKDPRTPWLLKAKKTSNKASEQDDDTEAEDTQKNKCK--------------------------------
CavV               NIKLHFVNDFDLTNALTNELKDPRTPWLLKAKKSSNKALEIEDDTEAEETQKTKRK--------------------------------
HanaV              AIRLSFENDFDLTNALTNEMKDPRTPWLLKAKKTSNKSLELEDDTEAEEPQ-PKRK--------------------------------
KPhV (KP84_0156)   NVILNFVNDFDLTCALNNELKDPRTPWLLKAKKGSNKSAEIEDDTEAEETLKSKRKGQSGEFSKTSKSRKGN----------------
KPhV (KP84_0192)   NVILNFVNDFDLTCALNNELKDPRTPWLLKAKKGSNKSAEIEDDTEAEETLKSKRKGQSGEFSKTSKSRKGN----------------
KphV (KP84_0344)   NVILNFVNDFDLTCALNNELKDPRTPWLLKAKKGSNKSAEIEDDTEAEETLKSKRKGKSGEFSKTSKSRKGN----------------
BBaV (JKT9876)     NVMLNFVKDFDLTHALNNELKDPRTPWLLKAKKCSSKSTKLDDDTEAEDNQKFKRKGKADEFEKSPKPRKGNEAVKAAEMPKQRKGNS
BBaV (JKT9891)     NVMLNFVKDFDLTHALNNELKDPRTPWLLKAKKCSSKSTKLDDDTEAEDNQKFKRKGKADEFEKSPKPRKGNEAVKAAEMPKQRKGNS
```

```
BBaV (JKT9853)     NVMLNFVKDFDLTHALNNELKDPRTPWLLKAKKCSSKSTKLDDDTEAEDNQKFKRKGKADEFEKSPKPRKGNEAVKAAEMPKQRKGNS
BBaV (JKT7774)     NVMLNFVKDFDLTHALNNELKDPRTPWLLKAKKCSSKSTKLDDDTEAEDNQKFKRKGKADEFEKSPKPRKGNEAVKAAEMPKQRKGNS
KSaV (JKT10701)    NILLTFVKDFDLTHALNNELKDPRTPWLLKAKKGSNKSTELEDDTEAEDNQKSKRKGKVGEAEKSPKTRKGNEAVKAEAMPKQRKANS
NseV               VEELTFTNDTDTLTALQNELKDPRSAWLLKAKKVTNRVVDNEDEATDQEHQQKPRR--------------------------------
MenoV              SSLLHFTNNTNLLCAFTSYQKEARTNWLFKEVKSTKVDDEDEHNLEKQEIQLKPQTN-------------------------------
                      * * :: :*  *: .  *:.*: **:*  * :.   . :.:   ::

HouV (16757)       ----------------------------------------------------------------------------------------
HouV (V3872)       ----------------------------------------------------------------------------------------
HouV (16740)       ----------------------------------------------------------------------------------------
HouV (V3982)       ----------------------------------------------------------------------------------------
NDiV               ----------------------------------------------------------------------------------------
NgeV (JKT9982)     ----------------------------------------------------------------------------------------
CavV               ----------------------------------------------------------------------------------------
HanaV              ----------------------------------------------------------------------------------------
KPhV (KP84_0156)   -------------------------------------------------------------------------NTQNSGDAETPETSK
KPhV (KP84_0192)   -------------------------------------------------------------------------NTQNSGDAETPETSK
KphV (KP84_0344)   -------------------------------------------------------------------------NTQNSGDAETPETSK
BBaV (JKT9876)     DNEKAESIPQPRKGNPHAVKALEPQPRNEIPDAIKAEEQQPAKDVLEAVKAEESTELHPEAQDAEKAEEKMEQSTDNPEAVKAEETQA
BBaV (JKT9891)     DNEKAESIPQPRKGNPHAVKALEPQPRNEIPDAIKAEEQQPAKDVLEAVKAEESTELHPEAQDAEKAEEKMEQSTDNPEAVKAEETQA
BBaV (JKT9853)     DNEKAESIPQPRKGNPHAVKALEPQPRNEIPDAIKAEEQQPAKDVLEAVKAEESTELHPEAQDAEKAEEKMEQSTDNPEAVKAEETQA
BBaV (JKT7774)     DNEKAESIPQPRKGNPHAVKALEPQPRNEIPDAIKAEEQQPAKDVLEAVKAEESTELHPEAQDAEKAEEKMEQSTDNPEAVKAEETQA
KSaV (JKT10701)    EAIDAEDLPKSRKGNSDAVKASEPQPRKEI-DTVKADG----PHIPQAVKVEKSTELLSETNDAEKAEEKTEQSTENLEAVKAEENPA
NseV               ----------------------------------------------------------------------------------------
MenoV              ----------------------------------------------------------------------------------------

HouV (16757)       --------------------------------------------------------------------------------GKQLKPQT
HouV (V3872)       --------------------------------------------------------------------------------GKQLKPQT
HouV (16740)       --------------------------------------------------------------------------------GKQLKPQT
HouV (V3982)       --------------------------------------------------------------------------------GKQLKPQT
NDiV               --------------------------------------------------------------------------------GKQLKPQT
NgeV (JKT9982)     --------------------------------------------------------------------------------GKQLKPQT
CavV               --------------------------------------------------------------------------------GK-LQPQT
HanaV              --------------------------------------------------------------------------------GKKLKPQT
KPhV (KP84_0156)   TTTDTSEN--------------------------------------------ATDVQNGPQ-----NASAST-KQ-SKRKGK-LTPQT
KPhV (KP84_0192)   TTTDTSEN--------------------------------------------ATDVQNGPQ-----NASAST-KQ-SKRKGK-LTPQT
KphV (KP84_0344)   TTTDTSEN--------------------------------------------ATDVQNGPQ-----NASAST-KQ-SKRKGK-LTPQT
BBaV (JKT9876)     PNSEKSEEPAQTIQTSTANLECSEHAQ--TAETSKPAPVA-EAKKAKPGTSKNKSVENEKP-----NAPSTTQHQ-SKRKGKTLAPQT
BBaV (JKT9891)     PNSEKSEEPAQTIQTSTANLECSEHAQ--TAETSKPAPVA-EAKKAKPGTSKNKSVENEKP-----NAPSTTQHQ-SKRKGKTLAPQT
BBaV (JKT9853)     PNSEKSEEPAQTIQTSTANLECSEHAQ--TAETSKPAPVA-EAKKAKPGTSKNKSVENEKP-----NAPSTTQHQ-SKRKGKTLAPQT
BBaV (JKT7774)     PNSEKSEEPAQTIQTSTANLECSEHAQN-TAETSKPAPVA-EAKKAKPGTSKNKSVENE-------NAPSTTQHQ-SKRKGKTLAPQT
KSaV (JKT10701)    PISEPSEKPAQISQTSAAVVQCSEHAQTNSELQSAPVTVAKEQNKATPGTSKNTPVANEKPTSGKSNAPATKYKQTSKRKGKQLTPQT
NseV               --------------------------------------------------------------------------------ERTLKSEN
MenoV              --------------------------------------------------------------------LLKYKTSKIGKSKARLPAEE
                                                                                      * .:

HouV (16757)       QLLQHTLAKQTKYARRQ----PFLSFGPTYMTLLCLISIMSPTYATVCTTYEPLDQADLYCNNLQNLTIAKYHAYANYEQLNRQCFSI
HouV (V3872)       QLLQHTLAKQTKYARRQ----PFLSFGPTYMTLLCLISIMSPTYATVCTTYEPLDQADLYCNNLQNLTIAKYHAYANYEQLNRQCFSI
HouV (16740)       QLLQHTLAKQTKYARRQ----PFLSFGPTYMTLLCLISIMSPTYATVCTTYEPLDQADLYCNNLQNLTIAKYHAYANYEQLNRQCFSI
HouV (V3982)       QLLQHTLAKQTKYARRQ----PFLSFGPTYMTLLCLISIMSPTYATVCTTYEPLDQADLYCNNLQNLTIAKYHAYANYEQLNRQCFSI
NDiV               QLLQHTLAKQTKFARRQ----PFLSFGPTYMTLLCLISIMSPTYATVCTTYEPLDQADLYCNNLQNLTIEKYHAYANYEQLNRQCFSI
NgeV (JKT9982)     QLLQHTLAKQTKFARRQ----PLLSFGPTYMTLLCLISIMSPTYATVCTTYEPLDQADLYCNNLQNLTIAKYHAYANYEQLNRQCFSI
CavV               QLLQHTLAKQSKPARRQ----SHLTFGPAYMTMLCLISIMSPTQAKVCTTYEILDQADLYCNNLQNLTIAKYHAYANYEHMNKQCFST
HanaV              KLLQHTLAKQNKTARNQ----TPLGFGPTFMTMLCLTCILTSTQAKICTEYDTISQSDVYCNTVQNLTISKYHAYANYEHLNRQCFST
KPhV (KP84_0156)   QLLDHTLN-QTRLAHRT----PYLKFGPTYMTLLFLTFVMIPAQATICTYYETVSQDNQYCEYVQNVTSSKYLAYAHYEHLNTKCFST
KPhV (KP84_0192)   QLLDHTLN-QTRLAHRT----PYLKFGPTYMTLLFLTFVMIPAQATICTYYETVSQDNQYCEYVQNVTSSKYLAYAHYEHLNTKCFST
KphV (KP84_0344)   QLLDHTLN-QTRLAHRT----PYLKFGPTYMTLLFLTFVMIPAQATICTYYETVSQDNQYCEYVQNVTSSKYLAYAHYEHLNTKCFST
BBaV (JKT9876)     QLLNHTLNQKKRPARPR----PYLKFGPTYMALLFLTSVLIPTHAMICTRYETVPQSEQYCEYVQNITISKYLAYANYEHLNKKCFST
BBaV (JKT9891)     QLLNHTLNQKKRPARPR----PYLKFGPTYMALLFLTSVLIPTHAMICTRYETVPQSEQYCEYVQNITISKYLAYANYEHLNKKCFST
BBaV (JKT9853)     QLLNHTLNQKKRPARPR----PYLKFGPTYMALLFLTSVLIPTHAMICTRYETVPQSEQYCEYVQNITISKYLAYANYEHLNKKCFST
BBaV (JKT7774)     QLLNHTLNQKKRPARPR----PYLKFGPTYMALLFLTSVLIPTHAMICTRYETVPQSEQYCEYVQNITISKYLAYANYEHLNKKCFST
KSaV (JKT10701)    QLLNHTLANQNRPARIR----PYLRFGPTFMSLLFLTSVLIPTQAMICTRYVTVPQSDQYCEHVQDLTIANYHAYANYEHLNSKCFST
NseV               QLLQHTNAKAPKIEKFKHRQAPRLMFGPTFMSMLFMLSIINTTYSTICTQYQLAAEADVYCDQVKNLT-QEYLPYYNYEQALKQCFSV
MenoV              STTENPQGKQTKIGVNP----VRVAFGPSFMTLLCIFALVQTSFSKICTEYHNTNEDGIPCQLVNNLTNTKYMAFYNYEQVKKPCFSL
                   .  ::.     :           : ***::*::* :  :: .: : :** *    :    *: ::::*  :* .: :**:    ***

HouV (16757)       DGAEFKDLIRLSVSN-ALNLNNVIKPVPRADYILKAFSNALPLNTHVLSDYNTILDLQILMQFYNLNGS---NVLYTETYSESEDYAG
HouV (V3872)       DGAEFKDLIRLSVSN-ALNLNNVIKPVPRADYILKAFSNALPLNTHVLSDYNTILDLQILMQFYNLNGS---NVLYTETYSESEDYAG
HouV (16740)       DGAEFKDLIRLSVSN-ALNLNNVIKPVPRADYILKAFSNALPLNTHVLSDYNTILDLQILMQFYNLNGS---NVLYTETYSESEDYAG
HouV (V3982)       DGAEFKDLIRLSVSN-ALNLNNVIKPVPRADYILKAFSNALPLNTHVLSDYNTILDLQILMQFYNLNGS---NVLYTETYSESEDYAG
NDiV               DGAEFKDLIRLSVSN-ALNLNNVIKPVPRDDYILKAFSNALPLNTHVLSDYNTILDLQILMQFYNLNGS---NVLYTETYSESEDYAG
NgeV (JKT9982)     DGAEFKDLIRLSVSN-ALNLNNVIKPVPRADYILKAFSNALPLNTHVLSDYNTILDLQILMQFYNLNGS---NVLYTETYSESEDYAG
CavV               DGAEFKDLVRLSVSN-ALNLNNVIKPLPKDDYILKAFSNALPLNTHVLSDYTTILDLQILMQFYNLNGS---NVLYTENYSESEDYTG
HanaV              DGVEFKDLIRLSVSN-VLNLNNIIKPTLKDDYILKAFSNALPLNTYVLSDYNTFQDLQILMQFYNLNSS---TVVYTENYSASENYVG
KPhV (KP84_0156)   DGAEYKDLVRLSVSN-VLNLSNLIKPIPKDDYILKAFTDALPLKTHILSDYNTKLDLQILMQFYNLNGS---NVVYVETYSESEDYDG
KPhV (KP84_0192)   DGAEYKDLVRLSVSN-VLNLSNLIKPIPKDDYILKAFTDALPLKTHILSDYNTKLDLQILMQFYNLNGS---NVVYVETYSESEDYDG
KphV (KP84_0344)   DGAEYKDLVRLSVSN-VLNLSNLIKPIPKDDYILKAFTDALPLKTHILSDYNTKLDLQILMQFYNLNGS---NVVYVETYSESEDYDG
BBaV (JKT9876)     DGAEFKDLIRLSVSN-VLNLSNLIKPIPKDDYILKAFSNALPLKTHILSDFNTKLDLQILMQFYNLSRE---NVVYAENYSESEDYVG
```

```
BBaV (JKT9891)     DGAEFKDLIRLSVSN-VLNLSNLIKPIPKDDYILKAFSNALPLKTHILSDFNTKLDLQILMQFYNLSRE---NVVYAENYSESEDYVG
BBaV (JKT9853)     DGAEFKDLIRLSVSN-VLNLSNLIKPIPKDDYILKAFSNALPLKTHILSDFNTKLDLQILMQFYNLSRE---NVVYAENYSESEDYVG
BBaV (JKT7774)     DGAEFKDLIRLSVSN-VLNLSNLIKPIPKDDYILKAFSNALPLKTHILSDFNTKLDLQILMQFYNLSRE---NVVYAENYSESEDYVG
KSaV (JKT10701)    DGAEFKDLIRLSVSN-VLNLSNVIKPIPKDDYILKAFSDALPLKTHVLSDFNTKLDLQILMQFYNLSGS---NVVYAENYSESEDYAG
NseV               DGFNYVDLIRLSVNKPQLNLRNVLKPEINSD-IHKALTNTLPFNTYILSTFETYLDIQILLNYYNINISE-SNILFTEQHTYTKEYAG
MenoV              DGNNYEPLVRLSMQTTDLNLNNIIKPQPIDEYLLKAFYGLLPLNTFVLSTLETKTDLQILAEVYNINTTDNSKIVYNTPNTIRSDYVG
                   ** ::  *:***:..  *** *::**    : : **: . **::*.:**   *  *:*** : **:.     .:::    :  .:* *
HouV (16757)       KVVQLLAQGTGGICKAPACILFTGLATTATDVEVKVTERLTKRIKHQEHGKPLYINPSCRKTCYCMHKPEVKPEPIETVKYAPQAEFY
HouV (V3872)       KVVQLLAQGTGGICKAPACILFTGLATTATDVEVKVTERLTKRIKHQEHGKPLHINPSCRKTCYCMHKPEVKPEPIETVKYAPQAEFY
HouV (16740)       KVVQLLAQGTGGICKAPACILFTGLATTATDVEVKVTERLTKRIKHQEHGKPLHINPSCRKTCYCMHKPEVKPEPIETVKYAPQAEFY
HouV (V3982)       KVVQLLAQGTGGICKAPACILFTGLATTATDVEVKVTERLTKRIKHQEHGKPLHINPSCRKTCYCMHKPVVKPEPIETVKYAPQAEFY
NDiV               KVVQLLAQGTGGICKAPACILFTGLATTVTDVEVKVTERLTKRIKHQEHGKPLHINPSCRKTCYCMYKPKVKPEPVETVKYAPQAEFY
NgeV (JKT9982)     KVVQLLAQGTGGICKAPACILFTGLATTATDVEVKVTERLTKRIKHQEHGKPLHINPSCRKTCYCMHKPEVKPEPIETVKYAPQAEFY
CavV               KVIQLLAQGTGGICKAPACIKFTGLDPTATDVEVKITERLTKRIKHQEHGKSLIIDPSCRKTCYCMRKPEIKPEPVEPVKYAPPAEFY
HanaV              KVVQLLAQGTGGICAAPTCILFTGLATTVTDVEIKVTERLTKRIKHQEHGKPLFVDPSCKTTCTCMDKPKVEPITIEPVKYAPHADFY
KPhV (KP84_0156)   KVVQLLAQSTGDICVKPTCILFTGLDTTVRDVDVKVTERLVKRIKHQEHGKPLHIKPSCSKKCYCMYKPEIEPEIVEIVKFTPQADFY
KPhV (KP84_0192)   KVVQLLAQSTGDICVKPTCILFTGLDTTVRDVDVKVTERLVKRIKHQEHGKPLHIKPSCSKKCYCMYKPEIEPEIVEIVKFTPQADFY
KphV (KP84_0344)   KVVQLLAQSTGDICVKPTCILFTGLDTTVRDVDVKVTERLVKRIKHQEHGKPLHIKPSCSKKCYCMYKPEIEPEIVEIVKFTPQADFY
BBaV (JKT9876)     KVVQLLAQSTGDICKKPTCILFTGLETTPKDVDVKVTKRLTKRVKHQEHGKQLIIDPFCSKKCECMNKPEMKPEIKETAKFAPQADFY
BBaV (JKT9891)     KVVQLLAQSTGDICKKPTCILFTGLETTPKDVDVKVTKRLTKRVKHQEHGKQLIIDPFCSKKCECMNKPEMKPEIKETAKFAPQADFY
BBaV (JKT9853)     KVVQLLAQSTGDICKKPTCILFTGLETTPKDVDVKVTKRLTKRVKHQEHGKQLIIDPFCSKKCECMNKPEMKPEIKETAKFAPQADFY
BBaV (JKT7774)     KVVQLLAQSTGDICKKPTCILFTGLETTPKDVDVKVTKRLTKRVKHQEHGKQLIIDPFCSKKCECMNKPEMKPEIKETAKFAPQADFY
KSaV (JKT10701)    KVVQLLAQSTGDICDKPTCILFTGLETTPKDVEVKVTERLMKRVKHQEHGKQLIINPSCSKQCECMNKPTIEPEIKETVKFAPLADFY
NseV               RIVQLTTQSTEISCQLPSCIYYHGFTHEPLDLEINITTRVTKRIQHQAEGTPIQINPACKETCTCMN--KIEPEPVKPLLLAPPREFY
MenoV              KILQLSDKVT-FDCEAPTCILFSGLNFNASALDIKITNHLTRRIEHSTTGRPLIISPECKQICSCSMQSTVKQ---EPEVINPSQDFY
                   :::**  : *   *  *:** : *:      :::::* :: :*::*.  *  : :.* *    * *     ::    :     *  :**

HouV (16757)       TQLRYFQNHELQMYDDFEMGVLRYNNYTLNTFIYSNETCILT--RGVHCVYNPEHFAITRVYNNLGNYLECGVNQEFCESLQQEFMFN
HouV (V3872)       TQLRYFQNHELQMYDDFEMGVLRYNNYTLNTFIYSNETCILT--RGVHCVYNPEHFAITRVYNNLGNYLECGVNQEFCESLQQEFMFN
HouV (16740)       TQLRYFQNHELQMYDDFEMGVLRYNNYTLNTFIYSNETCILT--RGVHCVYNPEHFAITRVYNNLGNYLECGVNQEFCESLQQEFMFN
HouV (V3982)       TQLRYFQNHELQMYDDFEMGVLRYNNYTLNTFIYSNETCILT--RGVHCVYNPEHFAITRVYNNLGNYLECGVNQEFCESLQQEFMFN
NDiV               TQLRYFQNHELQMYDDFEMGVLRYNNYTLNTFIYSNETCILT--RGVHCVYNPEHFAITRVYNNLGNYLECGVNQEFCESLQQEFMFN
NgeV (JKT9982)     IQLRYFQNHELQMYDDFEMGVLRYNNYTLNTFIYSNETCILT--RGVHCVYNPEHFAITRVYNNLGNYLECGVNQEFCESLQQEFMFN
CavV               TQLRYFQNHELRMYDDFEMGVLRYNNYTLSTFIYSNDTCIQT--RGVHCVYNSEHFVITRVYNNLGNYLECGVNQEFCESLQQEFMFN
HanaV              TQLRYFQNYELRIYDDFEMGVLRYNNYTLNTFIYSNETCILP--HGHHCIYNLENFETTRVYNHLGNHLECGVHQEFCESLQQEFMYN
KPhV (KP84_0156)   TQLSYFQNYELRMYDDFDAGVLRYNNYTLTNFVYNNDTCLLQ--LNHRCAYNLEHFEITRVYNDIGNFLECGVNQEFCESLQQEFLFT
KPhV (KP84_0192)   TQLSYFQNYELRMYDDFDAGVLRYNNYTLTNFVYNNDTCLLQ--LNHRCAYNLEHFEITRVYNDIGNFLECGVNQEFCESLQQEFLFT
KphV (KP84_0344)   TQLSYFQNYELRMYDDFDAGVLRYNNYTLTNFVYNNDTCLLQ--LNHRCAYNLEHFEITRVYNDIGNFLECGVNQEFCESLQQEFLFT
BBaV (JKT9876)     TQLSYFQNYELRMYDDFDMGVLRYNNYTLTTFVYNNATCLLN--LKHRCVYNPEHFVTTRVYNDIGNYLECGINQEFCESLQQEFTFS
BBaV (JKT9891)     TQLSYFQNYELRMYDDFDMGVLRYNNYTLTTFVYNNATCLLN--LKHRCVYNPEHFVTTRVYNDIGNYLECGINQEFCESLQQEFTFS
BBaV (JKT9853)     TQLSYFQNYELRMYDDFDMGVLRYNNYTLTTFVYNNATCLLN--LKHRCVYNPEHFVTTRVYNDIGNYLECGINQEFCESLQQEFTFS
BBaV (JKT7774)     TQLSYFQNYELRMYDDFDMGVLRYNNYTLTTFVYNNATCLLN--LKHRCVYNPEHFVTTRVYNDIGNYLECGINQEFCESLQQEFTFS
KSaV (JKT10701)    TQLSYFQNYELRMYDDFDMGVLRYNNYTLTTFVYNNATCLLN--LKHRCVYNPEHFEITRVYNDIGNYLECGVNQEFCESLQQEFTFS
NseV               TNIHYFQRYELLMYDNLETGVLQYNNYSLDRHIYQNDNCYKH--TDKYCVYDPELFETIIVYNKYGNHLECGVNHEFCISLQKEFVYV
MenoV              TKLRYFQQYELRMYDDLMSGVLQYNNYKLERHMYKNSYCLSTDTKTNHCIYDPTLFNTTVVKNANGNYLECGVNHKFCEDFQLEFAAV
                    :: ***.:** :**::  ***:****.*  .:*.*  *          * *:    *    * *  **.****::::** .:* **

HouV (16757)       EPQLVITESLAVEVPTQYHKICDNHYTALQVKYPLIEKLFWSNFNVSVNRALAVK---EPATFIIVHDTVAIIKTIIADIIEIMEECY
HouV (V3872)       EPQLVITESLAVEVPTQYHKICDNHYTALQVKYPLIEKLFWSNFNVSVNRALAVK---EPATFIIVHDTVAIIKTIIADIIEIMEECY
HouV (16740)       EPQLVITESLAVEVPTQYHKICDNHYTALQVKYPLIEKLFWSNFNVSVNRALAVK---EPATFIIVHDTVAIIKTIIADIIEIMEECY
HouV (V3982)       EPQLVITESLAVEVPTQYHKICDNHYTALQVKYPLIEKLFWSNFNVSVNRALAVK---EPATFIIVHDTVAIIKTIIADIIEIMEECY
NDiV               EPQLVITESMAVEAPTQYHKICDNHYTSLQVKYPLIEKLFWSNFXVSVNRALAVK---EPATFIIVHDTVAIIKTVIADIIEIMEKCY
NgeV (JKT9982)     EPQLVITESLAVEVPTQYHKICDNHYTSLQVKYPLIEKLFWSNFYVSVNRALAVK---EPATFIIVHDTVAIIKTVIADIIEIMEACY
CavV               EPQLVITESSTAEVSAQYKKICANHYTTLQSKYPLIEKLFWSNFNVSVNRALAAK---EPATFLIVYDTPAIVKTIIADIIETMEECY
HanaV              EPTLVISEIPIAEEPIMYHKICDNHYTALKSKYPLIAENFWRMYNVSMKHALSETSTEPLATIVIVHDTETIVKTVIADLIHVVEECY
KPhV (KP84_0156)   EPELVITSNPIIETPTHYFKICDNHYTALQAKYPMVEKSFWGSFIVNAKRAFAGK---EPATFIIVHDSATVIKTVIADVLAVAEECY
KPhV (KP84_0192)   EPELVITSNPIIETPTHYFKICDNHYTALQAKYPMVEKSFWGSFIVNAKRAFAGK---EPATFIIVHDSATVIKTVIADVLAVAEECY
KphV (KP84_0344)   EPELVITSNPIIETPTHYFKICDNHYTALQAKYPMVEKSFWGSFIVNAKRAFAGK---EPATFIIVHDSATVIKTVIADVLAVAEECY
BBaV (JKT9876)     EPDLVITENPSVEDPTSYYEICSNHYTALQTKYPLIEKSFWAHFAVSIKYALAGK---EPATFIIVHDSETVVKSVIADMIAIAEECY
BBaV (JKT9891)     EPDLVITENPSVEDPTSYYEICSNHYTALQTKYPLIEKSFWAHFAVSIKYALAGK---EPATFIIVHDSETVVKSVIADMIAIAEECY
BBaV (JKT9853)     EPDLVITENPSVEDPTSYYEICSNHYTALQTKYPLIEKSFWAHFAVSIKYALAGK---EPATFIIVHDSETVVKSVIADMIAIAEECY
BBaV (JKT7774)     EPDLVITENPSVEDPTSYYEICSNHYTALQTKYPLIEKSFWAHFAVSIKYALAGK---EPATFIIVHDSETVVKSVIADMIAIAEECY
KSaV (JKT10701)    ESDLVITENPSVEAPIRYYEICNNHYTALQTKYPLIEKEFWTNFAISIKNALVGK---EPATFIIVHDSETVVKSVIADMIAIAEECY
NseV               EEIDTVEIK-----QVNNEIQCALPLDTLRTKYPKIDDKVFVQFNVGIKNAIQNK---SPSTTTFLYDSDLVVKSLVEDLIVATENCF
MenoV              EYIPEIQPKTTDVCEPLFNYLKSSDLTSDAISIIQIITQATKEKLPANQITFALN-----GTLRDLQPLATYMQNCVDANNIVKTTSG
                   *     :                       :    .    :              : ::  .      .*    :       ::. :         .

HouV (16757)       DSTAIKLTHHDFNKLEYMDDYSQILVKYKPLLEQHKIMLIEDIDLITVPAARALFSIFDTYTPLVNGVFVLGTLNHKRYNETLSFNY-
HouV (V3872)       DSTAIKLTHHDFNKLEYMDDYSQILVKYKPLLEQHKIMLIEDIDLITVPAARALFSIFDTYTPLVNGVFVLGTLNHKRYNETLAFNY-
HouV (16740)       DSTAIKLTHHDFNKLEYMDDYSQILVKYKPLLEQHKIMLIEDIDLITVPAARALFSIFDTYTPLVNGVFVLGTLNHKRYNETLSFNY-
HouV (V3982)       DSTAIKLTHHDFNKLEYMDDYSQILVKYKPLLEQHKIMLIEDIDLITVPAARALFSIFDTYTPLVNGVFVLGTLNHKRYNETLAFNY-
NDiV               DSTAIKLTHHDFNKLEYMDDYSQILVKYKPLLEQHKIMLIEDIDLITVPAARALFSIFDTYTPLVNGVFVLGTLNHKRYNETLAFNY-
NgeV (JKT9982)     DSTAIKLTHHDFNKLEYMDDYSQILVKYKPLLEQHKIMLIEDIDLITVPAARALFSIFDTYTPLVNGVFVLGTLNHKRYNETLAFNY-
CavV               GNTAIKLTSNDFNKLD---DYNDFLIEHKPLLAKHKIMLIEDIDLIKVSMARAIYTIFDTYNPLVYGVFVLGTLNHKRYNETLAFNY-
HanaV              DKTALKLTYLDFNNVE---DYDQILVKITPLLQQHKILVIEDIDLIKASSARALFSIFDTYEPLVKGAFIIATLNHKRYNETLAFND-
KPhV (KP84_0156)   VRTALKLKPQDFNFTS---DYSEILEAYHPLIKKQKIVLIEDIDQLTVPAARSLFSFFDTYTPAVKDAFIISTLNHKRYTESVSLNL-
KPhV (KP84_0192)   VRTALKLKPQDFNFTS---DYSEILEAYHPLIKKQKIVLIEDIDQLTVPAARSLFSFFDTYTPAVKDAFIISTLNHKRYTESVSLNL-
KphV (KP84_0344)   VRTALKLKPQDFNFTS---DYSEILEAYHPLIKKQKIVLIEDIDQLTVPAARSLFSFFDTYTPAVKDAFIISTLNHKRYTESVSLNL-
BBaV (JKT9876)     EKTALRLTHKDFNAIP---DYSDIIEKYYPLISEQKIVLIEDIDLITVPAIRSLYSFFDTYTPAVQDAFILGTFNHKHYTETLALNR-
```

```
BBaV (JKT9891)          EKTALRLTHKDFNAIP---DYSDIIEKYYPLISEQKIVLIEDIDLITVPAIRSLYSFFDTYTPAVQDAFILGTFNHKHYTETLALNR-
BBaV (JKT9853)          EKTALRLTHKDFNAIP---DYSDIIEKYYPLISEQKIVLIEDIDLITVPAIRSLYSFFDTYTPAVQDAFILGTFNHKHYTETLALNR-
BBaV (JKT7774)          EKTALRLTHKDFNAIP---DYSDIIEKYYPLISEQKIVLIEDIDLITVPAIRSLYSFFDTYTPAVQDAFILGTFNHKHYTETLALNR-
KSaV (JKT10701)         ESTALKLTHKDFNVIP---DYSDIIAKYHPLISKQKIVLIEDIDLITVPAIRSLFSYFDTYTPAVKDAFILCTLNHKRYTEVLALNL-
NseV                    DRLALRLNYTYFNQPQYLEDYRALINDLKPQLEKHSVLVIDDIDKISPQTIQSLFQICDAYEPLVPRAVIIATINTEQAFEIVHTDEN
MenoV                   SELDLYT------------DRNSLMEHLQTIYSNTNIILVNDVQDLDCNKATAFFSFIDPYIDIPTYPHTLTLFTQQVK---------
                            :              *   ::    .   : .:::::*:: :      :::   *.*         :  :. :
HouV (16757)            YNDQTPTSY----VDGILNANWQLLEDHTRQPLITRVADNVHTILARQL--VTPPAKIAATMPKLPTLNNTSKVINTLTHYSAHLLQ
HouV (V3872)            YNDQTPTSY----VDGILNANWQLLEDHTRQPLITRVADNVHTILARQL--VTPPAKIAATMPKLPTLNNTSKVINTLTHYSAHLLQ
HouV (16740)            YNDQTPTSY----VDGILNANWQLLEDHTRQPLITRVADNVHTILARQL--VTPPAKIAATMPKLPTLNNTSKVINTLTHYSAHLLQ
HouV (V3982)            YNDQTPTSY----VDGILNANWQLLEDHTRQPLITRVADNVHTILARQL--VTPPAKIAATMPKLPTLNNTSKVINTLTHYSAHLLQ
NDiV                    YNDQTPTFY----VDGILNANWQLLEDHTRQPLITRVADNVHTILARQL--VTPPAKIAATMPKVPTLNNTSKVINTLTHYSAHLLQ
NgeV (JKT9982)          YNDQTPTFY----VDGILNANWQLLEDHTRQPLITRVADNVHTILARQL--VTPPAKIAATMPKVPTLNNTSKVINTLTHYSAHLLQ
CavV                    YNGQTPTSY----VEGILTNNWRELEDHTRQPLIIRVTDNVHTILARQL--VTPPAKLAETMSKIPPLNNTSKVINTLTHYSAHILR
HanaV                   YNPTTPASY----VDDILHKNWQELPNHTRLPLITRVGDNVHTIFGKQL--VTPPEKLIPEMPKMPALNNTSTVINTLTHYSAHILQ
KPhV (KP84_0156)        YNDQTPTSY----VEDILRENWKSMAVDTLTPLIVRMTDNVHIILANQV--VTP-GKLSTPESKIPTLNNTSKVINTFTHYSAHILR
KPhV (KP84_0192)        YNDQTPTSY----VEDILRENWKSMAVDTLTPLIVRMTDNVHIILANQV--VTP-GKLSTPESKIPTLNNTSKVINTFTHYSAHILR
KphV (KP84_0344)        YNDQTPTSY----VEDILRENWKSMAVDTLTPLIVRMTDNVHIILANQV--VTP-GKLSTPESKIPTLNNTSKVINTFTHYSAHILR
BBaV (JKT9876)          YNDITPTSY----VESILEENWKLLDNYTLTPLIVRITDNVHTILAYQV--ATP-AKLANPMPEKPTLNNTSKVINTFTHYSAHILR
BBaV (JKT9891)          YNDITPTSY----VESILEENWKLLDNYTLTPLIVRITDNVHTILAYQV--ATP-AKLANPMPEKPTLNNTSKVINTFTHYSAHILR
BBaV (JKT9853)          YNDITPTSY----VESILEENWKLLDNYTLTPLIVRITDNVHTILAYQV--ATP-AKLANPMPEKPTLNNTSKVINTFTHYSAHILR
BBaV (JKT7774)          YNDITPTSY----VESILEENWKLLDNYTLTPLIVRITDNVHTILAYQV--ATP-AKLANPMPEKPTLNNTSKVINTFTHYSAHILR
KSaV (JKT10701)         YNDQTPTSY----VEGIFKENWKLLDDKTLIPLIVRVTDNVQTILAYQV--ATPPAKLSQPMPEVPTLNNTSKVINTFTHYSAHILR
NseV                    IFNMDPVAYGLKDVEDLYTKRWGSLDQNMLIPLIVRLTENVFTITLEHLTGIKPPTPVVVVPTVTPTENIIYKTANTYTHYSAYLLE
MenoV                   LFNVSSLQY----LTDYYQN--CIVTNEWNTPFAVRMTENIITISTVFD-------DSNEIPIETPLESQILNTLNYYTTYFSQVFY
                             .  *    : .        :      *:  *: :*:  *                     *  .   .. *  * * : ::

HouV (16757)            AVGSDSTQAYTYINSSVHNITDYVNDSVHNITDYVYTVYNSTKNHIVTRYNNMLMAAYDIQLNFYNQYPLRQDFYHKGIRAFDLGQV
HouV (V3872)            AVGSDSTQAYTYINSSVHNITDYVNDSVHNITDYVYTVYNSTKNHIVTRYNNMLMAAYDIQLNFYNQYPLRQDFYHKGIRAFDLGQV
HouV (16740)            AVGSDSTQAYTYINSSVHNITDYVNDSVHNITDYVYTVYNSTKNHIVTRYNNMLMAVYDIQLNFYNQYPLRQDFYHKGIRAFDLGQV
HouV (V3982)            AVGSDSTQAYTYINSSVHNITDYVNDSVHNITDYVYTVYNSTKNHIVTRYNNMLMAAYDIQLNFYNQYPLRQDFYHKGIRAFDLGQV
NDiV                    VVGSDSTQAYTYINSSVHNITDYVNDSVHNITDYVYTVYNSTKNHIVTRYNNMLMAAYDIQLNFYNQYPLRQDFYHKGIRAFDLGQV
NgeV (JKT9982)          AVGSDSTQAYTYINSSVHNITDYVNDSVHNITDYVYTVYNSTKNHIVTRYNNMLMAAYDIQLNFYNQYPLRQDFYHKGIRAFDLGQV
CavV                    TVENDSTQAYTYINKSVHNITDYVNGTVHNFTDYVYTAYNTTKNHIVTRYNNMLMAVYDIQLNFYNRYPLRQDFYHKGMRAFDLGRV
HanaV                   SVGNDSTLAYNYINNSVYNITESVNDIIYNFTDYVKMAYNTTKYHIFTRYNNMLMAIYDIQQNYYNQYPLRQDFYYKSSRAFDLGQV
KPhV (KP84_0156)        TVGDDSTQAYKYLNNSVNNFTEYVSGSAYSFYDYIHTALKTTKYQIFKRYSNMLSAYNDIKRNLYNSYPIRQDFYRRSAHLFDSGRV
KPhV (KP84_0192)        TVGDDSTQAYKYLNNSVNNFTEYVSGSAYSFYDYIHTALKTTKYQIFKRYSNMLSAYNDIKRNLYNSYPIRQDFYRRSAHLFDSGRV
KphV (KP84_0344)        TVGDDSTQAYKYLNNSVNNFTEYVSGSAYSFYDYIHTALKTTKYQIFKRYSNMLSAYNDIKRNLYNSYPIRQDFYRRSAHLFDSGRV
BBaV (JKT9876)          TVGDDSTQAYNYLNSSVNNITDYVSESAHDFYSYVNTAFKLTRRQILKRYRNMLSAYYDIQRNFYNSYPIRQDFYNRHAHLFDSGRI
BBaV (JKT9891)          TVGDDSTQAYNYLNSSVNNITDYVSESAHDFYSYVNTAFKLTRRQILKRYRNMLSAYYDIQRNFYNSYPIRQDFYNRHAHLFDSGRI
BBaV (JKT9853)          TVGDDSTQAYNYLNSSVNNITDYVSESAHDFYSYVNTAFKLTRRQILKRYRNMLSAYYDIQRNFYNSYPIRQDFYNRHAHLFDSGRI
BBaV (JKT7774)          TVGDDSTQAYNYLNSSVNNITDYVSESAHDFYSYVNTAFKLTRRQILKRYRNMLSAYYDIQRNFYNSYPIRQDFYNRHAHLFDSGRI
KSaV (JKT10701)         TVGDDSTQAYNYLNNSVNNITDYVSESAHDFYDYVNDALKLTKRHILKRYRNMLMAYYDIQRNFHNSYPIRQDFYQRSVYLFDSGRI
NseV                    VFNRERHYAYDYLADTVEVFNNVS-----------RVVCDTVYDSVHLRYNNVRKAYNDIKRNFANQYPQRRDFYRHGQRAFDAGQV
MenoV                   TKQTTYTDLFT-------------------------ATYETLKQQLTTRVHNVVDSFTTMTEKFEQHYPLRNDFYHQTLRAFDLGRF
                                 :                           . .     :  *  *:  :   :  :  : ** *.*** :    ** *:.

HouV (16757)            CDFLHHTDTIVLYQDCINQKLDTIYVIKLRYGQNANGYHMYPLKQPHTKQTIYELSDAIGFVYKDSKYNYFRTLFTNPGEYVLTIRE
HouV (V3872)            CDFLHHTDTIVLYQDCINQKLDTIYVIKLRYGQNANGYHMYPLKQPHTKQTIYELSDAIGFVYKDSKYNYFRTLFTNPGEYVLTIRE
HouV (16740)            CDFLHHTDTIVLYQDCINQKLDTIYVIKLRYGQNANGYHMYPLKQPHTKQTIYELSDAIGFVYKDSKYNYFRTLFTNPGEYVLTIRE
HouV (V3982)            CDFLHHTDTIVLYQDCINQKLDTIYVIKLRYGQNANGYHMYPLKQPHTKQTIYELSDAIGFVYKDSKYNYFRTLFTNPGEYVLTIRE
NDiV                    CDFLHHTDTIVLYQDCINQKLDTIYVIKLRYGQNANGYHMYPLKQPHTKQTIYELSDAIGFVYKDSKYNYFRTLFTNPGEYVLTIRE
NgeV (JKT9982)          CDFLHHTDTIVLYQDCINQKLDTIYVIKLRYGQNANGYHMYPLKQPHTKQTIYELSDAIGFVYKDSKYNYFRTLFTNPGEYVLTIRE
CavV                    CDFLHHSDTIVLYQDCINQKLDTIHVIKLRYGQNANAYHMYPLELPHTKQTIYELSDAIGFVYKDRKYNYFRTLFTNPGEYVLTIRE
HanaV                   CDFLHHSDTIIMYQDCVKQNLDEVYVIKLRYGQNANAYHMYPLKQPYTKQTIYELSDAIGFVYKDRKYNYFRPLFTNPGEYVLTIRE
KPhV (KP84_0156)        CDYLHHSDTMVLYQDCIHEKLDTIYVIKLRYGQNANAFHIQTLEQPHTKQYISALSEAIGFEYQGKKYNYYRMLFTNPDEYVLTIRE
KPhV (KP84_0192)        CDYLHHSDTMVLYQDCIHEKLDTIYVIKLRYGQNANAFHIQTLEQPHTKQYISALSEAIGFEYQGKKYNYYRMLFTNPDEYVLTIRE
KphV (KP84_0344)        CDYLHHSDTMVLYQDCIHEKLDTIYVIKLRYGQNANAFHIQTLEQPHTKQYISALSEAIGFEYQGKKYNYYRMLFTNPDEYVLTIRE
BBaV (JKT9876)          CDYLHHSDTIVLYQDCIREKLDTIYVIKLRYGQPANAFHVQPLEPPHTKQYILALSEAIGFEYQGKKHNYYRTLFTNPGEYALTIRE
BBaV (JKT9891)          CDYLHHSDTIVLYQDCIREKLDTIYVIKLRYGQPANAFHVQPLEPPHTKQYILALSEAIGFEYQGKKHNYYRTLFTNPGEYALTIRE
BBaV (JKT9853)          CDYLHHSDTIVLYQDCIREKLDTIYVIKLRYGQPANAFHVQPLEPPHTKQYILALSEAIGFEYQGKKHNYYRTLFTNPGEYALTIRE
BBaV (JKT7774)          CDYLHHSDTIVLYQDCIREKLDTIYVIKLRYGQPANAFHVQPLEPPHTKQYILALSEAIGFEYQGKKHNYYRTLFTNPGEYALTIRE
KSaV (JKT10701)         CDYLHHSDTIVLYQDCIREKLDTIYVIKLRYGQPANAFHVQPLEPPHSKQHISALSEAIGFEYQGHKYNYYRTLFTNPDEYALTIRE
NseV                    CEFLHHTDTIVLYQDCVQDKLEDIYVMKLRYGVDANEYYTHKLETPFTKQTYHDLTEATGFIYKDYNYIYFRPLFTNPGEHVLTIRE
MenoV                   CDFLHHTDTLSLYNDCVSDQLQPISVINLRYGLDSNAYSTHDIDLPINKQSLYSLTQATGYIYRGQKHLFYRPLFTNANQYVLTVRE
                        *::***:**: :*:**: ::*: : *::****  :* :    :. * .**    *::* *: *:. :: ::* ****..::.**:**

HouV (16757)            NYLEYCKSDISPTPAFAPDATLQCYAYITGIQVID--NFIAEFGLFLMLYTAALIIILALAITIRDSTMMMFLKLSIIFAYTFGPLLL
HouV (V3872)            NYLEYCKSDISPTPAFAPDATLQCYAYITGIQVID--NFIAEFGLFLMLYTAALIIILALAITIRDSTMMMFLKLSIIFAYTFGPLLL
HouV (16740)            NYLEYCKSDISPTPAFAPDATLQCYAYITGIQVID--NFIAEFGLFLMLYTAALIIILALAITIRDSTMMMFLKLSIIFAYTFGPLLL
HouV (V3982)            NYLEYCKSDISPTPAFAPDATLQCYAYITGIQVID--NFIAEFGLFLMLYTAALIIILALAITIRDSTMMMFIKLSIIFAYTFGPLLL
NDiV                    NYLEYCKSDFSPTPAFAPDATLQCYAYITGIQVID--NFIAEFGLFLMLYTAALIIILALAITIRDSTMMMFLKLSIIFAYTFGPLLL
NgeV (JKT9982)          NYLEYCKSDFSPTPAFAPDATLQCYAYITGIQVID--NFIAEFGLFLMLYTAALIIILALAITIRDSTMMMFLKLSIIFAYTFGPLLL
CavV                    NYLEYCKSDNSPTPAFAPDVPLQCFAYITGVQVVD--SFIAEFGLFLMLYIAALIIILAIAITIRDNTMMMFLKLITIFAYTFGHLLL
HanaV                   DYLEYCKSDTSPTPAFAPDANLQCYAYITGVQVID--NFIAEFGIFLMLYTAALIIILAIAVTIRDSTMVMILKQVIIFAYAFGPWLL
KPhV (KP84_0156)        NYLEYCKSSISPTPAFAPGTKLTCFAYITGFKVIDRDSFIAEFGLFLMLYTAALIIILAVAITIRDNNYIMLVKQSICFAYIFGPLLL
KPhV (KP84_0192)        NYLEYCKSSISPTPAFAPGTKLTCFAYITGFKVID--SFIAEFGLFLMLYTAALIIILAVAITIRDNNYIMLVKQSICFAYIFGPLLL
KphV (KP84_0344)        NYLEYCKSSISPTPAFAPGTKLTCFAYITGFKVID--SFIAEFGLFLMLYTAALIIILAVAITIRDNNYIMLVKQSICFAYIFGPLLL
BBaV (JKT9876)          NYLEYCKSDISPTPAFTPNATLQCFAYITGFRVID--SFIAEFGLFLMLYIAALIIILAVAITIRDNTVIMLLKQFLIFVYIFGPWLL
```

```
BBaV (JKT9891)      NYLEYCKSDISPTPAFTPNATLQCFAYITGFRVID--SFIAEFGLFLMLYIAALIIILAVAITIRDNTVIMLLKQFLIFVYIFGPWLL
BBaV (JKT9853)      NYLEYCKSDISPTPAFTPNATLQCFAYITGFRVID--SFIAEFGLFLMLYIAALIIILAVAITIRDNTVIMLLKQFLIFVYIFGPWLL
BBaV (JKT7774)      NYLEYCKSDISPTPAFTPNATLQCFAYITGFRVID--SFIAEFGLFLMLYIAALIIILAVAITIRDNTVIMLLKQFLIFVYIFGPWLL
KSaV (JKT10701)     NYLEYCKSEISPTPAFTPNATLQCFAYITGFKVID--SFIAEFGLFLVLYIAALIIILAVAITIRDNTGIMLLKQFLIFAYIFGPWLL
NseV                DYLEYCKSDTSPTPAFATEAPLSCYAYITGVQFLD--HFISEFGWFFILYTAALLIILVISVAIREYTTTMIVKMTLIFLYSFGPWLL
MenoV               DYLEYCKTSTIPVPAFTPETTLTCFPYITGAQSID--HFIAEIGPFLILYTAALTIIVLAAVQIRENKYIMFIKMCIIFVYAFGPRIL
                    :******:.  *.***:. . * *:.**** : :*   **:*:* *::** *** **:  :: **: .  *::*    * * **  :*
HouV (16757)        TPKVFGSYIFVSFYNMLPYTSNTSYGCLLMMGALAITVIDLFSYMT---QRYRSEFTKNILQLATLLFEIVAITKYILIPYICTSYGL
HouV (V3872)        TPKVFGSYIFVSFYNMLPYTSNTSYGCLLMMGALAITVIDLFSYMT---QRYRSEFTKNILQLATLLFEIVAITKYILIPYICTSYGL
HouV (16740)        TPKVFGSYIFVSFYNMLPYTSNTSYGCLLMMGALAITVIDLFSYMT---QRYRSEFTKNILQLATLLFEIVAITKYILIPYICTSYGL
HouV (V3982)        TPKVFGSYIFVSFYNMLPYTSNTSYGCLLMMGALAITVIDLFAYMT---QRYRSEFTKNILQLATLLFEIVATTKYILIPYICTSYGL
NDiV                TPKVFGSYIFVSLYNMLPYTSNTSYGCLLMMGALAITVIDLFAYMT---QRYRSEFTKNILQLATLLFEIVAITKYILIPYVCTSYGL
NgeV (JKT9982)      TPKVFGSYIFVSFYNMLPYTSNTSYGCLLMMGALAITAIDLFAYMT---QRYRSEFTKNILQLVTLLFEIVAITKYILIPYICTSYGL
CavV                TPRVYGSYMFLSIYNILPYTSNTSYGCLLMLGALAIAVIDLLAYIT---QRYRSEFTKNVLQLVTLFFEIAAVTKYILIPYIFTSYGL
HanaV               TPNVFGSYIFVKIYNLIPYTSNTSYGCLLMVGTLAITTIDLFAYLT---QRYRAEFTKHVLQLVTLVFELVAVSKYILLPYILTSYGF
KPhV (KP84_0156)    TPNVYGSYIFVTLYNMLPYTGGTSYGCLLAMATIAITIVDFLAYTT---QRYRPEFTKHVLQVTTILFEIVVVSRYILFPYIFTSYGF
KPhV (KP84_0192)    TPNVYGSYIFVTLYNMLPYTGGTSYGCLLAMATIAITIVDFLAYTT---QRYRPEFTKHVLQVTTILFEIVVVSRYILFPYIFTSYGF
KphV (KP84_0344)    TPNVYGSYIFVTLYNMLPYTGGTSYGCLLAMATIAITIVDFLAYTT---QRYRPEFTKHVLQVTTILFEIVVVSRYILFPYIFTSYGF
BBaV (JKT9876)      TPKVYGSYIFVKLYNLIPYTSSTSYGCLLAMVTIAITIIDVLAYTT---QRYYPELTKHFLQIVTISFEIVAVTRYILLPYIFTSYGF
BBaV (JKT9891)      TPKVYGSYIFVKLYNLIPYTSSTSYGCLLAMVTIAITIIDVLAYTT---QRYYPELTKHFLQIVTISFEIVAVTRYILLPYIFTSYGF
BBaV (JKT9853)      TPKVYGSYIFVKLYNLIPYTSSTSYGCLLAMVTIAITIIDVLAYTT---QRYYPELTKHFLQIVTISFEIVAVTRYILLPYIFTSYGF
BBaV (JKT7774)      TPKVYGSYIFVKLYNLIPYTSSTSYGCLLAMVTIAITIIDVLAYTT---QRYYPELTKHFLQIVTISFEIVAVTRYILLPYIFTSYGF
KSaV (JKT10701)     TPKVYGSYILVKLYNLIPYTSSTSYGCLLAMATVAITIIDVIAYMT---QRYRPELTKHFLQVVTMSFEAVAITRFILLPYIFTSYGF
NseV                TPKFYGSYILVKLYNSLPYLIGNSYGCLLVLSVLLITTIDLMSYTT---QRFKPEFSRHVLQISTLAFEVMAVLYYIFIPYLFTSYGF
MenoV               TPAFPGSFILSWLYNHIPFVASTSYGVLLMLSILLTVTIDFVSFVTTYSKRTPEQLRTCVLHFVALVFEVLATFYYILLPYLFTGYGI
                    ** . **:::  :** :*:  ..*** ** :  :  . :*..:: *   :*   ::   .*:. :: **  .   :*::**: *.**:

HouV (16757)        VLTIIVSYVAYRYIQSQRPNYLKATVSNATAHADWVAYRNTTREKTDEAAKSNLSKIINTSIADINKDQFLECVYLAACHRATVAAST
HouV (V3872)        VLTIIVSYVAYRYIQSQRPNYLKATVSNATAHADWVAYRNTTREKTDEAAKSNLSKIINTSIADINKDQFLECVYLAACHRATVAAST
HouV (16740)        VLTIIVSYVAYRYIQSQRPNYLKATVSNATAHADWVAYRNTTREKTDEAAKSNLSKIINTSIADINKDQFLECVYLAACHRATVAAST
HouV (V3982)        VLTIIVSYVAYRYIQSRRPNYLKATVSNATAHADWVAYRNTTREKTDEAAKSNLSKIINTSIADINKDQFLECVYLAACHRATVAAST
NDiV                VLTIIVSYVAYRYIQSQRPNYLKATVSNATAHADWVAYRNTTREKTDEAAKSNLSKIINTSIADINKDQFLECVYLAACHRATVAAST
NgeV (JKT9982)      VLTIIVSYVAYRYIQSQRPNYLKATVSNATAHADWVAYRNTTREKTDEAAKSNLSKIINTSIADINKDQFLECVYLAACHRATVAAST
CavV                VLTIIVSYVAYRYIHSRRPNYLKATVSNATAHADWVAYRNTTREKTDEAAKSNLSKIINTSVAEIQKDQLLECLYLAACHRATVASST
HanaV               VLLTIVSYVAYLYTRSQRPNYLKSSVSNAVAHADWVAYRNSTREKTDEAAKSNLSKIINTNVADIKKEQLLECLYLAACHQATVAAST
KPhV (KP84_0156)    VLTIIASYVAYLIIQARRPNYLKSTVSNATAHADWVAYRNSTREKTDEAAKSNLSKIINTNVADIKKEQLLECLYLAACHQATVAAQT
KPhV (KP84_0192)    VLTIIASYVAYLIIQARRPNYLKSTVSNATAHADWVAYRNSTREKTDEAAKSNLSKIINTNVADIKKEQLLECLYLAACHQATVAAQT
KphV (KP84_0344)    VLTIIASYVAYLIIQARRPNYLKSTVSNATAHADWVAYRNSTREKTDEAAKSNLSKIINTNVADIKKEQLLECLYLAACHQATVAAQT
BBaV (JKT9876)      VLTIIASYVAYRFIQARRPNYLKSSVSNATAHADWVAYRNSTREKTDEAAKSNLSKIINTNVADIKKEKLLECVYLAACHQATVAAQT
BBaV (JKT9891)      VLTIIASYVAYRFIQARRPNYLKSSVSNATAHADWVAYRNSTREKTDEAAKSNLSKIINTNVADIKKEKLLECVYLAACHQATVAAQT
BBaV (JKT9853)      VLTIIASYVAYRFIQARRPNYLKSSVSNATAHADWVAYRNSTREKTDEAAKSNLSKIINTNVADIKKEKLLECVYLAACHQATVAAQT
BBaV (JKT7774)      VLTIIASYVAYRFIQARRPNYLKASVSNATAHADWVAYRNSTREKTDEAAKSNLSKIINTNVADIKKEKLLECVYLAACHQATVAAQT
KSaV (JKT10701)     VLTIIASYVAYRFVQARRPNYLKSSVSNATAHADWVAYRNTTREKTDEAAKSNLSKIINTNVADIKKEQLLECLYLAACHQATVAAQT
NseV                VITILVSYVAYIHYLSQRPNYKRSYVTNEVAHADWVRYRNSTREKTDEAAKSNLRKIINTNVADLNKESLLECIYLATCYRATVQDSP
MenoV               LMLIITTYVAHIYRRSQLPNYRRSSVTMATAHADWVKYRESTREKTDEAAKSSLRRVMNMYIGDATSDKLLESVYLAACYTNTVLAPT
                    ::  :.:***:    :: *** :: *:  .****** **::***********.* :::*  :.:  .:.:**.:***:*:  **   .

HouV (16757)        YNPKHYLHIPNYNTKIMFARDNELMNYS----VLSTDLKNKSAASNPSISHIVLEMPVAINPLIKYTTKTSVSSLRGAVVNGYIYIQR
HouV (V3872)        YNPKHYLHIPNYNTKIMFARDNELMNYS----VLSTDLKNKSAASNPSISHIVLEMPVAINPLIKYTTKTSVSSLRGAVVNGYIYIQR
HouV (16740)        YNPKHYLHIPNYNTKIMFARDNELMNYS----VLSTDLKNKSAASNPSISHIVLELPVAINPLIKYTTKTSVSSLRGAVVNGYIYIQR
HouV (V3982)        YNPKHYLHIPNYNTKIMFARDNELMNYS----VLSTDLKNKSAASNPSISHIVLEMPVAINPLIKYTTKTSVSSLRGAVVNGYIYIQR
NDiV                YNPKHYLHIPNYNTKIMFARDNELMNYS----VLSTDLKNKSAASNPSISHIVLELPVAINPLIKYTTKTSVSSLRGAVVNGYIYIQR
NgeV (JKT9982)      YNPKHYLHIPNYNTKIMFARDNELMNYS----VLSTDLKNKSAASNPSISHIVLEMPVAINPLIKYTTKTSVSSLRGAVVNGYIYIQR
CavV                YNPKHYLHIPNYNTKIMFARDNELMNYS----VLSTDFKNKSAASNPSISHIVLEMPVAINPLIKYTTRTSVSSLRGAVVNGYIYIQR
HanaV               YNPKHYLHIPNYNTKIMFARDNELMNYS----VLTTDLKNKSAASNPSIAHNVLELPVTINPLIKYTTRTSVSSLRGAVVNGYIYIQR
KPhV (KP84_0156)    YNPKHYLHIPNYNTRIMFARDNELMNYSS---VLSTDLKNKSAASNPSISHIVLELPVTINPLIKYTTKTSVSSLRGAIVNGYIYIQR
KPhV (KP84_0192)    YNPKHYLHIPNYNTRIMFARDNELMNYSS---VLSTDLKNKSAASNPSISHIVLELPVTINPLIKYTTKTSVSSLRGAIVNGYIYIQR
KphV (KP84_0344)    YNPKHYLHIPNYNTRIMFARDNELMNYSS---VLSTDLKNKSAASNPSISHIVLELPVTINPLIKYTTKTSVSSLRGAIVNGYIYIQR
BBaV (JKT9876)      YNPKHYLHIPNYNTRIMFARDNELMNYSA---VQPTNLKNKSAASNPSISHIVLELPVTINPLIKYTTKTSVSSLRGAIVNGYIYVQR
BBaV (JKT9891)      YNPKHYLHIPNYNTRIMFARDNELMNYSA---VQPTNLKNKSAASNPSISHIVLELPVTINPLIKYTTKTSVSSLRGAIVNGYIYVQR
BBaV (JKT9853)      YNPKHYLHIPNYNTRIMFARDNELMNYSA---VQPTNLKNKSAASNPSISHIVLELPVTINPLIKYTTKTSVSSLRGAIVNGYIYVQR
BBaV (JKT7774)      YNPKHYLHIPNYNTRIMFARDNELMNYSA---VQPTNLKNKSAASNPSISHIVLELPVTINPLIKYTTKTSVSSLRGAIVNGYIYVQR
KSaV (JKT10701)     YNPKHYLHIPNYNTRIMFARDNELMNYSA---VQPTKLKNKSAASNPSISHIVLELPVAINPLIKYTTKTSVSSLRGAIVNGYIYIQR
NseV                FNPRHQLHIPSYNASVMFSRDNEIMMQSL---MTSTDFKNKSAASNPSIPHKVLGLPVTINPLITYMSNKYASSLRGSVVNGYIYIQR
MenoV               FNPRHTLHIPRCSNSVFCSTASEVMNHSSNDSNMSTTIRNKSALSNPSIPYIALPLPVSINPLITYDS---VSSLRGSVVNGYIYILR
                    :**:* ****   .  :: :  .*:*  *      .* ::**** *****.: .* :**:*****.* :   .*****::******: *

HouV (16757)        HLFGSKKQEFEACYNNGKGLLNCKNLDRSKYDIDSAELIGTLIRIPLHDKQSIPHISLHPDPLSYNGPVTLYLSRYDTELNKDVLCVH
HouV (V3872)        HLFGSKKQEFEACYNNGKGLLNCKNLDRSKYDIDSAELIGTLIRIPLHDKQSIPHISLHPDPLSYNGPVTLYLSRYDTELNKDVLCVH
HouV (16740)        HLFGSKKQEFEACYNNGKGLLNCKNLDRSKYDIDSAELIGTLIRIPLHDKQSIPHISLHPDPLSYNGPVTLYLSRYDTELNKDVLCVH
HouV (V3982)        HLFGSKKQEFEACYNNGKGLLNCKNLDRSKYDIDSAELIGTLIRIPLHDKQSIPHISLHPDPLSYNGPVTLYLSRYDTELNKDVLCVH
NDiV                HLFGSKKQEFEACYNNGKGLLNCKNLDRSKYDIDSAELIGTLIRIPLHDKQSIPHISLHPDPLSYNGPVTLYLSRYDTELNKDVLCVH
NgeV (JKT9982)      HLFGSKKQEFEACYNNGKGLLNCKNLDRSKYDIDSAELIGTLIRIPLHDKQSIPHISLHPDPLSYNGPVTLYLSRYDTELNKDVLCVH
CavV                HLFGSKKQEFEACYNNGKGLLNCKNLERSKYDIDSAELIGTLIRIPLHDKHSIPHISIHPDPLSYNGPVTLYLSRYDTELNKDVLCVH
HanaV               HLFGNKKQVFEACYNNGKGLLNCKNLDRSKYDIDSAELIGTLIRIPLVDKSSVPNINIHPAPLTYNGPVTLYLSRYDTELNKDVLCVH
KPhV (KP84_0156)    HLFGSKRQEFEACYNNGKGLLNCHNLERSKYDIDSAELIGTLIRIPLLNDHSFPKIKLHPAPLTYNGPVTLYLSRYDTELQKDVLCVH
KPhV (KP84_0192)    HLFGSKRQEFEACYNNGKGLLNCHNLERSKYDIDSAELIGTLIRIPLLNDHSFPKIKLHPAPLTYNGPVTLYLSRYDTELQKDVLCVH
KphV (KP84_0344)    HLFGSKRQEFEACYNNGKGLLNCHNLERSKYDIDSAELIGTLIRIPLLNDHSFPKIKLHPAPLTYNGPVTLYLSRYDTELQKDVLCVH
BBaV (JKT9876)      HLFGSKKQEFEACYNNGKGLLNCHNLERSKYDIDSAELIGTLIRIPLVNKDAFPRIKLNPNPLAYNGPVTLYLSRYDTELRKDVLCVH
```

```
BBaV (JKT9891)     HLFGSKKQEFEACYNNGKGLLNCHNLERSKYDIDSAELIGTLIRIPLVNKDAFPRIKLNPNPLAYNGPVTLYLSRYDTELRKDVLCVH
BBaV (JKT9853)     HLFGSKKQEFEACYNNGKGLLNCHNLERSKYDIDSAELIGTLIRIPLVNKDAFPRIKLNPNPLAYNGPVTLYLSRYDTELRKDVLCVH
BBaV (JKT7774)     HLFGSKKQEFEACYNNGKGLLNCHNLERSKYDIDSAELIGTLIRIPLVNKDAFPRIKLNPNPLAYNGPVTLYLSRYDTELRKDVLCVH
KSaV (JKT10701)    HLFGSKKQEFEACYNNGKGLLNCHNLERSKYDIDSAELIGTLIRIPLINKNAFPHIKLHPAPLTYNGPVTLYLSRYDTELQKDVLCVH
NseV               HLFGSNKNDFLVCYNNGKGLEKCKNLERSKYDIDSAELIGTLIKIPLVDRNSVVDIELHPEPLTYNGPVTLYLSRYDSEQGKDVLCVH
MenoV              HLFGSNQKDFDSHYANGKGLNKCKNLERSKYDIDNAELIGTLIRIPLVDPKSVPDVKIHPKPLSYTGPVTLYLSRYDSETAKDVLCVH
                   ****.::: *   * ***** :*:**:*******.********:*** :  :.  :.::* **:*.***********:*  *******
HouV (16757)       TGFMSEGHHDIKTVFGDCGGMLFDPKGRLLGLHCAGSDDVVFMDTTTGKSNIWTSYKLQHPSEIMITLNNEINLPNPTNYDFETTKVV
HouV (V3872)       TGFMSEGHHDIKTVFGDCGGMLFDPKGRLLGLHCAGSDDVVFMDTTTGKSNIWTSYKLQHPSEIMITLNNEINLPNPTNYDFETTKVV
HouV (16740)       TGFMSEGHHDIKTVFGDCGGMLFDPKGRLLGLHCAGSDDVVFMDTTTGKSNIWTSYKLQHPSEIMITLNNEINLPNPTNYDFETTKVV
HouV (V3982)       TGFMSEGHHDIKTVFGDCGGMLFDPKGRLLGLHCAGSDDVVFMDTTTGKSNIWTSYKLQHPSEIMITLNNEINLPNPTNYDFETTKVV
NDiV               TGFMSEGHHDIKTVFGDCGGMLFDPKGRLLGLHCAGSDDVVFMDTTTGKSNIWTSYKLQHPSEIMITLNNEINLPNPTNYDFETTKVV
NgeV (JKT9982)     TGFMSEGHHDIKTVFGDCGGMLFDPKGRLLGLHCAGSDDVVFMDTTTGKSNIWTSYKLQHPSEIMITLNNEINLPNPTNYDFETTKVV
CavV               TGFMSEGHHDIKTVFGDCGGMLFDPKGRLLGLHCAGSDDVVFMDTTTGKSNIWTSYKLQHPSEIMITLNNEINLPNPANYDFETTKVV
HanaV              TGFISEGHHDIKTVFGDCGGMLFDPKGRLLGLHCAGSDDVVFMDIKTGKSNIWTSYKLQHPSEIMITLNNEINLPNPENYDFETSSVV
KPhV (KP84_0156)   TGFISEGHHDIKTVFGDCGGMLFDPKGRLLGLHCAGSDDVVFMDLNTHKSNIWTSYKLQHPSEIMITLNSEINLPNPENYDFETSKVV
KPhV (KP84_0192)   TGFISEGHHDIKTVFGDCGGMLFDPKGRLLGLHCAGSDDVVFMDLNTHKSNIWTSYKLQHPSEIMITLNSEINLPNPENYDFETSKVV
KphV (KP84_0344)   TGFISEGHHDIKTVFGDCGGMLFDPKGRLLGLHCAGSDDVVFMDLNTHKSNIWTSYKLQHPSEIMITLNSEINLPNPENYDFETSKVV
BBaV (JKT9876)     TGFISEGHHDIKTVFGDCGGMLFDPKGRLLGLHCAGSDDVVFMDLNTQKSNIWTSYKLQHPSEIMITLNSEINLPNPKNYDFETSKVV
BBaV (JKT9891)     TGFISEGHHDIKTVFGDCGGMLFDPKGRLLGLHCAGSDDVVFMDLNTQKSNIWTSYKLQHPSEIMITLNSEINLPNPKNYDFETSKVV
BBaV (JKT9853)     TGFISEGHHDIKTVFGDCGGMLFDPKGRLLGLHCAGSDDVVFMDLNTQKSNIWTSYKLQHPSEIMITLNSEINLPNPKNYDFETSKVV
BBaV (JKT7774)     TGFISEGHHDIKTVFGDCGGMLFDPKGRLLGLHCAGSDDVVFMDLNTQKSNIWTSYKLQHPSEIMITLNSEINLPNPKNYDFETSKVV
KSaV (JKT10701)    TGFISEGHHDIKTVFGDCGGMLFDPKGRLLGLHCAGSDDVVFMDLNTQKSNIWTSYKLQHPSEIMITLNSEINLPNPKNYDFETSKVV
NseV               TGFMSEGHHDIRTVFGDCGGMLFDTKGRLLGLHCAGSEDVVFMDKLQGKSNIWTSYKMQHPSEIMITLNNEINLPEPVDYDFNTNKVI
MenoV              TGFIAEGHHDIRTVFGDCGGMLFDPRGQLLGLHCAGSADVSFLNCTTGTPNIWTSYKIQHPSEIMITLDNGINLPQPEDYDFYTAKIC
                   ***::******:************.:*:********* ** *::    ..*******:*********:. ****:* :*** * .:

HouV (16757)       YQHPLRNVCATLETLQHLTNKTNAKLPYDPRLLSDFNITAEQYAQYGYNIDYNNFVNNFNRYTTTTIGTKSFETCIKYGLMDNKK-VE
HouV (V3872)       YQHPLRNVCATLETLQHLTNKTNAKLPYDPRLLSDFNITAEQYAQYGYNIDYNNFVNNFNRYTTTTIGTKSFETCIKYGLMDNKK-VE
HouV (16740)       YQHPLRNVCATLETLQHLTNKTNAKLPYDPRLLSDFNITAEQYAQYGYNIDYNNFVNNFNRYTTTTIGTKSFETCIKYGLMDNKK-VE
HouV (V3982)       YQHPLRNVCATLETLQHLTNKTNAKLPYDPRLLSDFNITAEQYAQYGYNIDYNNFVNNFNRYTTTTIGTKSFETCIKYGLMDNKK-VE
NDiV               YQHPLRNVCATLETLQHLTNKTNVKLPYDPRLLSDFNITAEQYAQYGYNIDYNNFINNFNRYTTTTIGTKSFETCIKYGLMDNKK-VE
NgeV (JKT9982)     YQHPLRNVCATLETLQHLTNKTNAKLPYDPRLLSDFNITAEQYAQYGYNIDYNNFINNFNRYTTTTIGTKSFETCIKYGLMDNKK-VE
CavV               YQHPLRNVCATLETLQHLTNKTNAKLPYDSRLLSDFNITAEQYNQYGYYIDYNNFVNNFNRYTTTTIGTKSFETCIKYGLMDNKK-PD
HanaV              YQHPLRNISATLETLQYLTNKTNAKIAYDSRLLSDFNITAEQYAQHGYYVDYNNFVNNFNHYTTTTIGTKSFETCIKYGLLNNKK-VE
KPhV (KP84_0156)   YHHPLRNVGATLETLQHLTNKTNAKLPYDPRLLSDFNITAEQYVQHGYNVDYNNFVSNFNRYTTTTIGTKSFETCIKYGLMDNKN-VT
KPhV (KP84_0192)   YHHPLRNVGATLETLQHLTNKTNAKLPYDPRLLSDFNITAEQYVQHGYNVDYNNFVSNFNRYTTTTIGTKSFETCIKYGLMDNKN-VT
KphV (KP84_0344)   YHHPLRNVGATLETLQHLTNKTNAKLPYDPRLLSDFNITAEQYVQHGYNVDYNNFVSNFNRYTTTTIGTKSFETCIKYGLMDNKN-VT
BBaV (JKT9876)     YHHPLRNVGATLETLQHLTNKTNAKLPYDPRLLSDFNITAEQYAHHGYNVDYNNFINNFNRYTTTTIGTKSFETCIKYGLMDNKN-TT
BBaV (JKT9891)     YHHPLRNVGATLETLQHLTNKTNAKLPYDPRLLSDFNITAEQYAHHGYNVDYNNFINNFNRYTTTTIGTKSFETCIKYGLMDNKN-TT
BBaV (JKT9853)     YHHPLRNVGATLETLQHLTNKTNAKLPYDPRLLSDFNITAEQYAHHGYNVDYNNFINNFNRYTTTTIGTKSFETCIKYGLMDNKN-TT
BBaV (JKT7774)     YHHPLRNVGATLETLQHLTNKTNAKLPYDPRLLSDFNITAEQYAHHGYNVDYNNFINNFNRYTTTTIGTKSFETCIKYGLMDNKN-TT
KSaV (JKT10701)    YHHPLRNVGATLETLQHLTNKTNAKMPYDPRLLSDFNITAEQYAHHGYNVDYNNFISNFNRYTTTTIGTKSFETCIKYGLMDNKN-AT
NseV               YHHPLRNVMATLETLQYLTNSTNARMPYDKRLLSDFNITAEQYEQYGLYVDYNQFVKNFNQYTTTLIGTKTFESCIKYGITDKST-TT
MenoV              YQHPLRDVRATLQTLQYLTNQGNTKLPYDPQLLADFNITAEQYNQHGYYIDYKNFIQNFIKYTNTLINSRSFEMSIKYGVANLQTNLT
                   *:****:: ***:***:***. *.::.** :**:********* ::*  :**::*:.** :**.* *.:::** .****: : ..

HouV (16757)       YYNQTATIFNPPEHSSS-GFDNIMDVLYVFVYMFTHTHPAFYIAAACVFCLFFVKMNKYLKMILSSIIFTIPHIYVNYYYGLVYMPLK
HouV (V3872)       YYNQTATIFNPPEHSSS-GFDNIMDVLYVFVYMFTHTHPAFYIAAACVFCLFFVKMNKYLKMILSSIIFTIPHIYVNYYYGLVYMPLK
HouV (16740)       YYNQTATIFNPPEHSSS-GFDNIMDVLYVFVYMFTHTHPAFYIAAACVFCLFFVKMNKYLKMILSSIIFTIPHIYVNYYYGLVYMPLK
HouV (V3982)       YYNQTATIFNPPEHSSS-GFDNIMDVLYVFVYMFTHTHPAFYIAAACVFCLFFVKMNKYLKMILSSIIFTIPHIYVNYYYGLVYMPLK
NDiV               YYNQTATIFNPPEHSSS-GFDNTMDVLYVFVYMFTHTHPAFYIAAACVFCLFFVKMNKYLKMILSSIIFTIPHIYVNYYYGLVYMPLK
NgeV (JKT9982)     YYNQTATIFNPPEHSSS-GFDNTMDVLYVFVYMFTHTHPAFYIAAACVFCLFFVKMNKYLKMILSSIIFTIPHIYVNYYYGLVYMPLK
CavV               YYNQSATIFNSPEHSSS-GFDNTMDVLYVFVYMFTHTHPAFYIAAACVFGLFFVKMNRHLKMILSSIIFAIPHIYVNYYYGLVYMPLK
HanaV              YHNQASNPFNPSEPSSSNGFDNVMDLLYVFVYMFTHTHPAFYIAAVCVFCLFFIKMNRHLKLIISSIIFAIPHIYVNYYYGLFYMPLK
KPhV (KP84_0156)   YNNQSATSVSVVDAPTR--LDYILDVLYVLLYMFTHTHPAFYIAAFCVFCLFFVKMNKYLKMVLITIIFMIPDLYINYYYGLLYLPVK
KPhV (KP84_0192)   YNNQSATSVSVVDAPTR--LDYILDVLYVLLYMFTHTHPAFYIAAFCVFCLFFVKMNKYLKMVLITIIFMIPDLYINYYYGLLYLPVK
KphV (KP84_0344)   YNNQSATSVSVVDAPTR--LDYILDVLYVLLYMFTHTHPAFYIAAFCVFCLFFVKMNKYLKMVLITIIFMIPDLYINYYYGLLYLPVK
BBaV (JKT9876)     YNNQSNTSVSIMESYSC--LDYIMDVLYVLLYMFTHTHPAYYIAAICVFCLFFVKMNKYLKMILINIILILPDLYFNYYYSLLYLPLK
BBaV (JKT9891)     YNNQSNTSVSIMESYSC--LDYIMDVLYVLLYMFTHTHPAYYIAAICVFCLFFVKMNKYLKMILINIILILPDLYFNYYYSLLYLPLK
BBaV (JKT9853)     YNNQSNTSVSIMESYSC--LDYIMDVLYVLLYMFTHTHPAYYIAAICVFCLFFVKMNKYLKMILINIILILPDLYFNYYYSLLYLPLK
BBaV (JKT7774)     YNNQSNTSVSIMESYSC--LDYIMDVLYVLLYMFTHTHPAYYIAAICVFCLFFVKMNKYLKMILINIILILPDLYFSYYYSLLYLPLK
KSaV (JKT10701)    YHNQNTTSVAIVEKYSC--LDYIMDVIYVLLYMFTHTHPAYYIAAFCVFCLFFVKMNKYLKMILINIILMLPDLYLNYYYSLLYLPLK
NseV               YRNQSNPLMLPTPVNNG--FDDIMDMLYVFIYMFTQIHPSFYVAAVAVVVLLFIKMNKVLKLTLITLICIIPHIYINSYLGLLYLPVK
MenoV              NQSETQLAINNPIAKLATDYTYLLDLLYIIIYMLSNRSPLHIICALVSMIIIFIKMDKRIKVTLLSLCYAIPLFYYRIYIGLICIPLR
                     .:    .             :*::*:::**:::  * . :.*   . ::*:**:: :*: : .:   :* :*   * .*. :*::

HouV (16757)       WRKQITALAIRYNP-YTAVALRYNKNLNIAKDVAKELGTPKNLCTHLSTLLKCIKPYAAFNDLSQVINNVDDLMANWANTYNAEELLK
HouV (V3872)       WRKQITALAIRYNP-YTAVALRYNKNLNIAKDVAKELGTPKNLCTHLSTLLKCIKPYAAFNDLSQVINNVDDLMANWANTYNAEELLK
HouV (16740)       WRKQITALAIRYNP-YTAVALRYNKNLNIAKDVAKELGTPKNLCTHLSTLLKCIKPYAAFNDLSQVINNVDDLMANWANTYNAEELLK
HouV (V3982)       WRKQITALAIRYNP-YTAVALRYNKNLNIAKDVAKELGTPKNLCTHLSTLLKCIKPYAAFNDLSQVINNVDDLMANWANTYNAEELLK
NDiV               WRKQITALAIRYNP-YTAVALRYNKNLNIAKDVAKELGTPKNLCTHLSTLLKCIKPYAAFNDLSQVINNVDDLMANWANTYNAEELLK
NgeV (JKT9982)     WRKQITALAIRYNP-YTAVALRYNKNLNIAKDVAKELGTPKNLCTHLSTLLKCIKPYAAFNDLSQVINNVDDLMANWANTYNAEELLK
CavV               WRKQITALAIRYNP-YTAVAIRYNKNLNIAKDVAKELGTPKNLCTHLSTLLKCIKPYAAFNDLSQVINNVDDLMANWANTYNPEKLLK
HanaV              WRKHITALATRYNP-YTAVATRYNKNLNIAKDMAKELGTPKNLCTHLATLLKCIKPYPAFSELSQVVNNVDDLMANWANTYNAEELLK
KPhV (KP84_0156)   WRYQIAHHIMSYKT-TTALALRYNENSTIAKDLAKELGTSKNLCTHLATILKCIKPYSAFNDLSQVVNNVDDLMANWADVHNAEELLK
KPhV (KP84_0192)   WRYQIAHHIMSYKT-TTALALRYNENSTIAKDLAKELGTSKNLCTHLATILKCIKPYSAFNDLSQVVNNVDDLMANWADVHNAEELLK
KphV (KP84_0344)   WRYQIAHHIMSYKT-TTALALRYNENSTIAKDLAKELGTSKNLCTHLATILKCIKPYSAFNDLSQVVNNVDDLMANWADVHNAEELLK
BBaV (JKT9876)     WRYEISYQFMRYKAYTTAIAVRYNESLTVAKDLAKELGTPKNLCTHLATILKCIKPYAAFSDLSQVVNNVDDLMANWVNTHNATELLK
```

```
BBaV (JKT9891)      WRYEISYQFMRYKAYTTAIAVRYNESLTVAKDLAKELGTPKNLCTHLATILKCIKPYAAFSDLSQVVNNVDDLMANWVNTHNATELLK
BBaV (JKT9853)      WRYEISYQFMRYKAYTTAIAVRYNESLTVAKDLAKELGTPKNLCTHLATILKCIKPYAAFSDLSQVVNNVDDLMANWVNTHNATELLK
BBaV (JKT7774)      WRYEISYQFMRYKAYTTAIAVRYNESLTVAKDLAKELGTPKNLCTHLATILKCIKPYAAFSDLSQVVNNVDDLMANWVNTHNATELLK
KSaV (JKT10701)     WRCEI-YHLIRHKAYTTSIAVRYNENLTIAKDLAKELGTPKNLCTHLATILKCIKPYAAFSDLSQVVNNVDDLMANWVNIHNASELLK
NseV                WRAQICGLKYKKLNSTAMTTTYDNQNLKIAEGVARELGTRKNLCTHLSTLLKCIKPYPAFSELSQVVNNVDDLMANWNSTHNAEELLQ
MenoV               LTALPAFTKP-----LTALNVYRNKNLKLAKTLATELGTSKNLCIQLATLLKCIKPYPAFSELEQVVNNVDDLMAKWDQTHDAEAVLK
                               :      *:. .:*: :* **** **** :*:*:*******.**.:*.**:********:* . ::.  :*:
HouV (16757)        QYIDEIYKLYPILFVVFEKIENYEDQIKTILSYISDTGEFDLNGFEIHFDEKEHTTNIVDTN--VEDIHDKLMAEKASLIALKNMNLE
HouV (V3872)        QYIDEIYKLYPILFVVFEKIENYEDQIKTILSYISDTGEFDLNGFEIHFDEKEHTTNIVDTN--VEDIHDKLMAEKASLIALKNMNLE
HouV (16740)        QYIDEIYKLYPILFVVFEKIENYEDQIKTILSYISDTGEFDLNGFEIHFDEKEHTTNIVDTN--VEDIHDKLMAEKASLIALKNMNLE
HouV (V3982)        QYIDEIYKLYPILFVVFEKIENYEDQIKTILSYISDTGEFDLNGFEIHFDEKEHTTNIVDTN--VEDIHDKLMAEKASLIALKNMNLE
NDiV                QYIDEIYKLYPILFVVFEKIENYEDQIKTILSYISDTGEFDLNGFEIHFDEKEHTTNIIDTN--VEDIHDKLMAEKASLIALKNMNLE
NgeV (JKT9982)      QYIDEIYKLYPILFVVFEKIENYEDQIKTILSYISDTGEFDLNGFEIHFDEKEHTTNIIDTN--VEDIHDKLMAEKASLIALKNMNLE
CavV                QYIEEIYKLYPILFIVFERIESYEDQIKTILSFISDTGEFDLNGFEIHFDEKEHTTNIIDTN--VEDIREKLMAAKASLIALKNMNSE
HanaV               QYIDEIYKLYPILFVVFEKIENYEDQIKAILAYISDTGEFDLNGFTIHFDEKEHTINTTDTN--VEDIHEKLMAEKASIIALKNMNQE
KPhV (KP84_0156)    QYIDEIYKLYPILFVVFEKIETVEEQIKTILSYISDTGEFDLNGFEIHFDEKEHTTNIIDTN--ADEIHTKLLEEKASFIALKNMNSD
KPhV (KP84_0192)    QYIDEIYKLYPILFVVFEKIETVEEQIKTILSYISDTGEFDLNGFEIHFDEKEHTTNIIDTN--ADEIHTKLLEEKASFIALKNMNSD
KphV (KP84_0344)    QYIDEIYKLYPILFVVFEKIETVEEQIKTILSYISDTGEFDLNGFEIHFDEKEHTTNIIDTN--ADEIHTKLLEEKASFIALKNMNSD
BBaV (JKT9876)      EYIDEIYKLYPILFVVFEKIESVEEQIKVVLSYISDTGEFDMNGFEIHFDEKEHTTTITDPN--AALLHERLMAEKASFIALKNMNSQ
BBaV (JKT9891)      EYIDEIYKLYPILFVVFEKIESVEEQIKVVLSYISDTGEFDMNGFEIHFDEKEHTTTITDPN--AALLHERLMAEKASFIALKNMNSQ
BBaV (JKT9853)      EYIDEIYKLYPILFVVFEKIESVEEQIKVVLSYISDTGEFDMNGFEIHFDEKEHTTTITDPN--AALLHERLMAEKASFIALKNMNSQ
BBaV (JKT7774)      EYIDEIYKLYPILFVVFEKIESVEEQIKVVLSYISDTGEFDMNGFEIHFDEKEHTTTITDPN--AALLHERLMAEKASFIALKNMNSQ
KSaV (JKT10701)     EYIDEIYKLYPILFVVFEKIETVEDQIKTVLSYINDTGEFDLNGFEIHFDEKEHTTNIIDPN--AAEIYEKLMVEKASFIALKNMNSE
NseV                QYIDEIYKLYPILFVVFEKIDNYDLQIRTILAYISDAGEFDLKGFSIHFDENEHTTEIVGTSNETEHLANRLMDAKASIIALKNMNVE
MenoV               QNIDEIYKLYPILFIVFEQVEKYDDQIKLIMSYINDTGNFDLNGFEINYDENEHTTKIIDPA--LDNLQMELVEKQDCLVTLRNMNSE
                    : *:*********:***::. : **: ::::*.*:*:**::** *::**:***     ..      :  .*:  : .:::*:*** :

HouV (16757)        FDIETINNANIGELVRYLIISSTPDTLDRDLLSRTTELLVRHIHQLRDNSEHNENLITLLSEIYKHKDFLTASHLTSNLRDRNYIMNN
HouV (V3872)        FDIETINNANIGELVRYLIISSTPDTLDRDLLSRTTELLVRHIHQLRDNSEHNENLITLLSEIYKHKDFLTASHLTSNLRDRNYIMNN
HouV (16740)        FDIETINNANIGELVRYLIISSTPDTLDRDLLSRTTELLVRHIHQLRDNSEHNENLITLLSEIYKHKDFLTASHLTSNLRDRNYIMNN
HouV (V3982)        FDIETINNANIGELVRYLIISSTPDTLDRDLLSRTTELLVRHIHQLRDNSEHNENLITLLSEIYKHKDFLTASHLTSNLRDRNYIMNN
NDiV                FDIETINNANIGELVRYLIISSTPDTLDRDLLSRTTELLVRRIHQLRDDSEHNENLITLLSEIYKHKDFLTASHLTSNLRDRNYIMNN
NgeV (JKT9982)      FDIETINNANIGELVRYLIISSTPDTLDRDLLSRTTELLVRHIHQLRDDSEHNENLITLLSEIYKHKDFLTASHLTSNLRDRNYIMNN
CavV                FDIETINSANIGELVRYLIISSTPETLDRDLLARTTELLVRHIHQLRDDSEHNENLITLLSEIYKHKDFLTASHLTSNLRDRNYVMNN
HanaV               FDIETINNANIGELVRYLIISSNPETLDRDLLSRTAELLARHIHHLRENAEHNDNLITLLAEIYKHKDFLTASHLTSNLRDRNYIMNN
KPhV (KP84_0156)    FNVESINTASIGELVRYLIISSTPETLDRDLLARTTEMLVRQIHNLRENNEHNENLIALLSEIYKHKDFLTASYLTSNLRDRNYVMNN
KPhV (KP84_0192)    FNVESINTASIGELVRYLIISSTPETLDRDLLARTTEMLVRQIHNLRENNEHNENLIALLSEIYKHKDFLTASYLTSNLRDRNYVMNN
KphV (KP84_0344)    FNVESINTASIGELVRYLIISSTPETLDRDLLARTTEMLVRQIHNLRENNEHNENLIALLSEIYKHKDFLTASYLTSNLRDRNYVMNN
BBaV (JKT9876)      FNIDTINDASIGELVRYLIISSTPETLDRELLARTTEMLVRHINSLRDNNEHSENLITLLSEIYKHKDFLTASHLTSNLRDRNYVMNN
BBaV (JKT9891)      FNIDTINDASIGELVRYLIISSTPETLDRELLARTTEMLVRHINSLRDNNEHSENLITLLSEIYKHKDFLTASHLTSNLRDRNYVMNN
BBaV (JKT9853)      FNIDTINDASIGELVRYLIISSTPETLDRELLARTTEMLVRHINSLRDNNEHSENLITLLSEIYKHKDFLTASHLTSNLRDRNYVMNN
BBaV (JKT7774)      FNIDTINDASIGELVRYLIISSTPETLDRELLARTTEMLVRHINSLRDNNEHSENLITLLSEIYKHKDFLTASHLTSNLRDRNYVMNN
KSaV (JKT10701)     FNINSINEASIGELVRYLIISSTPETLDRELLARTTELLVRQIHNLRDNNDHNENLIALLSEIYKHKDFLTASHLTSNLRDRNYIMNN
NseV                FDIETLNDANIGELVRYMIVSSTPETLDREILTRATEMLVRYINRIRENNETNEQLIPLLSEIYKHKDFLTAAHLTSNVKDKNFILNN
MenoV               FDISSIQSANIGELVRYLIVSSTPETLNRELLTQATEALVQHITTLRNSNDHSENLIPLLSEIYKHKDLLTSSYLTAHAKDKNFVMNS
                    *::.::: *.*******:*:**.*:**:*::*::::* *.: *  :*:. : .::**.**:*******:**:::**:: :*:*:::*.

HouV (16757)        LVRVIALFNKQINMQVAQKQYEARRIEDLRKKESKQIMEQNNRIRKMQRQNQNIASAIVHMVHACFANRFMLQNEAQKIMKALLGSDL
HouV (V3872)        LVRVIALFNKQINMQVAQKQYEARRIEDLRKKESKQIMEQNNRIRKMQRQNQNIASAIVHMVHACFANRFMLQNEAQKIMKALLGSDL
HouV (16740)        LVRVIALFNKQINMQVAQKQYEARRIEDLRKKESKQIMEQNNRIRKMQRQNQNIASAIVHMVHACFANRFMLQNEAQKIMKALLGSDL
HouV (V3982)        LVRVIALFNKQINMQVAQKQYEARRIEDLRKKESKQIMEQNNRIRKMQRQNQNIASAIVHMVHACFANRFMLQNEAQKIMKALLGSDL
NDiV                LVRVIALFNKQINMQVAQKQYEARRIEELRKKESKQIMEQNNRIRKMQRQNQNIASAIVHMVHACFANRFMLQNEAQKIMKALLGSDL
NgeV (JKT9982)      LVRVIALFNKQINMQVAQKQYEARRIEELRKKESKQIMEQNNRIRKMQRQNQNIASAIVHMVHACFANRFMLQNEAQKIMKALLGSDL
CavV                LVRVIALFNKQINMQVAQKQYEARRIEDVRKKESKQIMEQNNRIRKMQRQNQNIASAIVHMVHACFANRFMLQNEAQKIMKALLGTNL
HanaV               LIRVIALFNKQINFQVTQKQYEARRIEEERRKESKQIMEQNNRIRKMQRQNQNIASAIVHMVHACFANRFMLQNESQKIMKALLGTTL
KPhV (KP84_0156)    LVRVIALFNKQINMQVTQKQYEARRIENERKKESKQIMEQNNRIRKMQRQNQNIASAIVHMVHACFANRFMLKNEAQLIMQELTGNNL
KPhV (KP84_0192)    LVRVIALFNKQINMQVTQKQYEARRIENERKKESKQIMEQNNRIRKMQRQNQNIASAIVHMVHACFANRFMLKNEAQLIMQELTGNNL
KphV (KP84_0344)    LVRVIALFNKQINMQVTQKQYEARRIENERKKESKQIMEQNNRIRKMQRQNQNIASAIVHMVHACFANRFMLKNEAQLIMQELTGNNL
BBaV (JKT9876)      LVRVIALFNKQINMQVAQKQHEARRVENERKKESKQIMEQNNRIRKMQRQNQNIASAIVHMVHACFANRFMLKNEAQLIMKELAVYQL
BBaV (JKT9891)      LVRVIALFNKQINMQVAQKQHEARRVENERKKESKQIMEQNNRIRKMQRQNQNIASAIVHMVHACFANRFMLKNEAQLIMKELAVYQL
BBaV (JKT9853)      LVRVIALFNKQINMQVAQKQHEARRVENERKKESKQIMEQNNRIRKMQRQNQNIASAIVHMVHACFANRFMLKNEAQLIMKELAVYQL
BBaV (JKT7774)      LVRVIALFNKQINMQVAQKQHEARRVENERKKESKQIMEQNNRIRKMQRQNQNIASAIVHMVHACFANRFMLKNEAQLIMKELAVYQL
KSaV (JKT10701)     LVRVIALFNKQINMQVAQKQYEARRVENERKKESKQIMEQNNRIRKMQRQNQNIASAIVHMVHACFANRFMLKNEAQLIMKELATYQL
NseV                LIRVIALFNKQINLQVQQKQIEARRIEDERKKESKLIMEQNNRIRKMQRQNQNIASAIVHMVHACFANRFMLQNESRKIMKALIGLNL
MenoV               LVRVIALFNKQINLQVQQATIEAKKVERARMAESKTIIEQNNRIRKIQRQNQNIASAIVHMVHACFANRFMLQNEARKITKALAGTNV
                    *:***********:** *    **:::*  *  *** *:********:************************:**:: * : *    :

HouV (16757)        ELDPTDAEMQYYTAYRNG-QVLTNQAIVTNFTTLTTILWTGNGYQTVPSMCGAQEFTCTATHKHGYFNCTMEIKDAWYKHAEECTKCK
HouV (V3872)        ELDPTDAEMQYYTAYRNG-QVLTNQAIVTNFTTLTTILWTGNGYQTVPSMCGAQEFTCTATHKHGYFNCTMEIKDAWYKHAEECTKCK
HouV (16740)        ELDPTDAEMQYYTAYRNG-QVLTNQAIVTNFTTLTTILWTGNGYQTVPSMCGAQEFTCTATHKHGYFNCTMEIKDAWYKHAEECTKCK
HouV (V3982)        ELDPTDAEMQYYTAYRNG-QVLTNQAIVTNFTTLTTILWTGNGYQTVPSMCGAQEFTCTATHKHGYFNCTMEIKDAWYKHAEECTKCK
NDiV                ELDPTDAEMQYYTAYRNG-QVLTNQAIVTNFTTLTTILWTGNGYQTVPSMCGAQEFTCTATHKHGYFNCTMEIKDAWYKHAEECTKCK
NgeV (JKT9982)      ELDPTDAEMQYYTAYRNG-QVLTNQAIVTNFTTLTTILWTGNGYQTVPSMCGAQEFTCTATHKHGYFNCTMEIKDAWYKHAEECTKCK
CavV                ELDPSDAEMQYYTAYRTG-QVLTNQAIVTNFTTLTTILWTGNGYQTVPSMCGLHEFTCTATHKHGYFNCTMEIKDAWYKHAEECTKCK
HanaV               ELDPTDAEMQHYAAYRNG-QVLTNHVIITNFTTLTTILWTGNGYQIVPSMCGQEEFTCTATHKHGYFNCTMEIKDAWYKHAEECAKCK
KPhV (KP84_0156)    DLDPTDAEMLHYEAYRSG-QVLTNKSIKTNFTTLSTILWTGNGYQTVPSMCGDHEFSCTASHKHGYFNCTMEIKDDWYTHVENCAKCR
KPhV (KP84_0192)    DLDPTDAEMLHYEAYRSG-QVLTNKSIKTNFTTLSTILWTGNGYQTVPSMCGDHEFSCTASHKHGYFNCTMEIKDDWYTHVENCAKCR
KphV (KP84_0344)    DLDPTDAEMLHYEAYRSG-QVLTNKSIKTNFTTLSTILWTGNGYQTVPSMCGDHEFSCTASHKHGYFNCTMEIKDDWYTHVENCAKCR
BBaV (JKT9876)      ELDPSDAEMRHYESYQQD-TVLTNKAIITNFTTLSTILWTGNGYQTVPSMCGDHEFTCTASHKHGYFNCTMEIKDEWYTHVENCDKCR
```

```
BBaV (JKT9891)     ELDPSDAEMRHYESYQQD-TVLTNKAIITNFTTLSTILWTGNGYQTVPSMCGDHEFTCTASHKHGYFNCTMEIKDEWYTHVENCDKCR
BBaV (JKT9853)     ELDPSDAEMRHYESYQQD-TVLTNKAIITNFTTLSTILWTGNGYQTVPSMCGDHEFTCTASHKHGYFNCTMEIKDEWYTHVENCDKCR
BBaV (JKT7774)     ELDPSDAEMRHYESYQQD-TVLTNKAIITNFTTLSTILWTGNGYQTVPSMCGDHEFTCTASHKHGYFNCTMEIKDEWYTHVENCDKCR
KSaV (JKT10701)    NLDPSDAEMIHYESYQHD-TVLTNKSIQTNFTTLSTILWTGNGYQTVPSMCGDHEFTCTASHKHGYFNCTMEIKDDWYNHVENCDKCR
NseV               ELDPSEPESQYYAERRAG-KCYSNQTIVTNFTTLTTILWTGNGYQKLPSMCGDAEFVCTQQHKHGYFNCTMEIKDAWYKHVEECPKCK
MenoV              EFELTEAESDYYYQGRVKNTVMNNQAITTNFTTLSTVLWTGNGYQKLPSMCGSEEFVCTLPHKHGYFNCTMQIKDAWYAHVDACERCR
                   ::: ::.*  :*   :      .*: * ******:*:******** :*****  ** **  **********:*** ** *.: * :*:
HouV (16757)       SYYRTNKHPRCGAIYDTTVKRYPTLSNFIARYRSCPACMPCTQCLSRREPGCESASYHIADTAHYQNQAYLTPINIKPDNLEYNFVDI
HouV (V3872)       SYYRTNKHPRCGAIYDTTVKRYPTLSNFIARYRSCPACMPCTQCLSRREPGCESASYHIADTAHYQNQAYLTPINIKPDNLEYNFVDI
HouV (16740)       SYYRTNKHPRCGAIYDTTVKRYPTLSNFIARYRSCPACMPCTQCLSRREPGCESASYHIADTAHYQNQAYLTPINIKPDNLEYNFVDI
HouV (V3982)       SYYRTNKHPRCGAIYDTTVKRYPTLSNFIARYRSCPACMPCTQCLSRREPGCESASYHIADTAHYQNQAYLTPINIKPDNLEYNFVDI
NDiV               SYYRTNKHPRCGAIYDTTVKRYPTLSNFIARYRSCPACMPCTQCLSRREPGCESASYHIADTAHYQNQAYLTPINIKPDNLEYNFVDI
NgeV (JKT9982)     SYYRTNKHPRCGAIYDTTVKRYPTLSNFIARYRSCPACMPCTQCLSRREPGCESASYHIADTAHYQNQAYLTPINIKPDNLEYNFVDI
CavV               SYYRANKHPRCGAIYDTTVKRIPTLSNFIARYRSCPSCMPCTQCLSHREPGCESASYHIADTAHYQNQAYLTPINIKPDNLEYNFVDV
HanaV              VYYRANRHPRCGAVYDSTVKRYPTLSNFIARYRSCPSCMPCTQCLSHREPGCESASYHIADTAHYQNQAYLTPINIKPDNLEYTFTDI
KPhV (KP84_0156)   SYYKANKHPRCEAIYSIDVKRFPTLSNFVSRYRSCPACLPCTSCISTREPTCTIASYHVDDTAHYKNEAYLTPLNIKPDNLTYSFVNS
KPhV (KP84_0192)   SYYKANKHPRCEAIYSIDVKRFPTLSNFVSRYRSCPACLPCTSCISTREPTCTIASYHVDDTAHYKNEAYLTPLNIKPDNLTYSFVNS
KphV (KP84_0344)   SYYKANKHPRCEAIYSIDVKRFPTLSNFVSRYRSCPACLPCTSCISTREPTCTIASYHVDDTAHYKNEAYLTPLNIKPDNLTYSFVNS
BBaV (JKT9876)     NYYKARKHPRCPLTYDADKKRFPTLSNFIARYRSCPRCLPCTSCLSKREPTCTLNSYHVDDSAHYKNEAYLTPLNIKPDTLVYDFVNS
BBaV (JKT9891)     NYYKARKHPRCPLTYDADKKRFPTLSNFIARYRSCPRCLPCTSCLSKREPTCTLNSYHVDDSAHYKNEAYLTPLNIKPDTLVYDFVNS
BBaV (JKT9853)     NYYKARKHPRCPLTYDADKKRFPTLSNFIARYRSCPRCLPCTSCLSKREPTCTLNSYHVDDSAHYKNEAYLTPLNIKPDTLVYDFVNS
BBaV (JKT7774)     NYYKARKHPRCPLTYDADKKRFPTLSNFIARYRSCPRCLPCTSCLSKREPTCTLNSYHVDDSAHYKNEAYLTPLNIKPDTLVYDFVNS
KSaV (JKT10701)    SYYKQRKHPRCPNTYDPDTKRFPTLSNFIARYRSCPCCLPCTSCLSKREPTCTINSYHVEDTAYYKNEAYLTPLNIKPDNLTYDFTSP
NseV               NYYRQNKHPRCGGLYPSDVKRYPTLSNFISRYRSCPSCMPCTQCLSRREHNCENDSYHFSDATQVANQAYLTPINIKPDSLEYNFVDV
MenoV              TYYKNNRHPRCGAIYPADLKRYATLSNFVSRYHSCPQCLPCTQCLSIRESTCSINCYHPSDNSVIINQAYLTPLNIKPDSLEYTFIDK
                    **: .:****   *    ** .*****::**:*** *:***.*:* **  *    .**  * :   *:*****:*****.* * * .

HouV (16757)       NNGDVNAIYNGRIWLMRRTTAITPPPARYRNITNLKLKQTDPEGYYYISEVCPTDLAILNAMINQIQLKLLDRTVLNNENH----VEN
HouV (V3872)       NNGDVNAIYNGRIWLMRRTTAITPPPARYRNITNLKLKQTDPEGYYYISEVCPTDLAILNAMINQIQLKLLDRTVLNNENH----VEN
HouV (16740)       NNGDVNAIYNGRIWLMRRTTAITPPPARYRNITNLKLKQTDPEGYYYISEVCPTDLAILNAMINQIQLKLLDRTVLNNENH----VEN
HouV (V3982)       NNGDVNAIYNGRIWLMRRTTAITPPPARYRNITNLKLKQTDPEGYYYISEVCPTDLAILNAMINQIQLKLLDRTVLNNENH----VEN
NDiV               NNGDVNAIYNGRIWLMRRTTAITPPPARYRNITNLKLKQTDPEGYYYISEVCPTDLAILNAMINQIQLKLLDRTVLNNENH----VEN
NgeV (JKT9982)     NNGDVNAIYNGRIWLMRRTTAITPPPARYRNITNLKLKQTDPEGYYYISEVCPTDLAILNAMINQIQLKLLDRTVLNNENH----VEN
CavV               NNGDVNAIYNGRIWLMRRTTAITPPPARYRNITNLKLKQTDPEGYYYISEVCPTDLAILNAMINQIQLKLLDRTVLNNENH----VEN
HanaV              ASGDVNAIYNGRIWLMRRTTAITPPPARYRNITNLKLKQTDPEGYYYISEVCPTDLAILNAMINQIQLKLLDRVNLNNETH----VEE
KPhV (KP84_0156)   VSGDVNAIYNGRVWLMRRTTPATPPPARYRHITNLKLQQADPEGYYYISDVCPTDLAILNAMINQIQLKLQENIALNNQNN----YEE
KPhV (KP84_0192)   VSGDVNAIYNGRVWLMRRTTPATPPPARYRHITNLKLQQADPEGYYYISDVCPTDLAILNAMINQIQLKLQENIALNNQNN----YEE
KphV (KP84_0344)   VSGDVNAIYNGRVWLMRRTTPATPPPARYRHITNLKLQQADPEGYYYISDVCPTDLAILNAMINQIQLKLQENIALNNQNN----YEE
BBaV (JKT9876)     VNEDINAIYNGRIWLMRRSTPVTPPPARYRHITNLKLQQADPEGYYYLSEHCPTNLAILNAMINQIQVKLQERTTLNNENA----YEE
BBaV (JKT9891)     VNEDINAIYNGRIWLMRRSTPVTPPPARYRHITNLKLQQADPEGYYYLSEHCPTNLAILNAMINQIQVKLQERTTLNNENA----YEE
BBaV (JKT9853)     VNEDINAIYNGRIWLMRRSTPVTPPPARYRHITNLKLQQADPEGYYYLSEHCPTNLAILNAMINQIQVKLQERTTLNNENA----YEE
BBaV (JKT7774)     VNEDINAIYNGRIWLMRRSTPVTPPPARYRHITNLKLQQADPEGYYYLSEHCPTNLAILNAMINQIQVKLQERTTLNNENA----YEE
KSaV (JKT10701)    DSGDINANYNGRIWLMRRSTPATPPQARYRHITNLKLQQADPEGYYYLSEHCPTDLAILNAMINQIQVKLQERSILNNENA----SEE
NseV               NNGDVNAVYNGRVWLMRRTQAIIPPPSRYRNITNLKLKNLDPEGYYYISDVCPTDLAILNAMINQIQVKLQERTFVHNENF----TQE
MenoV              TIGDVNAVYNGRIWLMRRSIPISPPPARYRHITNLKLKQADPEGYYYISDVCPTDIAILNAMINQIQLKLQDCATMSNESLTLIDTQT
                      *:** ****:*****: .  ** :***:******:: *******:*: ***::***********:** :   : *:.      :

HouV (16757)       DN-TIKFNNPLNDTTLDDLRTKHKHLLVMKLRPDSEHHFIEVLNFVRMNNLPIFIAHVTYADNTVNHATIYINYLQAWRNEILDDVTT
HouV (V3872)       DN-TIKFNNPLNDTTLDDLRTKHKHLLVMKLRPDSEHHFIEVLNFVRMNNLPIFIAHVTYADNTVNHATIYINYLQAWRNEILDDVTT
HouV (16740)       DN-TIKFNNPLNDTTLDDLRTKHKHLLVMKLRPDSEHHFIEVLNFVRMNNLPIFIAHVTYADNTVNHATIYINYLQAWRNEILDDVTT
HouV (V3982)       DN-TIKFNNPLNDTTLDDLRTKHKHLLVMKLRPDSEHHFIEVLNFVRMNNLPIFIAHVTYADNTVNHATIYINYLQAWRNEILDDVTT
NDiV               AN-TIQFNNPLNDTTLDDLRTKHKHLLVMKLRPDSEHHFIEVLNFVRMNNLPIFIAHVTYADNTVNHATIYINYLQAWRNEILDDVTT
NgeV (JKT9982)     AN-TIKFNNPLNDTTLDDLRTKHKHLLVMKLRPDSEHHFIEVLNFVRMNNLPIFIAHVTYADNTVNHATIYINYLQAWRNEILDDVTT
CavV               DN-TIKFNTPLNDTTLDDLRTKHKHLLVMKLKPDSEHHFIEVLDFVRMNNLPIFIAHVTYAENDVNHATIYVNYLQAWRNEILDDVTK
HanaV              YNNTIKFNTPLTDTTLDELRTKHKHLLVLKLRQDSEYHFVDVLNFVRMNNLPIFIVHVTHEEINVNHATLYINYLQAWRNEI-DDIKT
KPhV (KP84_0156)   VTNYISFSNPLSEATLTDLRKKHTYLLILRLRPDADNHLMDVVNYVKQNQLPIFIVHVSNEIIDTNHATLYVNYMQVWR-NAIDSSNV
KPhV (KP84_0192)   VTNYISFSNPLSEATLTDLRKKHTYLLILRLRPDADNHLMDVVNYVKQNQLPIFIVHVSNEIIDTNHATLYVNYMQVWR-NAIDSSNV
KphV (KP84_0344)   VTNYISFSNPLSEATLTDLRKKHTYLLILRLRPDADNHLMDVVNYVKQNQLPIFIVHVSNEIIDTNHATLYVNYMQVWR-NAIDSSNV
BBaV (JKT9876)     PNNYIKFNNPPSDATLSDLRDKHKYLLVMRLRSDSEHHLIEVINYVRQGNLPVFIVHVTNDTITNDYATLYVSYMQVWRREIIDNIDI
BBaV (JKT9891)     PNNYIKFNNPPSDATLSDLRDKHKYLLVMRLRSDSEHHLIEVINYVRQGNLPVFIVHVTNDTITNDYATLYVSYMQVWRREIIDNIDI
BBaV (JKT9853)     PNNYIKFNNPPSDATLSDLRDKHKYLLVMRLRSDSEHHLIEVINYVRQGNLPVFIVHVTNDTITNDYATLYVSYMQVWRREIIDNIDI
BBaV (JKT7774)     PNNYIKFNNPPSDATLSDLRDKHKYLLVMRLRSDSEHHLIEVINYVRQGNLPVFIVHVTNDTITNDYATLYVSYMQVWRREIIDNIDI
KSaV (JKT10701)    LNNYIKFTTPPSDATLSDLRDKHTYLLVMRLRPDSEHHLLEVVNYVNQNQLPTFIVHVTNDTIENDHATLYVSYMQVWRREIIDNIDV
NseV               PTIDTTYRTPINEQLLSQLRENHKYLLVLKLKPDSDNLHYELLRFVKTLSFPVHVCHITYDEITTNHVAMYVNYTQVWRTTVNDNLET
MenoV              QINSTIFCKPVNDNMLDELKENHIYLLILKLQPQDEHLYIAMENYLSKLNPSIHICLIIHNEIPSNHLSLYINCMQVWRHSINHDNPE
                         : .* .:  * :*: :* :**:::*: : :     :  ::   . . .:  :       :: ::*:.  *.**    ..

HouV (16757)       TCDILEKIIKHPLD-SRWARTLSRNSNVARYHQLCTTTNAGIRHTIDISCNKTSISYIDEVNNNVNVKIKSHIVKEYKIYEMLINQ-Y
HouV (V3872)       TCDILEKIIKHPLD-SRWARTLSRNSNVARYHQLCTTTNAGIRHTIDISCNKTSISYIDEVNNNVNVKIKSHIVKEHKIYEMLINQ-Y
HouV (16740)       TCDILEKIIKHPLD-SRWARTLSRNSNVARYHQLCTTTNAGIRHTIDISCNKTSISYIDEVNNNVNVKIKSHIVKEHKIYEMLINQ-Y
HouV (V3982)       TCDILEKIIKHPLD-SRWARTLSRNSNVARYHQLCTTTNAGIRHTIDISCNKTSISYIDEVNNNVNVKIKSHIVKEYKIYEMLINQ-Y
NDiV               TCDILEKIIKHPLDFSRWARTLSRNSNVARYHQLCTTTDAGIRHTIDISCNKTSISYIDEVNNNVNVKIKSHIVKEHKIYEMLINQ-Y
NgeV (JKT9982)     TCDILEKIIKHPLD-SRWARTLSRNSNVARYHQLCTTTNAGIRHTIDISCNKTSISYIDEVNNNVNVKIKSHIVKEHKIYEMLINQ-Y
CavV               TCDILEKIIKHPLDFQSGLVLLSRNSNVARYHQLCTNTNNGIRHTIDISCNKTSISYIDEVNNNVNVKIKQHIVKEYKIYEMLINQ-Y
HanaV              TYDILENVIKNPLDFSKWARTLSRNSNVARYHQLCTNTNVGTRYTIDISCNKTSTSYIDSKNNIVNVKIKNNIVKEYNIYEMLINQ-Y
KPhV (KP84_0156)   VCDILTKVISQPMDFSTWARSLSRNSNVARYHQLCTNTKHGTRHTIDISCNKTSLSYIDEYNQTVNVKIKEHIVKEYEIYETLINQ-D
KPhV (KP84_0192)   VCDILTKVISQPMD-STWARSLSRNSNVARYHQLCTNTKHGTRHTIDISCNKTSLSYIDEYNQTVNVKIKEHIVKEYEIYETLINQ-D
KphV (KP84_0344)   VCDILTKVISQPMD-STWARSLSRNSNVARYHQLCTNTKHGTRHTIDISCNKTSLSYIDEYNQTVNVKIKEHIVKEYEIYETLINQ-D
BBaV (JKT9876)     VCDTLSKVNKKPLD-SNWARSLSRNSNVARYHQLCTNTTHGMRHTIDISCNKTSVSYIDDHNNNVNVKIKAHIVKEYEIYETLINQ-E
```

```
BBaV (JKT9891)      VCDTLSKVNKKPLD-SNWARSLSRNSNVARYHQLCTNTTHGMRHTIDISCNKTSVSYIDDHNNNVNVKIKAHIVKEYEIYETLINQ-E
BBaV (JKT9853)      VCDTLSKVNKKPLD-SNWARSLSRNSNVARYHQLCTNTTHGMRHTIDISCNKTSVSYIDDHNNNVNVKIKAHIVKEYEIYETLINQ-E
BBaV (JKT7774)      VCDTLSKVNKKPLDFSNWARSLSRNSNVARYHQLCTNTTHGMRHTIDISCNKTSVSYIDDHNNNVNVKIKAHIVKEYEIYETLINQ-E
KSaV (JKT10701)     VCDILSKVNTQPLDFSYWARSLSRNSNVARYHQLRTNTKTGIRHTIDISCNKTSVSYIDGHNNNVNVKIKAHIVKEYQIYETLINQ-E
NseV                TYEMLKRIDQTPLDFSKWARTLSRNSNVARYHQLHTKRLGVERYTIDISCNKTSTSYVNEQSQIVNVKFKAHVKYEQLVYDTLINQ-E
MenoV               SLSESVNKMTINTDFSKWARSLSRNTNVARYHQIRTVSKQNVRYTLDISCNKTSVSYITPNSEIVNVKVKPNIEKEHTIYEMLTKTSK
                      .   .      * .     ****:*******: *      *:*:******** **:   .: ****.* ::  *  :*:  * :
HouV (16757)        PNLFLIEHKLVNDTIPHLLRYNMTALSFADLYGLIKEENWHPIYDTLPQVTYHKINDDLLLKIKSHTPSPQHTCCMLCRRFLVEFGLL
HouV (V3872)        PNLFLIEHKLVNDTIPHLLRYNMTALSFADLYGLIKEENWHPIYDTLPQVTYHKINDDLLLKIKSHTPSPQHTCCMLCRRFLVEFGLL
HouV (16740)        PNLFLIEHKLVNDTIPHLLRYNMTALSFADLYGLIKEENWHPIYDTLPQVTYHKINDDLLLKIKSHTPSPQHTCCMLCRRFLVEFGLL
HouV (V3982)        PNLFLIEHKLVNDTIPHLLRYNMTALSFADLYGLIKEENWHPIYDTLPQVTYHKINDDLLLKIKSHTPSPQHTCCMLCRRFLVEFGLL
NDiV                PNLFLIEHKLVNDIIPHLLRYNMTALSFADLYGLIKEENWHPIYDTLPQVTYHKINDDLLLKIKSHTPSPQHTCCMLCRRFLVEFGLL
NgeV (JKT9982)      PNLFLIEHKLVNDTIPHLLRYNMTALSFADLYGLIKEENWHPIYDTLPQVTYHKINDDLLLKIKSHTPSPQHTCCMLCRRFLVEFGLL
CavV                PNLFLIEHKLVNFTIPHLLRYNMTALSFADLYGLIKEENWHPIYDTLPQVTYHKINDDLLLKIKSHTPSPQHTCCMLCRRFLVEFGLL
HanaV               SDLFLIEHKMVTSTIPHLLRYNMTALSFADLFGLIKDENWHPIYDTLPQVTYHNIDNDLLLKIKQHTPSPQHTCCMLCRRFLAEFGLL
KPhV (KP84_0156)    SNLFLIKHKLVHATIPHLLRYNMTALSFADLFGLIKEENWHPIYDTLPQVTYHKISNDLLTKIKSHTPSPQHTCCMLCRRFLVEFGLL
KPhV (KP84_0192)    SNLFLIKHKLVHATIPHLLRYNMTALSFADLFGLIKEENWHPIYDTLPQVTYHKISNDLLTKIKSHTPSPQHTCCMLCRRFLVEFGLL
KphV (KP84_0344)    SNLFLIKHKLVHATIPHLLRYNMTALSFADLFGLIKEENWHPIYDTLPQVTYHKISNDLLTKIKSHTPSPQHTCCMLCRRFLVEFGLL
BBaV (JKT9876)      PNLFLIEHKLVTETIPHLLRYNMTALSFADLFGLIKDENWHPIFDTLPQVTYHKISNDLLTKIKSHTPSPQHTCCMLCRRFLAEFGLL
BBaV (JKT9891)      PNLFLIEHKLVTETIPHLLRYNMTALSFADLFGLIKDENWHPIFDTLPQVTYHKISNDLLTKIKSHTPSPQHTCCMLCRRFLAEFGLL
BBaV (JKT9853)      PNLFLIEHKLVTETIPHLLRYNMTALSFADLFGLIKDENWHPIFDTLPQVTYHKISNDLLTKIKSHTPSPQHTCCMLCRRFLAEFGLL
BBaV (JKT7774)      PNLFLIEHKLVTETIPHLLRYNMTALSFADLFGLIKDENWHPIFDTLPQVTYHKISNDLLTKIKSHTPSPQHTCCMLCRRFLAEFGLL
KSaV (JKT10701)     PNLFLIEHKLVTETIPHLLRYNMTALSFADLFGLIKDENWHPIFDTLPQVTYHKINNDLLTKIKSHTPSPQHTCCMLCRRFLAEFGLL
NseV                SDLFLIEHQLETQPIPYLKRLDMTALSVADLYGVIKDENWHPIYDTLPQVKYHKLDQDLLKKIKEHTPCPQHTCCLLCRRFLAEFGLL
MenoV               IDLFLIKHTFMRQSINYLMRHNMTALSLADLYGVIKSENWKPLYDTLPQVTYHKISPTLLNNIQQHSSAPQHTCCMLCRRFLAEFGLL
                     :****:* :    * :* * :*****.***:*:**.***:*::******.**::.  ** :*:.*:..******:******.*****

HouV (16757)        LHKLNYKVFETTRAMLTHYDFVLTADNVDLNGILDFEDYKLRKCT-VAYDVKSQLRIMQPYYHALYSFYEHTGMYFISQPIYNSIVDP
HouV (V3872)        LHKLNYKVFETTRAMLTHYDFVLTADNVDLNGILDFEDYKLRKCT-VAYDVKSQLRIMQPYYHALYSFYEHTGMYFISQPIYNSIVDP
HouV (16740)        LHKLNYKVFETTRAMLTHYDFVLTADNVDLNGILDFEDYKLRKCT-VAYDVKSQLRIMQPYYHALYSFYEHTGMYFISQPIYNSIVDP
HouV (V3982)        LHKLNYKVFETTRAMLTHYDFVLTADNVDLNGILDFEDYKLRKCT-VAYDVKSQLRIMQPYYHALYSFYEHTGMYFISQPIYNSIVDP
NDiV                LHKLNYKVFETTRAMLTHYDFVLTADNVDLNGILDFEDYKLRKCT-VAYDVKSQLRIMQPYYHALYSFYEHTGMYFISQPIYNSIVDP
NgeV (JKT9982)      LHKLNYKVFETTRAMLTHYDFVLTADNVDLNGILDFEDYKLRKYT-VAYDVKSQLRIMQPYYHALYSFYEHTGMYFISQPIYNSIVDP
CavV                LHKLNYKVFETTRAILTHYDFVLTADNVDLNGILDFEDYKLKKST-IAHDVKSQLRIMQPYYHALYSFYEHTGMYFISQPIYNSIVDP
HanaV               LHKLNYKVFETTRKMLTHYDFVLTADNIDLNGILDFEDYVPKKYIDINIDVTSQLRIMQPYYHMLYSFYEHTGMYFISQPIYTSIVDP
KPhV (KP84_0156)    LHKLNYKVFQTTTHLLQGYEFVLTADNIDLNGILDFEDYRPREEY-FVCDVKHQLRIMQPYYHTLYSFYEHAGMYFISQPIYMSEVDP
KPhV (KP84_0192)    LHKLNYKVFQTTTHLLQGYEFVLTADNIDLNGILDFEDYRPREEY-FVCDVKHQLRIMQPYYHTLYSFYEHAGMYFISQPIYMSEVDP
KphV (KP84_0344)    LHKLNYKVFQTTTHLLQGYEFVLTADNIDLNGILDFEDYRPREEY-FVCDVKHQLRIMQPYYHTLYSFYEHAGMYFISQPIYMSEVDP
BBaV (JKT9876)      LHKLNKKVFETTNYILQRFEFVLTADNIDLNGILDFEDYIPRKEH-FEFDVKDQLRIMQPYYHTLYSFYEHTGMYFISQPIYTSEVDP
BBaV (JKT9891)      LHKLNKKVFETTNYILQRFEFVLTADNIDLNGILDFEDYIPRKEH-FEFDVKDQLRIMQPYYHTLYSFYEHTGMYFISQPIYTSEVDP
BBaV (JKT9853)      LHKLNKKVFETTNYILQRFEFVLTADNIDLNGILDFEDYIPRKEH-FEFDVKDQLRIMQPYYHTLYSFYEHTGMYFISQPIYTSEVDP
BBaV (JKT7774)      LHKLNKKVFETTNYILQRFEFVLTADNIDLNGILDFEDYIPRKEH-FEFDVKDQLRIMQPYYHTLYSFYEHTGMYFISQPIYMSEVDP
KSaV (JKT10701)     LHKLNKKVFETTTYILQRYEFVLTADNIDLNGILDFEDYMPREEY-FEFDVKDQLRIMQPYYHTLYSFYEHTGMYFISQPIYTSEVDP
NseV                MHKLNFKVYETTTTLLRSYEFVLTADNVDLNGILDFEDYLQLDEP-RNVECVDQLRKMQPYYHSLYSIYEHAGMYFISQPIYKHNVDS
MenoV               MHKLNKQVFSTINFMFQHYDFVLTSDNVDLNGILDFEDYTKTEYT-RTLELKDQLRKMQPYYHTLYSIYEHTGMYFISQPIYARPIDP
                    :**** :*:.*   ::  ::****:**:***********   .      :   *** ****** ***:***:**********   :*.

HouV (16757)        SLDLIQQFELAVEATRNLPLDAKFDDTPLYRPTIQHLAEYLKLNIYAMEPEPLWNCYDTMDCPQIELPGIDNAITSIITKPTRP-LSE
HouV (V3872)        SLDLIQQFELAVEATRNLPLDAKFDDTPLYRPTIQHLAEYLKLNIYAMEPEPLWNCYDTMDCPQIELPGIDNAITSIITKPTRP-LSE
HouV (16740)        SLDLIQQFELAVEATRNLPLDAKFDDTPLYRPTIQHLAEYLKLNIYAMEPEPLWNCYDTMDCPQIELPGIDNAITSIITKPTRP-LSE
HouV (V3982)        SLDLIQQFELAVEATRNLPLDAKFDDTPLYRPTIQHLAEYLKLNIYAMEPEPLWNCYDTMDCPQIELPGIDNAITSIITKPTRP-LSE
NDiV                SLDLIQQFESAVEATRNLPLDAKFDDTPLYRPTIQHLAEYLKLNIYAMEPEPLWNCYDTMDCPQIELPGIDNAITSIITKPTRP-LSE
NgeV (JKT9982)      SLDLIQQFELAVEATRNLPLDAKFDDTPLYRPTIQHLAEYLKLNIYAMEPEPLWNCYDTMDCPQIELPGIDQRYYKHNYKTNKTSLGK
CavV                SLDLIQQFELAVEAIRNLPLDVKFDDTPLYRPTIQHLTEYLKLNIYAMEPEPLWNCYDTMNCPQIELPEIDNAITSIITKPTRP-LSE
HanaV               NLDLIQQFESAIEVTRNLPLDGTFDEKPLYRPTIQHFTQYLKLNLYAMEPEPLWNCYDTMDSPLIEINGIDTTVTNIIIKPTRP-IPD
KPhV (KP84_0156)    SLDLIHQFELAVEAIRDLPLDSVFDDRPLFRPTIQHLTQYLKLNLYAMEPEPLWNCYDTMHCPPIELDGIDNSTTNIIVKPTRP-IPE
KPhV (KP84_0192)    SLDLIHQFELAVEAIRDLPLDSVFDDRPLFRPTIQHLTQYLKLNLYAMEPEPLWNCYDTMHCPPIELDGIDNSTTNIIVKPTRP-IPE
KphV (KP84_0344)    SLDLIHQFELAVEAIRDLPLDSVFDDRPLFRPTIQHLTQYLKLNLYAMEPEPLWNCYDTMHCPPIELDGIDNSTTNIIVKPTRP-IPE
BBaV (JKT9876)      SLDLIHQFELAVEATRNLPLDSTFDDQPLFRPTIQHLTQYLKLNLYAMEPEPLWNCYDTMNCPQIELAGIDNAITSIIVKPTRP-IPD
BBaV (JKT9891)      SLDLIHQFELAVEATRNLPLDSTFDDQPLFRPTIQHLTQYLKLNLYAMEPEPLWNCYDTMNCPQIELAGIDNAITSIIVKPTRP-IPD
BBaV (JKT9853)      SLDLIHQFELAVEATRNLPLDSTFDDQPLFRPTIQHLTQYLKLNLYAMEPEPLWNCYDTMNCPQIELAGIDNAITSIIVKPTRP-IPD
BBaV (JKT7774)      SLDLIHQFELAVEATRNLPLDSTFDDQPLFRPTIQHLTQYLKLNLYAMEPEPLWNCYDTMNCPQIELAGIDNAITSIIVKPTRP-IPD
KSaV (JKT10701)     SLDLIHQFELALEATRNLPLDSKFDDQPLFRPTIQHLTQYLKLNLYAMEPEPLWNCYDTMDCPQIKLDGIDNAITSIIVKPTRP-IPD
NseV                SVDLIQQFESVLHSVRNLPLDASFDDTPLYRPTIQHLTQYLKLNIYAMDPEPLWNSYDTMQCPQLEIPGIDNTVTNIIVKPTRP-IPE
MenoV               SIDLIDQFEMAISNIREFPLDGTFDDSTLYRPSIQHFAEYLKLDVYAVKPEPLWNCYDSMQAPQITIP--EHDSIQVILKPTRP-MSE
                    .:***.*** .:   *::***  **: .*:**:***:::****::**:.******.**:*..* : :   :    .   *..:. : .

HouV (16757)        YIELNHTTVKNFDGAIYCKVNHNEINNLQDILYCMPTDATIHELYIVDHPYELESHNRMLRTSLNIWLHNLYDANVNLSHFDSINYDK
HouV (V3872)        YIELNHTTVKNFDGDIYCKVNHNEINNLQDILYCMPTDATIHELYIVDHPYELESHNRMLRTSLNIWLHNLYDANVNLSHFDSINYDK
HouV (16740)        YIELNHTTVKNFDGDIYCKVNHNEINNLQDILYCMPTDATIHELYIVDHPYELESHNRMLRTSLNIWLHNLYDANVNLSHFDSINYDK
HouV (V3982)        YIELNHTTVKNFDGDIYCKVNHNEINNLQDILYCMPTDATIHELYIVDHPYELESHNRMLRTSLNIWLHNLYDANVNLSHFDSINYDK
NDiV                YIELNHTTVKNFDGDIYCKVNHNEINNLQDILYCMPTDATIHELYIVDHPYELESHNRMLRTSLNIWLHNLYDANVNLSHFDSINYDK
NgeV (JKT9982)      HRNLNHTTVKNFDGAIYCKVNHNEINNLQDILYCMPTDATIHELYIVDHPYELESHNRMLRTSLNIWLHNLYDANVNLSHFDSINYDK
CavV                YIELNHTTVKNLDGDVYCKIHHNEINNLQDILYSKPTDVTIHELYIVDHPYELESHNRMLRTSLNIWLHNLYDANVNLSHFDSINYDK
HanaV               YIELNYDTVKLLDGDVYCKVNHSEITKLQDILYCLPASALIHELYIEDHPYELESHNHMLRTSLNIWLHNLYDANVNLSHFDSINYDK
KPhV (KP84_0156)    YIELNHNTIKTFDGDVYCKLNYNEINNLQDILYSLPQNATILELYIVDHPYELESHNRMLRTSLNIWLHNLYDANVNLSHFDSINYEV
KPhV (KP84_0192)    YIELNHNTIKTFDGDVYCKLNYNEINNLQDILYSLPQNATILELYIVDHPYELESHNRMLRTSLNIWLHNLYDANVNLSHFDSINYEV
KphV (KP84_0344)    YIELNHNTIKTFDGDVYCKLNYNEINNLQDILYSLPQNATILELYIVDHPYELESHNRMLRTSLNIWLHNLYDANVNLSHFDSINYEV
BBaV (JKT9876)      YIELNHDTVKNFDGDTYCKVNYNDINNLQDILYCLPPNATILELYIVDHPYELESHNRMLRTSLNIWLHNLYDANVNLAHFDSINYEN
```

```
BBaV (JKT9891)     YIELNHDTVKNFDGDTYCKVNYNDINNLQDILYCLPPNATILELYIVDHPYELESHNRMLRTSLNIWLHNLYDANVNLAHFDSINYEN
BBaV (JKT9853)     YIELNHDTVKNFDGDTYCKVNYNDINNLQDILYCLPPNATILELYIVDHPYELESHNRMLRTSLNIWLHNLYDANVNLAHFDSINYEN
BBaV (JKT7774)     YIELNHDTVKNFDGDTYCKVNYNDINNLQDILYCLPPNATILELYIVDHPYELESHNRMLRTSLNIWLHNLYDANVNLAHFDSINYEN
KSaV (JKT10701)    YIELNHDTVKDFDGDIHCKINYNNINSLQDILYSLPLTATILELYIVDHPYELESHNRMLRTSLNIWLHNLYDANVNLAHFDSINFET
NseV               YIELTHSNVKNFNGPTYCFVNHSAINNIRDILNAMPQDATIHELYIVDHPYELESHNRMLRTSLNIWLHNLYDANVNLSHFDSINYEK
MenoV              YIELTHNTIKTFDGTIFCKIDHSQFNQLRDILCNLPNDAVIHELFIENHPYELEAYTRMLKVNLNIWLNNLYDANVNLAHFDSINYEH
                   : :*.: .:* ::*  .* :.:. :..::***    *   . * **:* :******::.:**:..*****:*********:******::
HouV (16757)       TRKASFPIVGTVPAITLRDCEICQDEIPDDLKDVYDFGSCVHAKAQLSDYTTPRKLNPLIEFDPALLRHGEFLPNNDYAYTMKTKPDH
HouV (V3872)       TRKASFPIVGTVPAITLRDCEICQDEIPDDLKDVYDFGSCVHAKAQLSDYTTPRKLNPLIEFDPALLRHGEFLPNNDYAYTMKTKPDH
HouV (16740)       TRKASFPIVGTVPAITLRDCEICQDEIPDDLKDVYDFGSCVHAKAQLSDYTTPRKLNPLIEFDPALLRHGEFLPNNDYAYTMKTKPDH
HouV (V3982)       TRKASFPIVGTVPAITLRDCEICQDEIPDDLKDVYDFGSCVHAKAQLSDYTTPRKLNPLIEFDPALLRHGEFLPNNDYAYTMKTKPDH
NDiV               TRKASFPIVGTVPAITLRDCEICQDEIPDDLKDVYDFGSCVHAKAQLSDYTTPRKLNPLIEFDPALLRHGEFLPNNDYAYTMKTKPDH
NgeV (JKT9982)     TRKASFPIVGTVPAITLRDCEICQDEIPDDLKDVYDFGSCVHAKAQLSDYTTPRKLNPLIEFDPALLRHGEFLPNNDYAYTMKTKPDH
CavV               TRKASFPIVGTVPAIPLRDCEVCQDEIPDDLKDVYDFGSCVHARAQLSDYTTPRKLNPLIEFDPALLRHGEFLPNNDYAYTMKTKPDH
HanaV              THKASFPIVGTVPAIPLRDCVHCQDDIPEDLKDVYDFGSCVHAKAQLSDYKLPRKIHPLIEFDTALLHIGEFQPNNDYAYTMKTKPDH
KPhV (KP84_0156)   SQKASFPIVGTVPAILLRDCEFCKDKIPDDLKDVYDFGSCVHAKAQLSDYKTPRKLHPLIEFDPALLRHGEFLPTNDYAYTMKTKPDH
KPhV (KP84_0192)   SQKASFPIVGTVPAILLRDCEFCKDKIPDDLKDVYDFGSCVHAKAQLSDYKTPRKLHPLIEFDPALLRHGEFLPTNDYAYTMKTKPDH
KphV (KP84_0344)   SQKASFPIVGTVPAILLRDCEFCKDKIPDDLKDVYDFGSCVHAKAQLSDYKTPRKLHPLIEFDPALLRHGEFLPTNDYAYTMKTKPDH
BBaV (JKT9876)     SQKATFPIVGTVPAILLRDCEICKDKIPDDLKDVYDFGSCVHAKAQLSDYKTPRKIHPLIEFDTALLRHGEFQPNNDYAYTMKTKPDH
BBaV (JKT9891)     SQKATFPIVGTVPAILLRDCEICKDKIPDDLKDVYDFGSCVHAKAQLSDYKTPRKIHPLIEFDTALLRHGEFQPNNDYAYTMKTKPDH
BBaV (JKT9853)     SQKATFPIVGTVPAILLRDCEICKDKIPDDLKDVYDFGSCVHAKAQLSDYKTPRKIHPLIEFDTALLRHGEFQPNNDYAYTMKTKPDH
BBaV (JKT7774)     SQKATFPIVGTVPAILLRDCEICKDKIPDDLKDVYDFGSCVHAKAQLSDYKTPRKIHPLIEFDTALLRHGEFQPNNDYAYTMKTKPDH
KSaV (JKT10701)    SQKATFPIVGTVPAILLRDCEICKDKIPDNLKDVYDFGSCVHAKAQLSDYKTPRKIHPLIEFDTALLRHGEFQPNNDYAYTMKTKPDH
NseV               THQANFPIVGSVPAVPLLDCELCKDKITDDIKAVYDIGSCIHAKAQLEDYQLPRKVNPLVEFDTALLKIGTFRPGSDYTYTMKTKPDL
MenoV              TKTISFPIIGKMPATLLADCEQCQDEIPNDIKAVYDLGSNVHAKANLQDYKTPRKINPLVEFDTALLKHGDFNEHNEYKYTMKTKPDL
                   ::   .***:*.:**   * **   *:*.*.:::* ***:** :**:*:*.**   ***::**:***.***: * *    .:* ********

HouV (16757)       LIDRELKDYIDSTGLTALIPPLDINPAVHDPETTYSSPYYIKTPSEPSIRQDLELFNQNTAGSVSPTVFLMAIELLHQLLTEEISASD
HouV (V3872)       LIDRELKDYIDSTGLTALIPPLDINPAVHDPETTYSSPYYIKTPSEPSIRQDLELFNQNTAGSVSPTVFLMAIELLHQLLTEEISASD
HouV (16740)       LIDRELKDYIDSTGLTALIPPLDINPAVHDPETTYSSPYYIKTPSEPSIRQDLELFNQNTAGSVSPTVFLMAIELLHQLLTEEISASD
HouV (V3982)       LIDRELKDYIDSTGLTALIPPLDINPAVHDPETTYSSPYYIKTPSEPSIRQDLELFNQNTAGSVSPTVFLMAIELLHQLLTEEISASD
NDiV               LIDRELKDYIDSTGLTALIPPLDINPAVHDPETTYSSSYYIKTPSETSIRQDLELFNQNTAGSVSPTVFLMAIELLHQLLTEEISASD
NgeV (JKT9982)     LIDRELKDYIDSTGLTALIPPLDINPAVHDPETTYSSSYYIKTPSEPSIRQDLELFNQNTAGSVSPTVFLMAIELLHQLLTEEISASD
CavV               LIDCELKDYIDSTGLTALIPPLDINPAVHDPETTYSSSYYIKTPSETSIRQDLELFNQNTAGSVSPTVFLMAIELLHQLLTEEISASD
HanaV              LIDWELKEYLDSTGLTALIPPLNVNPAVHDPETTFSSPYYIKTPSEESIRQDLELFNQNTAGSVSPTVFLMAIELLHQLLTEEVSASD
KPhV (KP84_0156)   IIDLELKEYIDSTGLTALIPPLNINPSVHDPETTFSSPYYIKTPSEVSIRADLELFNQNTAGSVSPTVFLMAIELLHQLLTEEISASD
KPhV (KP84_0192)   IIDLELKEYIDSTGLTALIPPLNINPSVHDPETTFSSPYYIKTPSEVSIRADLELFNQNTAGSVSPTVFLMAIELLHQLLTEEISASD
KphV (KP84_0344)   IIDLELKEYIDSTGLTALIPPLNINPSVHDPETTFSSPYYIKTPSEVSIRADLELFNQNTAGSVSPTVFLMAIELLHQLLTEEISASD
BBaV (JKT9876)     IVDLELKEYIDSTGLKALIPPLNINPAVHDPETTYESPYYIKTPSEQSIRADLELFNQNTAGSVSPTVFLMAIELLHQLLTEEISASD
BBaV (JKT9891)     IVDLELKEYIDSTGLKALIPPLNINPAVHDPETTYESPYYIKTPSEQSIRADLELFNQNTAGSVSPTVFLMAIELLHQLLTEEISASD
BBaV (JKT9853)     IVDLELKEYIDSTGLKALIPPLNINPAVHDPETTYESPYYIKTPSEQSIRADLELFNQNTAGSVSPTVFLMAIELLHQLLTEEISASD
BBaV (JKT7774)     IVDLELKEYIDSTGLKALIPPLNINPAVHDPETTYESPYYIKTPSEQSIRADLELFNQNTAGSVSPTVFLMAIELLHQLLTEEISASD
KSaV (JKT10701)    IIDLELKEYIDSTGLKALIPPLNINPAVHDPETTYSSPYYIKTPSEQSIRADLELFNQNTAGSVSPTVFLMAIELLHQLLTEEISASD
NseV               LVDSELKEYIDNSGLTALIPPLNPNPSVHDPATTFTSPYYIKTPSEASIREDLEMFNQNTAGSVSPTVFLMAVELLKQLLTEEILASD
MenoV              YIDYELKSHLDSTGITALIPPLNINPIVNDPNLTFTSPYYIKTPSADSIKEDLEMFNQNTAAAVSPVVYLMATELLHQLLTETILASD
                    :* ***.::*.:*:.******: ** *:**   *: *.*******   **: ***:******.:***.*:*** ***:***** : ***

HouV (16757)       GKPNCPMVPSEVPVRNKHKSAGTPYRKFGDSEFMRELYGNYRDAIVYHKRHSADQQLTLTINKVAPSKNHRDRTILAISINKSEPGRS
HouV (V3872)       GKPNCPMVPSEVPVRNKHKSAGTPYRKFGDSEFMRELYGNYRDAIVYHKRHSADQQLTLTINKVAPSKNHRDRTILAISINKSEPGRS
HouV (16740)       GKPNCPMVPSEVPVRNKHKSAGTPYRKFGDSEFMRELYGNYRDAIVYHKRHSADQQLTLTINKVAPSKNHRDRTILAISINKSEPGRS
HouV (V3982)       GKPNCPMVPSEVPVRNKHKSAGTPYRKFGDSEFMRELYGNYRDAIVYHKRHSADQQLTLTINKVAPSKNHRDRTILAISINKSEPGRS
NDiV               GKPNCPMVPSEVPVRNKHKSAGTPYRKFGDSEFMRELYGNYRDAIVYHKRHSADQQLTLTINKVAPSKNHRDRTILAISINKSEPGRS
NgeV (JKT9982)     GKPNCPMVPSEVPVRNKHKSAGTPYRKFGDSEFMRELYGNYRDAIVYHKRHSADQQLTLTINKVAPSKNHRDRTILAISINKSEPGRS
CavV               GKPNCPMVPSEVPVRNKHKSAGTPYRKFGDSEFMRELYGNYRDAIVYHKRHSADQQLTLTINKVAPSKNHRDRTILAISINKSEPGRS
HanaV              GKPNCPMVPSEVPVRNKHKSAGTPYRKFGDSEFMRELLGDYRDAIVHHKRHSADQHLTLTINKVATSTKHRDRTILAISINKSEAGRS
KPhV (KP84_0156)   GKPNCPMVPSEVPVRNKHKSAGTPYRKFGDSEFMRELYGKYRDAIVYHKRHSADQHLTLTINKVATSKNHRDRTILAISINKSEPGRS
KPhV (KP84_0192)   GKPNCPMVPSEVPVRNKHKSAGTPYRKFGDSEFMRELYGKYRDAIVYHKRHSADQHLTLTINKVATSKNHRDRTILAISINKSEPGRS
KphV (KP84_0344)   GKPNCPMVPSEVPVRNKHKSAGTPYRKFGDSEFMRELYGKYRDAIVYHKRHSADQHLTLTINKVATSKNHRDRTILAISINKSEPGRS
BBaV (JKT9876)     GKPNCPMVPSEVPVRNKHKSAGTPYRKFGDSEFMRELYGKYRDAIVYHKRHSADQHLTLTINKVATSKNHRDRTILAISINKSEPGRS
BBaV (JKT9891)     GKPNCPMVPSEVPVRNKHKSAGTPYRKFGDSEFMRELYGKYRDAIVYHKRHSADQHLTLTINKVATSKNHRDRTILAISINKSEPGRS
BBaV (JKT9853)     GKPNCPMVPSEVPVRNKHKSAGTPYRKFGDSEFMRELYGKYRDAIVYHKRHSADQHLTLTINKVATSKNHRDRTILAISINKSEPGRS
BBaV (JKT7774)     GKPNCPMVPSEVPVRNKHKSAGTPYRKFGDSEFMRELYGKYRDAIVYHKRHSADQHLTLTINKVATSKNHRDRTILAISINKSEPGRS
KSaV (JKT10701)    GKPNCPMVPSEVPVRNKHKSAGTPYRKFGDSEFMRELYGKYRDAIVYHKRHSADQHLTLTINKVATSKNHRDRTILAISINKSEPGRS
NseV               GLPNCPMVPSEVPVRHKHKSSGTPYRGHGDAEFMRQLYGSARDDLVYHKRHSADQMLTLVINKVANSTKHRDRTILAININKSEPGRA
MenoV              GKPNCPMVPSEVPIRNKHKSSGTPYRTFGDSEFMRTLYGEYRDKLVYHKRHSADQALTLVINKVAISTKHRDRTILAININKSEAGRS
                   * ***********:*:****:*****  .**:**** * *. ** :*:********* ***.***** *.:*********.*****.**:

HouV (16757)       LYRWNLDKIKYTSSLGGPILIGFTAQYGGWDKLYKYLYKNSPADNPDTAEHAVLGGKDYPKWDRRISNMLQLTTTTVLYSLIDPNTQR
HouV (V3872)       LYRWNLDKIKYTSSLGGPILIGFTAQYGGWDKLYKYLYKNSPADNPDTAEHAVLGGKDYPKWDRRISNMLQLTTTTVLYSLIDPNTQR
HouV (16740)       LYRWNLDKIKYTSSLGGPILIGFTAQYGGWDKLYKYLYKNSPADNPDTAEHAVLGGKDYPKWDRRISNMLQLTTTTVLYSLIDPNTQR
HouV (V3982)       LYRWNLDKIKYTSSLGGPILIGFTAQYGGWDKLYKYLYKNSPADNPDTAEHAVLGGKDYPKWDRRISNMLQLTTTTVLYSLIDPNTQR
NDiV               LYRWNLDKIKYTSSLGGPILIGFTAQYGGWDKLYKYLYKNSPADNPDTAEHAVLGGKDYPKWDRRISNMLQLTTTTVLYSLIDPNTQR
NgeV (JKT9982)     LYRWNLDKIKYTSSLGGPILIGFTAQYGGWDKLYKYLYKNSPADNPDTAEHAVLGGKDYPKWDRRISNMLQLTTTTVLYSLIDPNTQR
CavV               LYRWNLDKIKYTSSLGGPILIGFTAQYGGWDKLYKYLYKNSPADHPDIAEHAVLGGKDYPKWDRRISNMLQLTTTTVLYSLIDPNTQS
HanaV              LYRWNLDKIKYTASLGGPILIGFTAQYGGWDKFYKQLYKNSPADQPGVAERAVLGGKDYPKWDRRISNMLQLTTTTILYSLIDPNTQK
KPhV (KP84_0156)   LYRWNLDKIKYTASLGGPILIGFTAQYGGWDRLYKYLYKDSPADNPATAEYAVLGGKDYPKWDRRISNMLQLTTTTVLYSLIDPNIQE
KPhV (KP84_0192)   LYRWNLDKIKYTASLGGPILIGFTAQYGGWDRLYKYLYKDSPADNPATAEYAVLGGKDYPKWDRRISNMLQLTTTTVLYSLIDPNIQE
KphV (KP84_0344)   LYRWNLDKIKYTASLGGPILIGFTAQYGGWDRLYKYLYKDSPADNPATAEYAVLGGKDYPKWDRRISNMLQLTTTTVLYSLIDPNIQE
BBaV (JKT9876)     LYRWNLDKIKHTASLGGPILIGFTAQYGGWDKLYKYLYKDSPADNPATAEHAVLGGKDYPKWDRRISNMLQLTTTTVLYSLIDPNTQT
```

```
BBaV (JKT9891)     LYRWNLDKIKHTASLGGPILIGFTAQYGGWDKLYKYLYKDSPADNPATAEHAVLGGKDYPKWDRRISNMLQLTTTTVLYSLIDPNTQT
BBaV (JKT9853)     LYRWNLDKIKHTASLGGPILIGFTAQYGGWDKLYKYLYKDSPADNPATAEHAVLGGKDYPKWDRRISNMLQLTTTTVLYSLIDPNTQT
BBaV (JKT7774)     LYRWNLDKIKHTASLGGPILIGFTAQYGGWDKLYKYLYKDSPADNPATAEHAVLGGKDYPKWDRRISNMLQLTTTTVLYSLIDPNTQT
KSaV (JKT10701)    LYRWNLDKIKYTASLGGPILIGFTAQYGGWDKLYKYLYKDSPADNPATAEYAVLGGKDYPKWDRRISNMLQLTTTTVLYSLIDPNTQT
NseV               LYRWHLDKIKYTANNGGPILIGFIAQYGGWDKFYKQLYKNSPSDNPETAQYAVLGGKDYPKWDRRISNMLQLTTTNVLFSLIDQTTQL
MenoV              LFRWFLDKIKDTANKGGPILIGLIAQYGGWDKLYKNLYKDSPIDNPDATEHVVLGGKDYPKWDRRISNMLQLTTTNVFFNLIDQNTQF
                   *:** ***** *:. *******: *******::** ***:** *:*  :: .***********************.:::.*** . *
HouV (16757)       KLNNATPAQTWHEYMAETTQVLYDYLVFGNELYQKPGGVTSGNSRTADGNSLLHLLIDFYAIISQLIQSTPENVHLEVNLRNALCKTV
HouV (V3872)       KLNNATPAQTWHEYMAETTQVLYDYLVFGNELYQKPGGVTSGNSRTADGNSLLHLLIDFYAIISQLIQSTPENVHLEVNLRNALCKTV
HouV (16740)       KLNNATPAQTWHEYMAETTQVLYDYLVFGNELYQKPGGVTSGNSRTADGNSLLHLLIDFYAIISQLIQSTPENVHLEVNLRNALCKTV
HouV (V3982)       KLNNATPAQTWHEYMAETTQVLYDYLVFGNELYQKPGGVTSGNSRTADGNSLLHLLIDFYAIISQLIQSTPENVHLEVNLRNALCKTV
NDiV               KLNNATPAQTWHEYMAETTQVLYDYLVFGNELYQKPGGVTSGNSRTADGNSLLHLLIDFYAIISQLIQSTPENVHLEVNLRNALCKTV
NgeV (JKT9982)     KLNNATPAQTWHEYMAETTQVLYDYLVFGNELYQKPGGVTSGNSRTADGNSLLHLLIDFYAIISQLIQSTPENVHLEVNLRNALCKTV
CavV               KLNNATPAQTWHEYMAETTQVLFDYLVFGNELYQKPGGVTSGNSRTADGNSLLHLLIDFYAIISQLIQSTPENVHLEVNLRNALCKTV
HanaV              KLNNANPAQTWHEYMAETTQVLFDYLVFGNELYQKPGGVTSGNSRTADGNSLLHLLIDFYAIITQLIQSKPHNVHLHSKLRNRLCKTV
KPhV (KP84_0156)   KLNNATPSQTWHEYMAETTQVLFDYLVFGNELFQKPGGVTSGNSRTADGNSLLHLLIDFYAIIIQLIQSTPENVNLHPKLRNSLCKTV
KPhV (KP84_0192)   KLNNATPSQTWHEYMAETTQVLFDYLVFGNELFQKPGGVTSGNSRTADGNSLLHLLIDFYAIIIQLIQSTPENVNLHPKLRNSLCKTV
KphV (KP84_0344)   KLNNATPSQTWHEYMAETTQVLFDYLVFGNELFQKPGGVTSGNSRTADGNSLLHLLIDFYAIIIQLIQSTPENVNLHPKLRNSLCKTV
BBaV (JKT9876)     KLNNATPSQTWHEYMAETTQVLFDYLVFGNELFQKPGGVTSGNSRTADGNSLLHLLIDFYAIIIQLIQSVPENVHLHAKLRNKLCKLV
BBaV (JKT9891)     KLNNATPSQTWHEYMAETTQVLFDYLVFGNELFQKPGGVTSGNSRTADGNSLLHLLIDFYAIIIQLIQSVPENVHLHAKLRNKLCKLV
BBaV (JKT9853)     KLNNATPSQTWHEYMAETTQVLFDYLVFGNELFQKPGGVTSGNSRTADGNSLLHLLIDFYAIIIQLIQSVPENVHLHAKLRNKLCKLV
BBaV (JKT7774)     KLNNATPSQTWHEYMAETTQVLFDYLVFGNELFQKPGGVTSGNSRTADGNSLLHLLIDFYAIIIQLIQSVPENVHLHAKLRNKLCKLV
KSaV (JKT10701)    KLNDATPSQTWHEYMAETTQVLFDYLVFGSELLQKPGGVTSGNSRTADGNSLLHLLIDFYATIIQLIQSTPENVNLHAKLRNKLCKLV
NseV               RRNNANPAETWHEYMAETTQVLFDYLVFGNELHQKPGGVTSGNSRTADGNSLLHLLIDFYAIIIQLIQSTPENVDLQSELRSRLCKIV
MenoV              KNNQASPSETWHEYMSETTQILFDYLVFGNELYQKPGGVTSGNSRTADGNSFLHMLIDMYALIIQLIQSTPENVHIESKLRNTLCKTV
                   : *:*.*::******:****:*:******.** *****************:**:***:** * ***** *.**.:. :**. *** *

HouV (16757)       FTRIPSDYIDSS-CVTLRNTDTLHIIRRRVAKGAYLSDDGLIVIDPRIIRYDDFMSVSHLISHYMIAQNKHKYHIDAIQRYAREFLSQ
HouV (V3872)       FTRIPSDYIDSS-CVTLRNTDTLHIIRRRVAKGAYLSDDGLIVIDPRIIRYDDFMSVSHLISHYMIAQNKHKYHIDAIQRYAREFLSQ
HouV (16740)       FTRIPSDYIDSS-CVTLRNTDTLHTIRRRVAKGAYLSDDGLIVIDPRIIRYDDFMSVSHLISHYMIAQNKHKYHIDAIQRYAREFLSQ
HouV (V3982)       FTRIPSDYIDSS-CVTLRNTDTLHTIRRRVAKGAYLSDDGLIVIDPRIIRYDDFMSVSHLISHYMIAQNKHKYHIDAIQRYAREFLSQ
NDiV               FTRIPSDYIDSS-CVTLRNTDTLHTIRRRVAKGAYLSDDGLIVIDPRIIRYDDFMSVSHLISHYMIAQNKHKYHIDAIQRYAREFLSQ
NgeV (JKT9982)     FTRIPSDYIDSS-CVTLRNTDTLHTIRRRVAKGAYLSDDGLIVIDPRIIRYDDFMSVSHLISHYMIAQNKHKYHIDAIQRYAREFLSQ
CavV               FTKIPSDYIDSS-CVTLRNTDILHTIRRRVAKGAYLSDDGLIVIDPRIIRYDDFMSVSHLISHYMIAQNKHKYHIDAIQRYAREFLSQ
HanaV              FTKIPADYINTS-CVTLRKTDTLRTIRLKIAKNLVLSDDGLLVLDPEIIDYTDYMSISHLISHYMMAQNKHKYHIDAISSYAREFLSQ
KPhV (KP84_0156)   FTMIPADYIDQS-CVTLRKTDTLRIIRQSIAKGTYLSDDGLLVHDPRIIRYDDFMSISHLISHYMIAQNKHKYHVDAIKRFAREFLSQ
KPhV (KP84_0192)   FTMIPADYIDQS-CVTLRKTDTLRIIRQSIAKGTYLSDDGLLVHDPRIIRYDDFMSISHLISHYMIAQNKHKYHVDAIKRFAREFLSQ
KphV (KP84_0344)   FTMIPADYIDQS-CVTLRKTDTLRIIRQSIAKGTYLSDDGLLVHDPRIIRYDDFMSISHLISHYMIAQNKHKYHVDAIKRFAREFLSQ
BBaV (JKT9876)     FTTIPSDYIDQS-SVTLRKTDILHIIRLCIAKGTYLSDDGLLVHDPRIIRYDDFMAISHLISHYMIALNKHKYHVDAILRYAREFLSQ
BBaV (JKT9891)     FTTIPSDYIDQS-SVTLRKTDILHIIRLCIAKGTYLSDDGLLVHDPRIIRYDDFMAISHLISHYMIALNKHKYHVDAILRYAREFLSQ
BBaV (JKT9853)     FTTIPSDYIDQS-SVTLRKTDILHIIRLCIAKGTYLSDDGLLVHDPRIIRYDDFMAISHLISHYMIALNKHKYHVDAILRYAREFLSQ
BBaV (JKT7774)     FTTIPSDYIDQS-SVTLRKTDILHIIRLCIAKGTYLSDDGLLVHDPRIIRYDDFMAISHLISHYMIALNKHKYHVDAILRYAREFLSQ
KSaV (JKT10701)    FTTIPSDYIDQS-SVTLRNTDILHTIRLTVAKGTYLSDDGLFVHDPRIIIYDDFMSISHLISHYMISLNKHKYHVDAIQRFAREFLSQ
NseV               FTKIPADYIDDLNTDRLVKTDVLKTIRLNVAKGLYLSDDGLLVFDPRIIRYDDFMAVSHMISYYMIAQNKHKYHIDAIERYAREFLSQ
MenoV              FETIPSDYIDNTNVNLLRNTDILHRIRTRIAKGAYLSDDGLILIDTRLVKYDDFMAESHMISKYAIATNKHKYHLDPVQRKAREFLSQ
                   *  **:***:      * :** *: **  :**.  ******:: *..:: * *:*: **:** * :: ******:*.:   *******

HouV (16757)       DTIKFGDMVYPIPEFGRMYTAMLLSDNKNTLDPQINITRLLALFSYLYIYYFKYADQPTHPTLKFLDALRTYIENKLNTTDEIFLDCI
HouV (V3872)       DTIKFGDMVYPIPEFGRMYTAMLLSDNKNTLDPQINITRLLALFSYLYIYYFKYEDQPTHPTLKFLDALRTYIENKLNTTDEIFLDCI
HouV (16740)       DTIKFGDMVYPIPEFGRMYTAMLLSDNKNTLDPQINITRLLALFSYLYIYYFKYEDQPTHPTLKFLDALRTYIENKLNTTDEIFLDCI
HouV (V3982)       DTIKFGDMVYPIPEFGRMYTAMLLSDNKNTLDPQINITRLLALFSYLYIYYFKYEDQPTHPTLKFLDALRTYIENKLNTTDEIFLDCI
NDiV               DTIKFGDMVYPIPEFGRMYTAMLLSDNKNTLDPQINITRLLALFSYLYIYYFKYEDQPTHPILKFLDALRTYIENKLNTTDEIFLDCI
NgeV (JKT9982)     DTIKFGDMVYPIPEFGRMYTAMLLSDNKNTLDPQINITRLLALFSYLYIYYFKYEDQPTHPTLKFLDALRTYIENKLNTTDEIFLDCI
CavV               DTIKFGDMVYPIPEFGRMYTAMLLSDNKNTLDPQINITRLLALFSYLYIYYFKYVDQPTHPILKFLDALRTYIELKLNTTDEIFLDCI
HanaV              GPHKFGDMVFPIPEFGRMYSAMLLSDNKNILDPKINITRLLALFSYLYIYYFKYEDEPTHPIVKFLDALRTYIDNKLDITDEIFLDCI
KPhV (KP84_0156)   DTIQFGDMAYPIPEFGRMYTAMLLSDNKNTLDPQINITRLLALFSYLYIYYFKYEDQPTHPIIKFLDALRTYIENKLNTTDEIFLDCV
KPhV (KP84_0192)   DTIQFGDMAYPIPEFGRMYTAMLLSDNKNTLDPQINITRLLALFSYLYIYYFKYEDQPTHPIIKFLDALRTYIENKLNTTDEIFLDCV
KphV (KP84_0344)   DTIQFGDMAYPIPEFGRMYTAMLLSDNKNTLDPQINITRLLALFSYLYIYYFKYEDQPTHPIIKFLDALRTYIENKLNTTDEIFLDCV
BBaV (JKT9876)     DTIKFGDMVYPIPEFGRMYSAMFLSDNKNTLDPQINITRLLALFSYLYIYYFKFEDQPTHPILKFLDALRTYIENKLDTTDEIFLDRV
BBaV (JKT9891)     DTIKFGDMVYPIPEFGRMYSAMFLSDNKNTLDPQINITRLLALFSYLYIYYFKFEDQPTHPILKFLDALRTYIENKLDTTDEIFLDRV
BBaV (JKT9853)     DTIKFGDMVYPIPEFGRMYSAMFLSDNKNTLDPQINITRLLALFSYLYIYYFKFEDQPTHPILKFLDALRTYIENKLDTTDEIFLDRV
BBaV (JKT7774)     DTIKFGDMVYPIPEFGRMYSAMFLSDNKNTLDPQINITRLLALFSYLYIYYFKFEDQPTHPILKFLDALRTYIENKLDTTDEIFLDRV
KSaV (JKT10701)    DTIKFGDMAYPIPEFGRMYSAMLLSDNKNTLDPQINITRLLALFSYLYIYYFKFEDQPTHPILKFLDALRTYIENKLDTTDEIFLDCV
NseV               DTIKFGDMVYPIPEFGRIYSAMLLSDNKNTLDPQINTTRLLALFSYLYMYYFKFVDQPTEPTLKLLDIIRDYLEQKLDSTEEIFLDCV
MenoV              DTFHFGDMAFPIPEFGRIYSAMLLSDNRNTLEPSISITRLLALYSYLYIFYFKYANTPTHHELKLLDAIRTYIDDHLENTEDIFLDCV
                   .. :****.:*******:*:**:****:* *:*.*. ******:****::***: : **.  :*:** :* *:: :*: *::**** :

HouV (16757)       KVPDLQDVEFDLKNCDLYENFDYLWGLDQSSAYMDYLCKYKHRYRNLSLFKRQLIQHHEEAQ-LHNENKLMNKGKLITYNCYVCGENA
HouV (V3872)       KVPDLQDVEFDLKNCDLYENFDYLWGLDQSSAYMDYLCKYKHRYRNLSLFKRQLIQHHEEAQ-LHNENKLMNKGKLITYNCYVCGENA
HouV (16740)       KVPDLQDVEFDLKNCDLYENFDYLWGLDQSSAYMDYLCKYKHRYRNLSLFKRQLIQHHEEAQ-LHNENKLMNKGKLITYNCYVCGENA
HouV (V3982)       KVPDLQDVEFDLKNCDLYENFDYLWGLDQSSAYMDYLCKYKHRYRNLSLFKRQLIQHHEEAQ-LHNENKLMNKGKLITYNCYVCGENA
NDiV               KVPDLQDVEFDLKNCDLYENFDYLWGLDQSSAYMEYLCKYKHRYRNLSLFKRQLIQHHEEAQ-LHNENKLMNKGKLITYNCYVCGENA
NgeV (JKT9982)     KVPDLQDVEFDLKNCDLYENFDYLWGLDQSSAYMEYLCKYKHRYRNLSLFKRQLIQHHEEAR-LHNENKLMNKGKLITYNCYVCGENA
CavV               KVPDLQDVEFDLKNCDLYENFDYLWGLDQSSAYMDYLCKYKHRYRNLSLFKRQLIQHHEEAQ-LHNENKLKNKGRLITYNCYVCGENA
HanaV              KIPDLQDIEFDLKNCDLYENLDHLWGLDQSSAYMDYLCKYRHRYRNLSIFKRQLIQQHEQAQ-LHNETKLLNKGKLISYNCYVCGDNA
KPhV (KP84_0156)   KIPDLQDVEFDLKNCDLYENFDYLWGLDQSSAYMNYLCKYKHRYRNLSLFKRQLIQQHELTL-LHNPTTLVNKGKLITYNCYVCGENA
KPhV (KP84_0192)   KIPDLQDVEFDLKNCDLYENFDYLWGLDQSSAYMNYLCKYKHRYRNLSLFKRQLIQQHELTL-LHNPTTLVNKGKLITYNCYVCGENA
KphV (KP84_0344)   KIPDLQDVEFDLKNCDLYENFDYLWGLDQSSAYMNYLCKYKHRYRNLSLFKRQLIQQHELTL-LHNPTTLVNKGKLITYNCYVCGENA
BBaV (JKT9876)     KIPDLQDVEFDLKNCDLYENFDYLWGLDQSSAYMDYLCKYKHRYRNLSLFKRQLIQQHELDQ-LHNHTNLVNKGKLITYNCYVCGENA
```

```
BBaV (JKT9891)      KIPDLQDVEFDLKNCDLYENFDYLWGLDQSSAYMDYLCKYKHRYRNLSLFKRQLIQQHELDQ-LHNHTNLVNKGKLITYNCYVCGENA
BBaV (JKT9853)      KIPDLQDVEFDLKNCDLYENFDYLWGLDQSSAYMDYLCKYKHRYRNLSLFKRQLIQQHELDQ-LHNHTNLVNKGKLITYNCYVCGENA
BBaV (JKT7774)      KIPDLQDVEFDLKNCDLYENFDYLWGLDQSSAYMDYLCKYKHRYRNLSLFKRQLIQQHELDQ-LHNHTNLVNKGKLITYNCYVCGENA
KSaV (JKT10701)     KVPDLQDVEFDLKNCDLYENFDYLWGLDQSSAYMDYLCKYKHRYRNLSLFKRQLIQQHEQDQ-LHNHTNLVNKGKLITYNCYVCGENA
NseV                KIPDLQDVTFDLRNCDMYENFDHLWGLDLSSNYMEYLCKYQHRYRNLSLFKRQMIQQYEQDK-LNNENKLVNKGKLITYNCYVCGENA
MenoV               KIPELQDVQFDLKNCDMFENFDHLWGLDQSSAYIEYLHKYRYRYKNLSLFKVQLLQEYELQLNSHNKTELQNKGQLITYNCYVCGENA
                    *:*:***: ***:***::**:*:***** ** *::** **::**:***:** *::*.:*     :* . * ***:**:*******:**
HouV (16757)        YLTCATCERAFCNSADTNHGSHMEQHLQYSGHTCLYLNCKTVKCQHCFTTDINLLYTTGR-DHYCESHKPKNAVRILNYNDNTKLPPL
HouV (V3872)        YLTCATCERAFCNSADTNHGSHMEQHLQYSGHTCLYLNCKTVKCQHCFTTDINLLYTTGR-DHYCESHKPKNAVRILNYNDNTKLPPL
HouV (16740)        YLTCATCERAFCNSADTNHGSHMEQHLQYSGHTCLYLNCKTVKCQHCFTTDINLLYTTGR-DHYCESHKPKNAVRILNYNDNTKLPPR
HouV (V3982)        YLTCATCERAFCNSADTNHGSHMEQHLQYSGHTCLYLNCKTVKCQHCFTTDINLLYTTGR-DHYCESHKPKNAVRILNYNDNTKLPPL
NDiV                YLTCATCERAFCNSADTNHGSHMEQHLQYSGHTCLYLNCKTVKCQHCFTMDINLLYTTGR-DHYCESHKPKNAVRILNYNANTKLPPL
NgeV (JKT9982)      YLTCATCERAFCNSADTNHGSHMEQHLQYSGHTCLYLNCKTVKCQHCFTTDINLLYTTGR-DHYCESHKPKNAVRILNYNANTKLPPL
CavV                YLTCATCERAFCNSADTNHGSHMEQHLQYSGHTCLYLNCKTVKCHHCFTTDINLLYTTGR-DHYCEAHKPKNAVRILNHNDNTKLPPL
HanaV               YLTCATCERAFCNNTDTNHGSHIEQHLQYSGHTCLYLNCKTVKCQHCYTSDINLLYTTGR-EHFCEIHKPKNAVRILNSNDNEKLPPR
KPhV (KP84_0156)    YLTCATCERAFCNSADTDHGSHIEQHLQYSGHTCLYLNCKTVKCKHCFTNDINLLYTTGR-EHFCEAHRPKNAARILNSNVNPKLPPL
KPhV (KP84_0192)    YLTCATCERAFCNSADTDHGSHIEQHLQYSGHTCLYLNCKTVKCKHCFTNDINLLYTTGR-EHFCEAHRPKNAARILNSNVNPKLPPL
KphV (KP84_0344)    YLTCATCERAFCNSADTDHGSHIEQHLQYSGHTCLYLNCKTVKCKHCFTNDINLLYTTGR-EHFCEAHRPKNAARILNSNVNPKLPPL
BBaV (JKT9876)      YLTCATCERAFCNSADTNHGSHIEQHLQYSGHTCLYLNCKTVKCQHCFTNDINLLYTTGR-EHFCVSHKPKNAARILNSNANSKLPPL
BBaV (JKT9891)      YLTCATCERAFCNSADTNHGSHIEQHLQYSGHTCLYLNCKTVKCQHCFTNDINLLYTTGR-EHFCVSHKPKNAARILNSNANSKLPPL
BBaV (JKT9853)      YLTCATCERAFCNSADTNHGSHIEQHLQYSGHTCLYLNCKTVKCQHCFTNDINLLYTTGR-EHFCVSHKPKNAARILNSNANSKLPPL
BBaV (JKT7774)      YLTCATCERAFCNSADTNHGSHIEQHLQYSGHTCLYLNCKTVKCQHCFTNDINLLYTTGR-EHFCVSHKPKNAARILNSNANPKLPPL
KSaV (JKT10701)     YLTCATCERAFCNSADSDHGSHIEQHLQYSGHTCLYLNCKTVKCQHCFTNDINLLYTTGR-EHFCEAHKPKNAARILNSNANPKLPPL
NseV                YLTCATCERAFCNSSDSIHGSHLEQHLQYSEHNCLYLNCRTVKCSHCYTTDINVLYTSGK-NVFCEAHKPKNAACLIQNNVNPKSPPH
MenoV               YITCATCEKSFCNSKDTIHGSHIEQHLQYSGHDCLYLNCKTIKCRHCFAIDINKLYTSGKDMYFCETHKPKHAIRLVNNNVASNLPPL
                    *:******::***. *: ****:******* * ******:*:** **:: *** ***:*:   :*  *:**:*  ::: *   : **

HouV (16757)        LYLCVTDTKRVTFYEQCYINYTKAHPTYAISKEQFMSLIQLYLHQDYTLPVNQLANRIRVSLQLSSYGVVRPYHQLIMQLTKLESKVL
HouV (V3872)        LYLCVTDTKRVTFYEQCYINYTKAHPTYAISKEQFMSLIQLYLHQDYTLPVNQLANRIRVSLQLSSYGVVRPYHQLIMQLTKLESKVL
HouV (16740)        LYLCVTDTKRVTFYEQCYINYTKAHPTYAISKEQFMSLIQLYLHQDYTLPVNQLANRIRVSLQLSSYGVVRPYHQLIMQLTKLESKVL
HouV (V3982)        LYLCVTDTKRVTFYEQCYINYTKAHPTYAISKEQFMSLIQLYLHQDYTLPVNQLANRIRVSLQLSSYGVVRPYHQLIMQLTKLESKVL
NDiV                LYLCVTDTKRVTFYEQCYINYTKAHPTYAISKEQFMSLIQLYLHQDYTLPVNQLANRIRVSLQLSSYGVVRPYHQLIMQLTKLESKVL
NgeV (JKT9982)      LYLCVTDTKRVTFYEQCYINYTKAHPTYAISKEQFMSLIQLYLHQDYTLPVNQLANRIRVSLQLSSYGVVRPYHQLIMQLTKLESKVL
CavV                LYLCVTDTKRVTFYEQCYINYTKAHPTYAISKEQFMGLIQLYLHQDYTLPVNQLANRIRVSLQLSSYGVVRPYHQLIMQLTKLESKVL
HanaV               LYLCVTDNRRITFYEQCYINYTKSHPDYAISKQQFMGLIQQYLHQDYTLPTNQLANRIRVSLQLSSYGVVRPYHQLIMQLTKLESKVL
KPhV (KP84_0156)    LYLCVTNPRPVTFYEQCYINYTKDHPMYVISKQQFMGLIQTYLHQDYTLPINQLANRIRVSLQLSSYGVVRPYHQLIMQLTKLESKVL
KPhV (KP84_0192)    LYLCVTNPRPVTFYEQCYINYTKDHPMYVISKQQFMGLIQTYLHQDYTLPINQLANRIRVSLQLSSYGVVRPYHQLIMQLTKLESKVL
KphV (KP84_0344)    LYLCVTNPRPVTFYEQCYINYTKDHPMYVISKQQFMGLIQTYLHQDYTLPINQLANRIRVSLQLSSYGVVRPYHQLIMQLTKLESKVL
BBaV (JKT9876)      LYLCVTNTRQVTFYEQCYINYTKDHPLYVIDKQKFMGLIQMYLHQDYTLPINQLANRIRVSLQLSSYGVVRPYHQLIMQLTKLESKVL
BBaV (JKT9891)      LYLCVTNTRQVTFYEQCYINYTKDHPLYVIDKQKFMGLIQMYLHQDYTLPINQLANRIRVSLQLSSYGVVRPYHQLIMQLTKLESKVL
BBaV (JKT9853)      LYLCVTNTRQVTFYEQCYINYTKDHPLYVIDKQKFMGLIQMYLHQDYTLPINQLANRIRVSLQLSSYGVVRPYHQLIMQLTKLESKVL
BBaV (JKT7774)      LYLCVTNTRQVTFYEQCYINYTKDHPLYVIDKQKFMGLIQMYLHQDYTLPINQLANRIRVSLQLSSYGVVRPYHQLIMQLTKLESKVL
KSaV (JKT10701)     LYLCVTNTRQITFYEQCYINYTKDHPLYVISKQKFMGLIQTYLHQDYTLPINQLANRIRVSLQLSSYGVVRPYHQLIMQLTKLESKVL
NseV                LYYCVTAPRRISFFEQCYTNYTQAHPHKLIIKEDFMSLVQTYLHQDYTLPANQLANRIRVRMQLSSYGIVRPYYQLVMQLTKLESRVL
MenoV               LYLCVTEAKSVSFYEQCYRSYIATHPTYVVTHEKFMSLVQLYLYQDYSIPSNQLANRIRILLQLSSYGIVRPYYQLIMQLTKIESKVI
                    ** ***  : ::*:****  .*  **   : ::.**.*:* **:***::* ********: :******:****:**:*****:**:*:

HouV (16757)        DSSVVDIPITLINSQEIGTYYIEIPREHKLDQHSTYSYLIGTREVSFTPNYYRLSSTNTHIWQTDTQIPNYCTFIRQRRLNTLSAILR
HouV (V3872)        DSSVVDIPITLINSQEIGTYYIEIPREHKLDQHSTYSYLIGTREVSFTPNYYRLSSTNTHIWQTDTQIPNYCTFIRQRRLNTLSAILR
HouV (16740)        DSSVVDIPITLINSQEIGTYYIEIPREHKLDQHSTYSYLIGTREVSFTPNYYRLSSTNTHIWQTDTQIPNYCTFIRQRRLNTLSAILR
HouV (V3982)        DSSVVDIPITLINSQEIGTYYIEIPREHKLDQHSTYSYLIGTREVSFTPNYYRLSSTNTHIWQTDTQIPNYCTFIRQRRLNTLSAILR
NDiV                DSSVVDIPITLINSQEIGTYYIEIPREHKLDQHSTYSYLLGTREVSFTPNYYRLSSTNTHIWQTDTQIPNYCTFIRQRRLNTLSAILR
NgeV (JKT9982)      DSSVVDIPITLINSQEIGTYYIEIPREHKLDQHSTYSYLIGTREVSFTPNYYRLSSTNTHIWQTDTQIPNYCTFIRQRRLNTLSAILR
CavV                DSSVVDIPITLINSQEIGTYYIEIPREHKLDQHSTYSYLMGTREVSFTPNYHRLSSTNTHIWLTDTQIPNYCTFIRQRRLNTLSAILR
HanaV               DSSVVDIPVTLINSQEIGTYYIEIPREHKLDLHSTYSYLLGTREVSFTPNYYRLSSTNTHIWQTDTQIPNSCTFIRQRRLNTLSAILR
KPhV (KP84_0156)    DSSVVDIPITLINSQEIGTYYIEIPREHKLDQHSTYSYLIGTREVSFTPTYYRLSSTNTHIWQTDTQIPNYCTFIRQRRLNTLSAILR
KPhV (KP84_0192)    DSSVVDIPITLINSQEIGTYYIEIPREHKLDQHSTYSYLIGTREVSFTPTYYRLSSTNTHIWQTDTQIPNYCTFIRQRRLNTLSAILR
KphV (KP84_0344)    DSSVVDIPITLINSQEIGTYYIEIPREHKLDQHSTYSYLIGTREVSFTPTYYRLSSTNTHIWQTDTQIPNYCTFIRQRRLNTLSAILR
BBaV (JKT9876)      DSSVVDIPITLINSQEIGTYHIEIPREHKLDQHSTYSYLIGNREVSFTPSYYRLSSTNTHIWQTDTQIPNYCTFIRQRRLNTLSAILR
BBaV (JKT9891)      DSSVVDIPITLINSQEIGTYHIEIPREHKLDQHSTYSYLIGNREVSFTPSYYRLSSTNTHIWQTDTQIPNYCTFIRQRRLNTLSAILR
BBaV (JKT9853)      DSSVVDIPITLINSQEIGTYHIEIPREHKLDQHSTYSYLIGNREVSFTPSYYRLSSTNTHIWQTDTQIPNYCTFIRQRRLNTLSAILR
BBaV (JKT7774)      DSSVVDIPITLINSQEIGTYHIEIPREHKLDQHSTYSYLIGNREVSFTPSYYRLSSTNTHIWQTDTQIPNYCTFIRQRRLNTLSAILR
KSaV (JKT10701)     DSSVVDIPITLINSQEIGTYYIEIPREHKLDQHSTYSYLIGTREVSFTPSYYRLSSTNTHIWQTDTQIPNYCTFIRQRRLNTLSAILR
NseV                DASVVDIPITPTNIQEIGTYHIEIPQEYKLDQHSTYAYLIGSREVVFTPTYYRLSSKNTHIWQTDIQIPNYSTFIRQRRLNTLSAIIR
MenoV               DASVVDIPITPTNIQEAGSFYIEVPLEYKLDQHSSYSYLIGTREVTINLTHHNLSSNKTYIWKTDVQIPNYCTFIRQRRLNTLSAILR
                    *:******:*  * ** *:::**:* *:*** **:*:**:*.*** :. .::.***.:*:** ** **** .**************:*

HouV (16757)        NTTQHVPEFTRLLLEWNQQLPITAKPFAEFKPSLKIPAQPNVTDNINTLLKELNEKRFKIMFGGPGTGKSHTLSILINHLHEKGLRIL
HouV (V3872)        NTTQHVPEFTRLLLEWNQQLPITAKPFAEFKPSLKIPAQPNVTDNINTLLKELNEKRFKIMFGGPGTGKSHTLSILINHLHEKGLRIL
HouV (16740)        NTTQHVPEFTRLLLEWNQQLPITAKPFAEFKPSLKIPAQPNVTDNINTLLKELNEKRFKIMFGGPGTGKSHTLSILINHLHEKGLRIL
HouV (V3982)        NTTQHVPEFTRLLLEWNQQLPITAKPFAEFKPSLKIPAQPNVTDNINTLLKELNEKRFKIMFGGPGTGKSHTLSILINHLHEKGLRIL
NDiV                NTTQHVPEFTRLLLEWNQQLPITAKPFAEFKPSLKIPAQPNVTDNINTLLKELNVKRFKIMFGGPGTGKSHTLSILINHLHEKGLRIL
NgeV (JKT9982)      NTTQHVPEFTRLLLEWNQQLPITAKPFAEFKPSLKIPAQPNVTDNINTLLKELNAKRFKIMFGGPGTGKSHTLSILINHLHEKGLRIL
CavV                NTTQHVPEYTRLLLEWNQQLPITAKPFAEFKPSLKIPAQPNVTDNINMLLKELNEKRFKIMFGGPGTGKSHTLSILINHLHEKGLRIL
HanaV               NTTQHVPEFTRALLEWNQQLPSETKPLPEFKPSLKIPAQPNVTDNINILLKELNTKRFKIMFGGPGTGKSHTLATLINHLHDKGFRVL
KPhV (KP84_0156)    NTTQHVPEFTRLLLEWNQQLPITAKPFAVFKPSLKIPAQPNVTDNINILLSELNSKRFKIMFGGPGTGKSHTLSILINHLHEKGLRIL
KPhV (KP84_0192)    NTTQHVPEFTRLLLEWNQQLPITAKPFAVFKPSLKIPAQPNVTDNINILLSELNSKRFKIMFGGPGTGKSHTLSILINHLHEKGLRIL
KphV (KP84_0344)    NTTQHVPEFTRLLLEWNQQLPITAKPFAVFKPSLKIPAQPNVTDNINILLSELNSKRFKIMFGGPGTGKSHTLSILINHLHEKGLRIL
BBaV (JKT9876)      NTTQHVPEYTRLLLEWNQQLPITAKPFPDFRPSTKIPAQPNVTDNINILLGELNSKRFKIMFGGPGTGKSHTLSILINHLHEKGLRIL
```

```
BBaV (JKT9891)      NTTQHVPEYTRLLLEWNQQLPITAKPFPDFRPSTKIPAQPNVTDNINILLGELNSKRFKIMFGGPGTGKSHTLSILINHLHEKGLRIL
BBaV (JKT9853)      NTTQHVPEYTRLLLEWNQQLPITAKPFPDFRPSTKIPAQPNVTDNINILLGELNSKRFKIMFGGPGTGKSHTLSILINHLHEKGLRIL
BBaV (JKT7774)      NTTQHVPEYTRLLLEWNQQLPITAKPFPDFRPSTKIPAQPNVTDNINILLGELNSKRFKIMFGGPGTGKSHTLSILINHLHEKGLRIL
KSaV (JKT10701)     NTTQHVPEYTRLLLEWNQQLPITAKPFAEFKPSLKISAQPNVTANINILLKELNSKRFKIMFGGPGTGKSHTLSILINHLHEKGFRIL
NseV                NTTQHVPEFTRLLLAWNQQLPILAKRFPIFKPSLKISAEPNVVDNINVLLSELNEKRFKIMFGGPGTGKSYTLSILINHLHESGFKIL
MenoV               NTTQRVPEFTRLLLSWNQQLPISARTFPRFTPSLKIPSQPNVAQNINTLLNDLNEHRFKIMFGGPGTGKSHTLSILVNHLHEQGFKIL
                    ****:***:** ** ******   :: :.  * ** **.::***. *** ** :** :**************:**: *:****:.*::*
HouV (16757)        VYTPSHQSANALLYKIANLIKRRTIQNPGLVRIITDGMKEEIKPHPYITYRTNMLDKDRICVTTIQSFSTVQHVKDVDLVILDEFSLT
HouV (V3872)        VYTPSHQSANALLYKIANLIKRRTIQNPGLVRIITDGMKEEIKPHPYITYRTNMLDKDRICVTTIQSFSTVQHVKDVDLVILDEFSLT
HouV (16740)        VYTPSHQSANALLYKIANLIKRRTIQNPGLVRIITDGMKEEIKPHPYITYRTNMLDKDRICVTTIQSFSTVQHVKDVDLVILDEFSLT
HouV (V3982)        VYTPSHQSANALLYKIANLIKRRTIQNPGLVRIITDGMKEEIKPHPYITYRTNMLDKDRICVTTIQSFSTVQHVKDVDLVILDEFSLT
NDiV                VYTPSHQSANALLYKIANLIKRRTIQNPGLVRIITDGMKEEIKPHPYITYRTNMLDKDRICVTTIQSFSTVQHVKDVDLVILDEFSLT
NgeV (JKT9982)      VYTPSHQSANALLYKIANLIKRRTIQNPGLVRIITDGMKEEIKPHPYITYRTNMLDKDRICVTTIQSFSTVQHVKDVDLVILDEFSLT
CavV                VYTPSHQSANALLYKIANLIKRRTIQNPGLVRIITDGMKEEIKPHPYITYRTNMLDKDRICVTTIQSFSTVQHVKDIDLVILDEFSLT
HanaV               VYTPSHQSANALLYKIANMMKRRGIQNPGLVRIITDGMKDELKPHPYITYRTHMIDTDRICVTTIQSFSAVQHVV-VDVVILDEFSLT
KPhV (KP84_0156)    VYTPSHQSANALLYKIANLIKRRNIQNPGLVRIITDGMKDEIKPHPYITYRTNMLDKDRICVTTIQSFSTVQHVKDIDLVILDEYSLT
KPhV (KP84_0192)    VYTPSHQSANALLYKIANLIKRRNIQNPGLVRIITDGMKDEIKPHPYITYRTNMLDKDRICVTTIQSFSTVQHVKDIDLVILDEYSLT
KphV (KP84_0344)    VYTPSHQSANALLYKIANLIKRRNIQNPGLVRIITDGMKDEIKPHPYITYRTNMLDKDRICVTTIQSFSTVQHVKDIDLVILDEYSLT
BBaV (JKT9876)      VYTPSHQSANALLYKIANMMKRRNIQNPGLVRIITDGLKDEIKPHPYITYRTNMLDKDRICVTTIQSFSTVQHVKDIDLVILDEYSLT
BBaV (JKT9891)      VYTPSHQSANALLYKIANMMKRRNIQNPGLVRIITDGLKDEIKPHPYITYRTNMLDKDRICVTTIQSFSTVQHVKDIDLVILDEYSLT
BBaV (JKT9853)      VYTPSHQSANALLYKIANMMKRRNIQNPGLVRIITDGLKDEIKPHPYITYRTNMLDKDRICVTTIQSFSTVQHVKDIDLVILDEYSLT
BBaV (JKT7774)      VYTPSHQSANALLYKIANMMKRRNIQNPGLVRIITDGLKDEIKPHPYITYRTNMLDKDRICVTTIQSFSTVQHVKDIDLVILDEYSLT
KSaV (JKT10701)     VYTPSHQSANALLYKIANLIKRRNIQNPGLVRIITDGMKDEIKPHPYITYRTNMLDKDRICVTTIQSFSTVQHVKDIDLVILDEYSLT
NseV                IYTPSHQSANALLYKIANVMRKRNIQNPRLVRIITEGMKDELKPHPYITPRTHMLDQDCICVTTIQSFPTVQHVD-VDLVILDEFSLT
MenoV               IYTPSHQSANALLFKIANIMKRRHIQNSGLVRIITEGMKDEIKPHPFITYRASMLEKDRICVTTIQSFSTVQHVRDIDLVILDEFSLT
                    :***********:****::::* ***. ******:*:*:*:****:** *: *:: * *********.:****  :*:*****:***

HouV (16757)        SDNYLLTGLAHLKPSTRVLFSGDPRQLSGVDEIRKPLQSRFHTLINYYTETYPREVHVLKYHFRCHPSIFQYFKDLYYADKDMECATS
HouV (V3872)        SDNYLLTGLAHLKPSTRVLFSGDPRQLSGVDEIRKPLQSRFHTLINYYTETYPREVHVLKYHFRCHPSIFQYFKDLYYADKDMECATS
HouV (16740)        SDNYLLTGLAHLKPSTRVLFSGDPRQLSGVDEIRKPLQSRFHTLINYYTETYPREVHVLKYHFRCHPSIFQYFKDLYYADKDMECATS
HouV (V3982)        SDNYLLTGLAHLKPSTRVLFSGDPRQLSGVDEIRKPLQSRFHTLINYYTETYPREVHVLKYHFRCHPSIFQYFKDLYYADKDMECATS
NDiV                SDNYLLTGLAHLKPSTRVLFSGDPRQLSGVDEIRKPLQSRFHTLINYYTETYPREVHVLKYHFRCHPSIFQYFKDLYYADKDMECATS
NgeV (JKT9982)      SDNYLLTGLAHLKPSTRVLFSGDPRQLSGVDEIRKPLQSRFHTLINYYTETYPREVHVLKYHFRCHPSIFQYFKDLYYADKDMECATS
CavV                SDNYLLTGLAHLKPSTRVLFSGDPRQLSGVDEVRKPLQSRFHTLINYYTETYPREVHVLKYHFRCHPTIFQYFKDLYYADKDMECATS
HanaV               SDNYLLSGLAHLRLSTRVLFSGDPRQLSGVDVIRKLLHSRFHTLINYYTETYPLEVLVLTYHFRCHPMIFQYFKDLYYADKHMECATS
KPhV (KP84_0156)    SDNYLLTGLSHLKPSTRVLFSGDPRQLSGVDEIRKPLPARFHTLINYYTETYPLEVHVLKYHFRCHPTIFQYFKDLYYADKDMECATS
KPhV (KP84_0192)    SDNYLLTGLSHLKPSTRVLFSGDPRQLSGVDEIRKPLPARFHTLINYYTETYPLEVHVLKYHFRCHPTIFQYFKDLYYADKDMECATS
KphV (KP84_0344)    SDNYLLTGLSHLKPSTRVLFSGDPRQLSGVDEIRKPLPARFHTLINYYTETYPLEVHVLKYHFRCHPTIFQYFKDLYYADKDMECATS
BBaV (JKT9876)      SDNYLLTGLAHLKPSTRVLFSGDPRQLSGVDEIRKPLPARFHTLINYYTETYPLEVHVLKYHFRCHPSIFHYFKDLFYADKDMECATS
BBaV (JKT9891)      SDNYLLTGLAHLKPSTRVLFSGDPRQLSGVDEIRKPLPARFHTLINYYTETYPLEVHVLKYHFRCHPSIFHYFKDLFYADKDMECATS
BBaV (JKT9853)      SDNYLLTGLAHLKPSTRVLFSGDPRQLSGVDEIRKPLPARFHTLINYYTETYPLEVHVLKYHFRCHPSIFHYFKDLFYADKDMECATS
BBaV (JKT7774)      SDNYLLTGLAHLKPSTRVLFSGDPRQLSGVDEIRKPLPARFHTLINYYTETYPLEVHVLKYHFRCHPSIFHYFKDLFYADKDMECATS
KSaV (JKT10701)     SDNYLLTGLAHLKPSTRVLFSGDPRQLSGVDEIRKPLPSRFHTLINYYTETYPLEVHVLKYHFRCHPSIFQYFKELYYADKDMECATS
NseV                SDNYLLTGLSHLKLSTRVLFSGDPKQLSGVDEIRKPLLPRFHTLINYYAETYPKELLVLKHHFRCHPSIFQFFKDLYYADKDMVCATA
MenoV               SDNYLLTGLSHLKPSTRVLFSGDPRQLSGVDETRKPLQPRFHTLINFYTETYPNEVHVLKDHFRCHPHIFQYFKDQYYADKDMVCATK
                    ******:**:**: **********:******  ** * .*******:*:**** *: **. ****** **::**: :****.* ***

HouV (16757)        IADRIIRPLNPINTVQVSEPTFRNQGVILNQDEADKVLEILVLVNQTLALHSSYEYQPTIAIICSYKSQLQNFISLQQQKILSENVNL
HouV (V3872)        IADRIIRPLNPINTVQVSEPTFRNQGVILNQDEADKVLEILVLVNQTLALHSSYEYQPTIAIICSYKSQLQNFISLQQQKILSENVNL
HouV (16740)        IADRIIRPLNPINTVQVSEPTFRNQGVILNQDEADKVLEILVLVNQTLALHSSYEYQPTIAIICSYKSQLQNFISLQQQKILSENVNL
HouV (V3982)        IADRIIRPLNPINTVQVSEPTFRNQGVILNQDEADKVLEILVLVNQTLALHSSYEYQPTIAIICSYKSQLQNFISLQQQKILSENVNL
NDiV                IADRIIRPLNPINTVQVSEPTFRNQGVILNQDEADKVLEILVLVNQTLALHSSYEYQPTIAIICSYKSQLQNFISLQQQKILSENVNL
NgeV (JKT9982)      IADRIIRPLNPINTVQVSEPTFRNQGVILNQDEADKVLEILVLVNQTLALHSSYEYQPTIAIICSYKSQLQNFISLQQQKILSENVNL
CavV                IADRIIRPLNPINTVQVSEPTFRNQGVILNQDEADKVLEILVLVNQTLALHSSYEYQPTIAIICSYKSQLQNFISLQQQKILSDNVNL
HanaV               IADRIIRPLNPINTVQVGEPTFRNQGVILNQDEADKVLEILVLVNQTLALHSTYEYQPTIAIICSYKSQLQNFISLQQQKILSDNVTL
KPhV (KP84_0156)    VADRIIRPLNPINTVQVSEPTYRNQGVILNQDETDKVLEILVLVNQTLALHSSYEYQPTIAIICSYKSQLQNFISLQQQKIIPEYVNL
KPhV (KP84_0192)    VADRIIRPLNPINTVQVSEPTYRNQGVILNQDETDKVLEILVLVNQTLALHSSYEYQPTIAIICSYKSQLQNFISLQQQKIIPEYVNL
KphV (KP84_0344)    VADRIIRPLNPINTVQVSEPTYRNQGVILNQDETDKVLEILVLVNQTLALHSSYEYQPTIAIICSYKSQLQNFISLQQQKIIPEYVNL
BBaV (JKT9876)      IADRIIRPLNPINTVQVSEPTFRNQGVILNQDEADKVLEILVLVNQTLALHSSYEYQPTIAIICSYKSQLQNFISLQQQKVISEYVKL
BBaV (JKT9891)      IADRIIRPLNPINTVQVSEPTFRNQGVILNQDEADKVLEILVLVNQTLALHSSYEYQPTIAIICSYKSQLQNFISLQQQKVISEYVKL
BBaV (JKT9853)      IADRIIRPLNPINTVQVSEPTFRNQGVILNQDEADKVLEILVLVNQTLALHSSYEYQPTIAIICSYKSQLQNFISLQQQKVISEYVKL
BBaV (JKT7774)      IADRIIRPLNPINTVQVSEPTFRNQGVILNQDEADKVLEILVLVNQTLALHSSYEYQPTIAIICSYKSQLQNFISLQQQKVISEYVKL
KSaV (JKT10701)     IADRIIRPLNPINTVQVSEPTFRNQGVILNQDEADKVLEILVLVNQTLALHSSYEYQPTIAIICSYKSQLQNFISLQQQKIISEYVNL
NseV                ASDRIIRPLHPINTIQVEGPSYRNQGVIRNENETEKVLDILVLVSQVLSLHASFNYQPTIAIICSYKSQLQNFASLQQLKVIPEYVNL
MenoV               AEDRLIRPLNPINTVHVGPPTYRNQGVILNQDETDKVLDILILVTQTLTLHASQEYQPSLAIICSYKSQLQHFATLQNQKIIPEFVKL
                      **:****:****::*  *::****** *::*::***:**:**.*.*:**:: :***::***********:* :**: *::.: *.*

HouV (16757)        STIDSAQGDEFDIVILCLSQINNFTLNPNRFNVAISRAKSVLFITVPPIDKNPAFLFKDVYATLHKHNLTYFKIYNTSGKAILSLDSP
HouV (V3872)        STIDSAQGDEFDIVILCLSQINNFTLNPNRFNVAISRAKSVLFITVPPIDKNPAFLFKDVYATLHKHNLTYFKIYNTSGKAILSLDSP
HouV (16740)        STIDSAQGDEFDIVILCLSQINNFTLNPNRFNVAISRAKSVLFITVPPIDKNPAFLFKDVYATLHKHNLTYFKIYNTSGKAILSLDSP
HouV (V3982)        STIDSAQGDEFDIVILCLSQINNFTLNPNRFNVAISRAKSVLFITVPPIDKNPAFLFKDVYATLHKHNLTYFKIYNTSGKAILSLDSP
NDiV                STIDSAQGDEFDIVILCLSQINNFTLNPNRFNVAISRAKSVLFITVPPIDKNPAFLFKDVYATLHKHNLTYFKIYNTSGKAILSLDSP
NgeV (JKT9982)      STIDSAQGDEFDIVILCLSQINNFTLNPNRFNVAISRAKSVLFITVPPIDKNPAFLFKDVYATLHKHNLTYFKIYNTSGKAILSLDSP
CavV                STIDSAQGDEFDIVILCLSQINNFTLNPNRFNVAISRAKSVLFITVPPIDKNPAFLFKDVYETLHKHNLTYFKIYNTSGKAILSLDSP
HanaV               STIDSAQGDEFDIVILCLSQINNFTLNPNRFNVAISRAKSVLFITVPPIDKNPSFLYKDVYTTLNKHNLTYFKIYNTSGKAILSLDSP
KPhV (KP84_0156)    STTDSAQGDEFDIVILCLSQINNFTLNPNRFNVAISRAKSVLFITVPPIDKNPSFLFKDVYMTLNKHNLTYFKIYNNSGKAILSLDTP
KPhV (KP84_0192)    STTDSAQGDEFDIVILCLSQINNFTLNPNRFNVAISRAKSVLFITVPPIDKNPSFLFKDVYMTLNKHNLTYFKIYNNSGKAILSLDTP
KphV (KP84_0344)    STTDSAQGDEFDIVILCLSQINNFTLNPNRFNVAISRAKSVLFITVPPIDKNPSFLFKDVYMTLNKHNLTYFKIYNNSGKAILSLDTP
BBaV (JKT9876)      STIDSAQGDEFDIVILCLSQINNFTLNPNRFNVAISRAKSVLFITVPPIDTNPSFLFKDVYATLCKHNLTYFKIYNTSGKAILSLDTP
```

```
BBaV (JKT9891)     STIDSAQGDEFDIVILCLSQINNFTLNPNRFNVAISRAKSVLFITVPPIDTNPSFLFKDVYATLCKHNLTYFKIYNTSGKAILSLDTP
BBaV (JKT9853)     STIDSAQGDEFDIVILCLSQINNFTLNPNRFNVAISRAKSVLFITVPPIDTNPSFLFKDVYATLCKHNLTYFKIYNTSGKAILSLDTP
BBaV (JKT7774)     STIDSAQGDEFDIVILCLSQINNFTLNPNRFNVAISRAKSVLFITVPPIDTNPSFLFKDVYATLCKHNLTYFKIYNTSGKAILSLDTP
KSaV (JKT10701)    STIDSAQGDEFDIVILCLSQINNFTLNPNRFNVAISRAKSVLFITVPPIDNNPSFLFKDVYATLCKHNLTYFKIYNNSGKAILSLDTP
NseV               STIDSAQGDEFDIVILCLSQINNFTLNPNRFNVAISRAKSVLFITVPPVDNNPAFLYRDVYAKLQAYELTYFKIFNNSGKAIIELNSP
MenoV              STIDSAQGDEFDIVILCLSQINNFTLNPNRFNVAISRAKSVLFITVPPIDQNPAFLYQDVYKTLGQHNLSYFRIFNNSGK-VVKVTLS
                   ** *******************************************:* **:**::*** .*  ::*:**:*:*.*** ::.:  .
HouV (16757)       TTLKTKAEKMTYTNVRHLDKNTHTMQRKFPMNIVMDDCICFDAEFLNPRDNVQEPVMLSYGFSSKYGKRRIAGIPVRYIKDKFNRIVP
HouV (V3872)       TTLKTKAEKMTYTNVRHLDKNTHTMQRKFPMNIVMDDCICFDAEFLNPRDNVQEPVMLSYGFSSKYGKRRIAGIPVRYIKDKFNRIVP
HouV (16740)       TTLKTKAEKMTYTNVRHLDKNTHTMQRKFPMNIVMDDCICFDAEFLNPRDNVQEPVMLSYGFSSKYGKRRIAGIPVRYIKDKFNRIVP
HouV (V3982)       TTLKTKAEKMTYTNVRHLDKNTHTMQRKFPMNIVMDDCICFDAEFLNPRDNVQEPVMLSYGFSSKYGKRRIAGIPVRYIKDKFNRIVP
NDiV               TTLKTKAEKMTYTNVRHLDKNTHTMQRKFPMNIVMDDCICFDAEFLNPRDNVQEPVMLSYGFSSKYGKRRIAGIPVRYIKDKFNRIVP
NgeV (JKT9982)     TTLKTKAEKMTYTNVRHLDKNTHTMQRKFPMNIVMDDCICFDAEFLNPRDN-QEPVMLSYGFSSKYGKRRIAGIPVRYIKDKFNRIVP
CavV               TTLKTQAEKMPYTNVRNLDKETHTMQRKFPMNIVMDDCICFDAEFLNPRDN-QEPVMLSYGFSSKYGKRRIAGIPVRYIKDKFNRIIP
HanaV              TTLKNQAE-MSFINIRQLN--SHTMERKFPINIVMPDYICFDAEFLNPRDNVQEHVMLSYGFSSKYGKRRIAGTPVRYIKDKFNKIVP
KPhV (KP84_0156)   TTLKTKANKA-YINIRHLDKATHTMQRKFPINIVMSDYICFDAEFLNPKDNLQEPVMLSYGFSSKYGKRRIAGIPVRYIKDRFDKIVP
KPhV (KP84_0192)   TTLKTKANKA-YINIRHLDKATHTMQRKFPINIVMSDYICFDAEFLNPKDNLQEPVMLSYGFSSKYGKRRIAGIPVRYIKDRFDKIVP
KphV (KP84_0344)   TTLKTKANKA-YINIRHLDKATHTMQRKFPINIVMSDYICFDAEFLNPKDNLQEPVMLSYGFSSKYGKRRIAGIPVRYIKDRFDKIVP
BBaV (JKT9876)     TTLKTKAGKMSYINIRNL---THTMQRKFPINIVMNDYICFDAEFLNPRDNLQEPVMLSYGFSSKYGKRRIAGIPVRYIKDRFDKIVP
BBaV (JKT9891)     TTLKTKAGKMSYINIRNL---THTMQRKFPINIVMNDYICFDAEFLNPRDNLQEPVMLSYGFSSKYGKRRIAGIPVRYIKDRFDKIVP
BBaV (JKT9853)     TTLKTKAGKMSYINIRNL---THTMQRKFPINIVMNDYICFDAEFLNPRDNLQEPVMLSYGFSSKYGKRRIAGIPVRYIKDRFDKIVP
BBaV (JKT7774)     TTLKTKAGKMSYINIRNL---THTMQRKFPINIVMNDYICFDAEFLNPRDNLQEPVMLSYGFSSKYGKRRIAGIPVRYIKDRFDKIVP
KSaV (JKT10701)    TTLKTKAGKMSYINIRNLDKTTHTMQRKFPINIVMNDYICFDAEFLNPRDNLQEPVMLSYGFSSKYGKRRIAGIPVRYIKDRFNQIVP
NseV               TTLQNKASES-FMNIRQLPKEDYTMKRRFPMNVVLDDYIYFDSEFLNPKDNPPEPVMLSYGFSSKHGKRRISGTPMRYIKDNYNRITP
MenoV              TTLHNQSAPLEYINIRNIASSKQTLQRKYPINVIMDDYIFFDSEFLNPRDDPREPVMLSYGFTSAYGNRRISGTPMRYVYSKFNQLTP
                   ***:.::    : *:*::     *::*::*:*::: * * **:*****:*:  * *******:* :*:***:* *:**: ..:::: *

HouV (16757)       QKYNYKDNNKPLTSTYTCDWMRKQHPEQYKHLLTSVMQGIRNDTTVDLKPLLNFCVDNMHVKPVIITWSGASDHCFLKAHTLYPDIST
HouV (V3872)       QKYNYKDNNKPLTSTYTCDWMRKQHPEQYKHLLTSVMQGIRNDTTVDLKPLLNFCVDNMHVKPVIVTWSGASDHCFLKAHTLYPDIST
HouV (16740)       QKYNYKDNNKPLTSTYTCDWMRKQHPEQYKHLLTSVMQGIRNDTTVDLKPLLNFCVDNMHVKPVIVTWSGASDHCFLKAHTLYPDIST
HouV (V3982)       QKYNYKDNNKPLTSTYTCDWMRKQHPEQYKHLLTSVMQGIRNDTTVDLKPLLNFCVDNMHVKPVIVTWSGASDHCFLKAHTLYPDIST
NDiV               QKYNYKDNNKPLTSTYTCDWMRKQHPEQYKHLLTSVMQGIRNDTTVDLKPLLNFCVDNMHVKPVIVTWSGASDHCFLKAHTLYPDIAT
NgeV (JKT9982)     QKYNYKDNNKPLTSTYTCDWMRKQHPEQYKHLLTSVMQGIRNDTTVDLKPLLNFCVDNMHVKPVIVTWSGASDHCFLKAHTLYPDIST
CavV               HKYNYKDNNKPLTSTYSCDWMKKQHPDQYKHLLTSVLQGIRNDTTVDLRPLLNFCVDNMHVKPVIVTWSGASDHCFLRAHTLYPDIST
HanaV              IKYPYKDKHKPLTSTYVCEWMKKQKPEQYQYLLNSITQGISNDTIVDLKPLLNFCVDNVHVKPVIVTWSGVSDHCFLKAYTLYPDIST
KPhV (KP84_0156)   QEYNYRDTNKPLTSTYNCEWMRKQHPDQYKHLLHSVTQGIRNSTIVDLKPLLNFCVDNMHVKPVIVTWAGASDHCFLKANTLYPDIST
KPhV (KP84_0192)   QEYNYRDTNKPLTSTYNCEWMRKQHPDQYKHLLHSVTQGIRNSTIVDLKPLLNFCVDNMHVKPVIVTWAGASDHCFLKANTLYPDIST
KphV (KP84_0344)   QEYNYRDTNKPLTSTYNCEWMRKQHPDQYKHLLHSVTQGIRNSTIVDLKPLLNFCVDNMHVKPVIVTWAGASDHCFLKANTLYPDIST
BBaV (JKT9876)     HEYSYKDNNKPLTSTYNCDWMRKRDPEQYKHLLNSVMQGINNSTIVDLRPLLNFCVDNIHVKPVIITWAGASDHCFLKANTLYPDIST
BBaV (JKT9891)     HEYSYKDNNKPLTSTYNCDWMRKRDPEQYKHLLNSVMQGINNSTIVDLRPLLNFCVDNIHVKPVIITWAGASDHCFLKANTLYPDIST
BBaV (JKT9853)     HEYSYKDNNKPLTSTYNCDWMRKRDPEQYKHLLNSVMQGINNSTIVDLRPLLNFCVDNIHVKPVIITWAGASDHCFLKANTLYPDIST
BBaV (JKT7774)     HEYSYKDNNKPLTSTYNCDWMRKRDPEQYKHLLNSVMQGINNSTIVDLRPLLNFCVDNIHVKPVIITWAGASDHCFLKANTLYPDIST
KSaV (JKT10701)    HEYSYKDNNKPLTSTYNCDWMRKRYPEQYKHLLNSVTQGIHNLTTVDLRPLLNFCVDNMHVKPVIVTWAGASDHCFLKANTLYPDIST
NseV               IKYNFKDKDKPLTSTYVSSWMKKDLPTQFKHLNNAVCKGISDNTIVDVKHMLNFCIDNVHVKPVIVTWAGTSDHCFLRANTLYPDVSQ
MenoV              VKYNYKDNNKPLTSTYIATWLKAKAPEQYNYLRDTITQGIKNNVTVDIKHLLNFCIDNMHVKPVIITWAGDNDHCFLKANTLYPPAAQ
                    :* ::*..*******  . *::   * *:::*  :: :** : . **:: :****:**:******:**:* .*****:* ****  :

HouV (16757)       VCNITTRCTSQPIYASPQGRHTYYLCQYHAHQLKDHINITHFVNLEIIDLK VDRNQYTDERTLRVYHNDYLKLTLDLDN-VASNSLT
HouV (V3872)       VCNITTRCTSQPIYASPQGRHTYYLCQYHAHQLKDHINITHFVNLEIIDLK VDRNQYTDERTLRVYHNDYLKLTLDLDN-VASNSLT
HouV (16740)       VCNITTRCTSQPIYASPQGRHTYYLCQYHAHQLKDHINITHFVNLEIIDLK VDRNQYTDERTLRVYHNDYLKLTLDLDN-VASNSLT
HouV (V3982)       VCNITTRCTSQPIYASPQGRHTYYLCQYHAHQLKDHINITHFVNLEIIDLK VDRNQYTDERTLRVYHNDYLKLTLDLDN-VASNSLT
NDiV               VCNITIRCTSQPIYASPQGRHTYYLCQYHAHQLKDHVNITHFVNLEIIDLK VDRNQYTDERTLRVYHNDYLKLTLDLDN-VASNSLT
NgeV (JKT9982)     VCNITTRCTSQPIYASPQGRHTYYLCQYHAHQLKDHINITHFVNLEIIDLK VDRNQYTDERTLRVYHNDYLKLTLDLDN-VASNSLT
CavV               VCNITTRCTSQPIYASPQGRHTYYLCQYHAHQLKDHINITHFVNLEIIDLK VDRNQYTNERTLRVYHNDYLKLTLDLDN-VASNSLT
HanaV              VCNITTRCTSQPIYASPQGRHTYYLCQYHAHQLKDHINITHFVNLEIIDLK VEYNQFTSERILRVYHNNYLKLTLDLDN-VASNSLT
KPhV (KP84_0156)   VCNITTRCSSQPVYASPQGLHTYYLCQYHAHQLQGYVNITHFVNLEIIDLK VNYNQYTGERILNVFHNENLKLELKLEN-VASNSLT
KPhV (KP84_0192)   VCNITTRCSSQPVYASPQGLHTYYLCQYHAHQLQGYVNITHFVNLEIIDLK VNYNQYTGERILNVFHNENLKLELKLEN-VASNSLT
KphV (KP84_0344)   VCNITTRCSSQPVYASPQGLHTYYLCQYHAHQLQGYVNITHFVNLEIIDLK VNYNQYTGERILNVFHNENLKLELKLEN-VASNSLT
BBaV (JKT9876)     VCNITTRCTSQPVYASPQGLHTYYLCQYHAHQHKESINITHFVNLEIIDLK INYNQYTGERILNVCHNDHLKLELDLDN-VASNSLA
BBaV (JKT9891)     VCNITTRCTSQPVYASPQGLHTYYLCQYHAHQHKESINITHFVNLEIIDLK INYNQYTGERILNVCHNDHLKLELDLDN-VASNSLA
BBaV (JKT9853)     VCNITTRCTSQPVYASPQGLHTYYLCQYHAHQHKESINITHFVNLEIIDLK INYNQYTGERILNVCHNDHLKLELDLDN-VASNSLA
BBaV (JKT7774)     VCNITTRCTSQPVYASPQGLHTYYLCQYHAHQHKESINITHFVNLEIIDLK INYNQYTGERILNVCHNDHLKLELDLDN-VASNSLA
KSaV (JKT10701)    VCNITTRCTSQPIYASPQGLHTYYLCQYHAHQHKESINITHFVNLEIIDLK VNYNQYTGERILNVCHNDNLKLELDLDN-VASNSLA
NseV               TCNIASRCLSQPIYASPQGRHTYYLCQYHAHKFKEQLNITHFVNLEIIDLS VDKNKHTDERNLRVTHNNELKLKLEISKSIASNKLQ
MenoV              ICNITPRCTSTPIYASAQTCHTYYLCQFHAQTFKEQLTITHFVNLEIIDLK AINNKQTNEKTLRLTHNNELKLTIELSQDIKSNSLN
                    ***: ** * *:***.*  *******:**:  :  :.************.    *: *.*: *.: **: *** :.:.: : **.*

HouV (16757)       DCHTRYCRTVHAPATPHDPLDDAIMTQCIYQSFVLSHLENLAYEPQANLKAFTSMDYRLKNFNPEMCKLRRELQKVWYTQYTTTN--K
HouV (V3872)       DCHTRYCRTVHAPATPHDPLDDAIMTQCIYQSFVLSHLENLAYEPQANLKAFTSMDYRLKNFNPEMCKLRRELQKVWYTQYTNTN--K
HouV (16740)       DCHTRYCRTVHAPATPHDPLDDAIMTQCIYQSFVLSHLENLAYEPQANLKAFTSMDYRLKNFNPEMCKLRRELQKVWYTQYTNTN--K
HouV (V3982)       DCHTRYCRTVHAPATPHDPLDDAIMTQCIYQSFVLSHLENLAYEPQANLKAFTSMDYRLKNFNPEMCKLRRELQKVWYTQYTNTN--K
NDiV               DCHTRYCRTVHAPATPHDPLDDAIMTQCIYQSFVLSHLENLAYEPQANLKAFTSMDYRLKNFNPEMCKLRRELQKVWYSQYTNTN--K
NgeV (JKT9982)     DCHTRYCRTVHAPATPHDPLDDAIMTQCIYQSFVLSHLENLAYEPQANLKAFTSMDYRLKNFNPEMCKLRRELQKVWYTQYTNTN--K
CavV               DCHTRYCRTIHAPPTPHDPLDDAIMTQCIYQSFVLSHLEKLAYEPQANLKAFTSMDYRLKNFNPEMCKLRRELQKVWYEKYIDTK--K
HanaV              DCHKRYCRTVHASITPHDPLDDAIMTQCIYQSFVLSHLENLAYDPQANLKAFTSMDYRLKNFNPEMCKLRRELQKTWYQQYIATN--K
KPhV (KP84_0156)   DCHARYCRTIHAPITPHDPLDDSIMTQCIYQSFVQLHLEAIAYEPQANLKAFTSMGYRLKNFNPEMCKLRRELQQAWYKQYVEHN--I
KPhV (KP84_0192)   DCHARYCRTIHAPITPHDPLDDSIMTQCIYQSFVQLHLEAIAYEPQANLKAFTSMGYRLKNFNPEMCKLRRELQQAWYKQYVEHN--I
KphV (KP84_0344)   DCHARYCRTIHAPITPHDPLDDSIMTQCIYQSFVQLHLEAIAYEPQANLKAFTSMGYRLKNFNPEMCKLRRELQQAWYKQYVEHN--I
BBaV (JKT9876)     DCHARYCRTIHAPITPHDPLDDSIMTQCIYQSFVQPHFEKLAYEPQANLKAFTSMDYRLKNFNPEMCKLRRELQQAWYAKYIEHN--I
```

```
BBaV (JKT9891)      DCHARYCRTIHAPITPHDPLDDSIMTQCIYQSFVQPHFEKLAYEPQANLKAFTSMDYRLKNFNPEMCKLRRELQQAWYAKYIEHN--I
BBaV (JKT9853)      DCHARYCRTIHAPITPHDPLDDSIMTQCIYQSFVQPHFEKLAYEPQANLKAFTSMDYRLKNFNPEMCKLRRELQQAWYAKYIEHN--I
BBaV (JKT7774)      DCHARYCRTIHAPITPHDPLDDSIMTQCIYQSFVQPHFEKLAYEPQANLKAFTSMDYRLKNFNPEMCKLRRELQQAWYAKYIEHN--I
KSaV (JKT10701)     DCHARYCRTIHAPITPHDPLDDAIMTQCIYQSFVQPHFEKIAYESEANLKAFTSMDYRLKNFDPEMCKLRRELQKAWYSEYITHN--I
NseV                DCHDFYCNTIHTPITPHDPLDDAIMTQCIFQSLVQPHFEKLAYEQGANLRAFLSMDYRLRNFNIEMCKLRRELQIEWYERYTSINTSR
MenoV               DCHAYYCNTTHRPITPHDPLDDALMTQCIFKSVVQPQLESIAYD-NANLNAFTSMDYRLRNFDPEMCKLRRDLQKHWYAKYSETS--I
                    ***  **.* * . ********::*****::*.*  ::* :**:  ***.** **.***:**: *******:**  ** .*   .
HouV (16757)        THCNMGCGKEPLQQALHNIDVLQGKSNPQNNMNTHTCDSEEHIYFDSHWYKDGGFIKPSYIFSDINKEHYYKLGTTGLCLYLNSKYA
HouV (V3872)        THCNMGCGKEPLQQALHNIDVLQGKSNPQNNMNTHTCDSEEHIYFDSHWYKDGGFTKPSYIFSDINKEHYYKLGTTGLCLYLNSKYA
HouV (16740)        THCNMGCGKEPLQQALHNIDVLQGKSNPQNNMNTHTCDSEEHIYFDSHWYKDGGFTKPSYIFSDINKEHYYKLGTTGLCLYLNSKYA
HouV (V3982)        THCNMGCGKEPLQQALHNIDVLQGKSNPQNNMNTHTCDSEEHIYFDSHWYKDGGFTKPSYIFSDINKEHYYKLGTTGLCLYLNSKYA
NDiV                THCNMGCGKEPLQQALHNIDVLQGKSNPQNNMNTHTCDSEEHIYFDSHWYKAGGFTKPSYIFSDINKEHYYKLGTTGLCLYLNSKYA
NgeV (JKT9982)      THCNMGCGKEPLQQALHNIDVLQGKSNPQNNMNTHTCDSEEHIYFDSHWYKDGGFTKPSYIFSDINKEHYYKLGTTGLCLYLNSKYA
CavV                THCNMGCGKEPLQQALHNIDVLQGKSNPQNNMNTHTCDSEEHIYFDSHWYKDGGFKKPSYIFSDINKEHYYKLGTTGLCLYLNSKYA
HanaV               THCNMGCGKEPLKHALHNIDILQGKTNPQNNMNTHTCDAEEHIYFDSHWYKDGGFSKPSYIFSDINKEHYYNLGTTGLVLYLNSKYA
KPhV (KP84_0156)    THCNMGCGKDHLQHALHNIDILQGK-NPQNNMNTHTCDAEEHIYFDSHWYKEGNFTKPSYIFSDINKEHYYNLGTTGLSLYLNSKCA
KPhV (KP84_0192)    THCNMGCGKDHLQHALHNIDILQGK-NPQNNMNTHTCDAEEHIYFDSHWYKEGNFTKPSYIFSDINKEHYYNLGTTGLSLYLNSKCA
KphV (KP84_0344)    THCNMGCGKDHLQHALHNIDILQGK-NPQNNMNTHTCDAEEHIYFDSHWYKEGNFTKPSYIFSDINKEHYYNLGTTGLSLYLNSKCA
BBaV (JKT9876)      THCNMGCGKDRLKRALHNIDILQGKSNPQNDMNTHTCDAAEHIYFDSHWYKEGNFKKPSYIFSDINKEHYYNLGSTGTSLYLNSKYA
BBaV (JKT9891)      THCNMGCGKDRLKRALHNIDILQGKSNPQNDMNTHTCDAAEHIYFDSHWYKEGNFKKPSYIFSDINKEHYYNLGSTGTSLYLNSKYA
BBaV (JKT9853)      THCNMGCGKDRLKRALHNIDILQGKSNPQNDMNTHTCDAAEHIYFDSHWYKEGNFKKPSYIFSDINKEHYYNLGSTGTSLYLNSKYA
BBaV (JKT7774)      THCNMGCGKDRLKRALHNIDILQGKSNPQNDMNTHTCDAAEHIYFDSHWYKEGNFKKPSYIFSDINKEHYYNLGSTGTSLYLNSKYA
KSaV (JKT10701)     THCNMGCGKDRLKHALHNIDILQGKSNPQNNMNTHTCDAEEHIYFDSHWYKEGNFKKPSYIFSDINKEHYYNLGSTGKSLYLNSKYA
NseV                THCNMGCGKEKLSNALHNIDIKQGK-NLHNDMNTHICSSDEHIYFDSHWYKINGFSKPSYIFSDMNKEHYYNLGTTGLCLYLNSKYA
MenoV               THCNMGCGKQPLKQALHNIDINQGKNNPQNNMNTHLCDAQEHIYFDSHWYKTGGFSKPSYIFSDINKEHYYKLGATGLCLYLNSKHA
                    *********: *..******: *** * :*:**** *.: *********** ..* *******:******:**:**   ****** *

HouV (16757)        KYVHEYRTVSGNDVFK-SLYSPYCDLGRKPHQADIEPSCSIPDCIITSNIGERFQTLVCNVHKDQMDIISKISQATKYGYQFIYTGK
HouV (V3872)        KYVHEYRTVSGNDVFK-SLYSPYCDLGRKPHQAEIEPSCSIPDCIITSNIGERFQTLVCNVHKDQMDIISKISQATKYGYQFIYTGK
HouV (16740)        KYVHEYRTVSGNDVFK-SLYSPYCDLGRKPHQAEIEPSCSIPDCIITSNIGERFQTLVCNVHKDQMDIISKISQATKYGYQFIYTGK
HouV (V3982)        KYVHEYRTVSGNDVFK-SLYSPYCDLGRKPHQADIEPSCSIPDCIITSNIGERFQTLVCNVHKDQMDIISKISQATKYGYQFIYTGK
NDiV                KYVHEYRTVSGNDVFK-SLYSPYCDLGRKPHQAEIEPSCSIPDCIITSNIGERFQTLVCNVHKDQMDIISKISQATKYGYQFIYTGK
NgeV (JKT9982)      KYVHEYRTVSGNDVFK-SLYSPYCDLGRKPHQAEIEPSCSIPDCIITSNIGERFQTLVCNVHKDQMDIISKISQATKYGYQFIYTGK
CavV                KYVHEYRTVSGNDVFK-SLYSPYCDLGRKPHQAVIEPSCSIPDCIITSNIGERFQTLVCNVHQDQMELISKIAQATKYGYQFIYTGK
HanaV               KYIHEYRKVSGNDVFK-SLYNPFCDLGREPHQAAIEPSCSIPDCIITSNIDEKFQTLVCNIHKDQMEIISKISQATKYGYQFVYTGK
KPhV (KP84_0156)    KYIHEYRTINGTDVFK-TLYSQYCALERQPHQAAIKPSCSIPDCIITSNIGENFQTLVCNVHKDQMDIICKISQATGYGYQFIYTGK
KPhV (KP84_0192)    KYIHEYRTINGTDVFK-TLYSQYCALERQPHQAAIKPSCSIPDCIITSNIGENFQTLVCNVHKDQMDIICKISQATGYGYQFIYTGK
KphV (KP84_0344)    KYIHEYRTINGTDVFK-TLYSQYCALERQPHQAAIKPSCSIPDCIITSNIGENFQTLVCNVHKDQMDIICKISQATGYGYQFIYTGK
BBaV (JKT9876)      KYIHTYRIVSGNDVFK-TFYAPYCTLERQPHTAVIKSSCSIPDCIITSNIGENFQTLVCNVHHDQMDIISKIAQATRYGYQFIYTGK
BBaV (JKT9891)      KYIHTYRIVSGNDVFK-TFYAPYCTLERQPHTAVIKSSCSIPDCIITSNIGENFQTLVCNVHHDQMDIISKIAQATRYGYQFIYTGK
BBaV (JKT9853)      KYIHTYRIVSGNDVFK-TFYAPYCTLERQPHTAVIKSSCSIPDCIITSNIGENFQTLVCNVHHDQMDIISKIAQATRYGYQFIYTGK
BBaV (JKT7774)      KYIHTYRIVSGNDVFK-TFYAPYCTLERQPHTAVIKSSCSIPDCIITSNIGENFQTLVCNVHHDQMDIISKIAQATRYGYQFIYTGK
KSaV (JKT10701)     KYVHAYRTVSGNDVFK-TLYAQNCSLERQPHKAVIKPSCSIPDCIITSNIGENFQTLVCNIHQDQMDIISKIAQATRYGYQFVYTGK
NseV                KYCHVYQEVTGTDVFK-SLYSSSCELELKPHKAIITDCTSTPDCINSSTLGSQFQTIVCSLHYGQMDIISKISQATQYGYRFVYTG-
MenoV               KYLHAFQPISGNDVFKTEFYNSQCTTNRTPHHLSIEDNTSMPACIIQSSSNEDFHTFICDGHYDQMQIISDISKATKYGYQFIKTGP
                    ** * :: :.*.****  :*   *   **   *    * * **  *. .. *:*::*. * .**::*..*::** ***:*: **

HouV (16757)        TLLNNHAALAKAPHNWDHQKLEIPGYNTRKQHSS-HMTTKALGILHILQDSMLYTNRKTLNPNLPVILPGSASYLGDTVLANEMAKT
HouV (V3872)        TLLNNHAALAKAPHNWDHQKLEIPGYNTRKQHSS-HMTTKALGILHILQDSMLYTNRKTLNPNLPVILPGSASYLGDTVLANEMAKT
HouV (16740)        TLLNNHAALAKAPHNWDHQKLEIPGYNTRKQHSS-HMTTKALGILHILQDSMLYTNRKTLNPNLPVILPGSASYLGDTVLANEMAKT
HouV (V3982)        TLLNNHAALAKAPHNWDHQKLEIPGYNTRKQHSS-HMTTKALGILHILQDSMLYTNRKTLNPNLPVILPGSASYLGDTVLANEMAKT
NDiV                TLLNNHAALSKAPHNWDHLTLEIPGYNTRKQHSS-HMTTKALGILHILQDSMLYTNRKTLNPNLPVILPGSASYLGDTVLANEMAKT
NgeV (JKT9982)      TLLNNHAALSKAPHNWDHLTLEIPGYNTRKQHSS-HMTTKALGILHILQDSMLYTNRKTLNPNLPVILPGSASYLGDTVLANEMAKT
CavV                ILLNNHAALAKAPLDWDHLTLEIPGYNTRKQHSS-HMTTKALGILHILQDSMLYTNRKTLNPNLPVILPGSASYLGDTVLANEMSKT
HanaV               TLLNNHSTLSKAPPTWNHLTLELPGCN--RKHVS-HTTTKASGILYILQDSMLYTNRKTLNPNLPVIIPGAASQFGDTVLTNEISKN
KPhV (KP84_0156)    NLLHNHSALSKAPHNWDHLTLEIPGYNTRKQHSS-HMTTKALGILHILQDSMLYTNRKTLNPKLPVIMPGSASFLGETVLANEMSKY
KPhV (KP84_0192)    NLLHNHSALSKAPHNWDHLTLEIPGYNTRKQHSS-HMTTKALGILHILQDSMLYTNRKTLNPKLPVIMPGSASFLGETVLANEMSKY
KphV (KP84_0344)    HLLHNHSALSKAPHNWDHLTLEIPGYNTRKQHSS-HMTTKALGILHILQDSMLYTNRKTLNPKLPVIMPGSASFLGETVLANEMSKY
BBaV (JKT9876)      TLLNNHSALSKAPPDWDHLKLEIPGYNTRKQHSS-HMTTKALGILHILQDSMLYTNRKTLNPNLPVIMPGSASFLGETILANEMSKQ
BBaV (JKT9891)      TLLNNHSALSKAPPDWDHLKLEIPGYNTRKQHSS-HMTTKALGILHILQDSMLYTNRKTLNPNLPVIMPGSASFLGETILANEMSKQ
BBaV (JKT9853)      TLLNNHSALSKAPPDWDHLKLEIPGYNTRKQHSS-HMTTKALGILHILQDSMLYTNRKTLNPNLPVIMPGSASFLGETILANEMSKQ
BBaV (JKT7774)      TLLNNHSALSKAPPDWDHLKLEIPGYNTRKQHSS-HMTTKALGILHILQDSMLYTNRKTLNPNLPVIMPGSASFLGETILANEMSKQ
KSaV (JKT10701)     TLLNNHSALSKAPPNWDHLTLEIPGYNTRKQHSS-HMTTKALGILHILQDSMLYTNRKTLNPNLPVIMPGSASFLGETVLANEMSKQ
NseV                SELNNKAHLNLAPHTWDHQTLEIPGYSTRINNPC-HMQTKAIGVLRILQDSILYTPRKTLNAKYPVILPGSASNYGETILANEFRRH
MenoV               TTLSNHSNLQKAPSNWNHTQLLIPGYETRKISPPGHMLNKALGILSILQDSMLYTHRTTLNKNLPIIHPGAAGFNGDTILANEFRKY
                      * *:: *  **  *:*  * :** .        *  .** *:* *****:*** *.*** : *:* **:*.  *:*:*:**: :

HouV (16757)        LKQTKFIHIDPRLKIDNNTTHHRKTLMEMLDIGYTTELIISDIHDNKN-PWIPELMTYTLKYLIDTGTLIMKITSRGATEDVIQQLE
HouV (V3872)        LKQTKFIHIDPRLKIDNNTTHHRKTLMEMLDIGYTTELIISDIHDNKN-PWIPELMTYTLKYLIDTGTLIMKITSRGATEDVIQQLE
HouV (16740)        LKQTKFIHIDPRLKIDNNTTHHRKTLMEMLDIGYTTELIISDIHDNKN-PWIPELMTYTLKYLIDTGTLIMKITSRGATEDVIQQLE
HouV (V3982)        LKQTKFIHIDPRLKIDNNTTHHRKTLMEMLDIGYTTELIISDIHDNKN-PWIPELMTYTLKYLIDTGTLIMKITSRGATEDVIQQLE
NDiV                LKQTKFIHIDPRLKIDNNTTHHRKTLMEMLDIGYTTELIISDIHDNKN-PWIPELMTYTLKYLVDTGTLIMKITSRGATEDVLQQLE
NgeV (JKT9982)      LKQTKFIHIDPRLKIDNNTTHHRKTLMEMLDIGYTTELIISDIHDNKN-PWIPELMTYTLKYLIDTGTLIMKITSRGATEDVIQQLE
CavV                LKQTKFVHIDPRLKIDNNTTHHRKTLMEMLDIGYTTELIISDIHDNNN-PWIPELMEYTLKYLIDTGTLIMKITSRGATEAVLQQLE
HanaV               LKRTKFINVDPRLKLDNHNTHYRKPLKEMLDIGYTTELIISDIHDNNNNTWIHELIEYTLKYLIETGTLIMKITSRAATEDTLEYLE
KPhV (KP84_0156)    LKQTKFVHIDPRLKIDNQTTHYRKTLMEMLDIGYPTELIISDIHNNTS-PWIPELVSYTQKYLIDTGTLIMKITSRGATEDNLALLE
KPhV (KP84_0192)    LKQTKFVHIDPRLKIDNQTTHYRKTLMEMLDIGYPTELIISDIHNNTS-PWIPELVSYTQKYLIDTGTLIMKITSRGATEDNLALLE
KphV (KP84_0344)    LKQTKFVHIDPRLKIDNQTTHYRKTLMEMLDIGYPTELIISDIHNNTS-PWIPELVSYTQKYLIDTGTLIMKITSRGATEDNLALLE
BBaV (JKT9876)      LKRSKFIHIDPRLKIDNQSTHYRKTLMEMLDIGYPTELIISDIHNNTS-PWIPELISYTQKYLVDNGTLIMKITSRAATADNLELLE
```

```
BBaV (JKT9891)    LKRSKFIHIDPRLKIDNQSTHYRKTLMEMLDIGYPTELIISDIHNNTS-PWIPELISYTQKYLVDNGTLIMKITSRAATADNLELLE
BBaV (JKT9853)    LKRSKFIHIDPRLKIDNQSTHYRKTLMEMLDIGYPTELIISDIHNNTS-PWIPELISYTQKYLVDNGTLIMKITSRAATADNLELLE
BBaV (JKT7774)    LKRSKFIHIDPRLKIDNQSTHYRKTLMEMLDIGYPTELIISDIHNNTS-PWIPELISYTQKYLVDNGTLIMKITSRAATADNLELLE
KSaV (JKT10701)   LKRSKFIHIDPRLKIDNQSTHYRKTLMEMLDIGYPTELIISDIHNNTS-PWIPELILYTQKYLVDNGTLIMKITSRGATEDNLKLLE
NseV              LKQSKFIHIDPRLKLDNNTTHFKKTLKDMLPIGYPTELIISDIHNNDDPTWITELIDYTHKYLLDTGTLIMKITSRGATSTALEQLE
MenoV             LKQSKIINIDPRLKR-NDDTHYKLLLKDMLPKGFNTELIISDIHSDVE--WIPELIEYTNKYLQQSGTLIMKITSRMATEKAIQQLE
                  **::*::::*****  *. **.:  * :**  *: *********.: .  ** **: ** *** :.********** **   :  **
HouV (16757)      DLSKNFTYVRVCNLNAVTFSSELWIVFANKRKPPVQGWTSHELRAELRKHWYSMTRSIIQPLMRSRQSVFRYSPK
HouV (V3872)      DLSKNFTYVRVCNLNAVTFSSELWIVFANKRKPPVQGWTSHELRAELRKHWYSMTRSIIQPLMRSRQSVFRYSPK
HouV (16740)      DLSKNFTYVRVCNLNAVTFSSELWIVFANKRKPPVQGWTSHELRAELRKHWYSMTRSIIQPLMRSRQSVFRYSPK
HouV (V3982)      DLSKNFTYVRVCNLNAVTFSSELWIVFANKRKPPVQGWTSHELRAELRKHWYSMTRSIIQPLMRSRQSVFRYSPK
NDiV              DLSKNFTYVRVCNLNAVTFSSELWIVFANKRKPPVQGWTSHELRAELRKHWYSMTRSIIQPLMRSRQSVFRYSPK
NgeV (JKT9982)    DLSKNFTYVRVCNLNAVTFSSELWIVFANKRKPPVQGWTSHELRAELRKHWYSMTRSIIQPLMRSRQSVFRYSPK
CavV              HMAKNFTYVRVCNLNAVTFSSELWIVFANKRKPPVQGWTSHELRAELRKHWYSMTRSIIQPLMRARQSVFRYSPK
HanaV             HLSKNFTYVRVCNLNAVTPSSVLWIVFADKRKPPVLGWTSHELRNELRKHWYSMARNIIQPLTRARPCIFRYSPK
KPhV (KP84_0156)  NLSKDFTYVRVCNLNAVTYSSELWIVFADKRKPPVQGWTSHELKSELRKHWYAMTRNIIQPIMRSRTCVFRYSPK
KPhV (KP84_0192)  NLSKDFTYVRVCNLNAVTYSSELWIVFADKRKPPVQGWTSHELKSELRKHWYAMTRNIIQPIMRSRTCVFRYSPK
KphV (KP84_0344)  NLSKDFTYVRVCNLNAVTYSSELWIVFADKRKPPVQGWTSHELKSELRKHWYAMTRNIIQPIMRSRTCVFRYSPK
BBaV (JKT9876)    NLSKDFTYVRVCNLNAVTYSSELWIVFADKRKPPVQGWTSHELKSELRKHWYSMTRGIIQPTMRSRICVFRYSPK
BBaV (JKT9891)    NLSKDFTYVRVCNLNAVTYSSELWIVFADKRKPPVQGWTSHELKSELRKHWYSMTRGIIQPTMRSRICVFRYSPK
BBaV (JKT9853)    NLSKDFTYVRVCNLNAVTYSSELWIVFADKRKPPVQGWTSHELKSELRKHWYSMTRGIIQPTMRSRICVFRYSPK
BBaV (JKT7774)    NLSKDFTYVRVCNLNAVTYSSELWIVFADKRKPPVQGWTSHELKSELRKHWYSMTRGIIQPTMRSRICVFRYSPK
KSaV (JKT10701)   NLSKDFTYVRVCNLNAVTYSSELWIVFADKRKPPVQGWTSHELRSELRKHWYSMTRNIIQPTMRSRTCVFRYSPK
NseV              NLSRIFTYVRVCNVNAVTCSSELWIVFADKRKPTVKGWTSHDVRSELRKHWYSMTQNIKNPILRYRLDIFRYSPK
MenoV             TLSENFTYVRVCNLNVVTCSSELWIVFANNRKPPVNGWTSHDLRNELRKHWYSMTHNILHPTIRSRLDVFRYSPK
                   ::. ********:*.** ** ******::***.* *****::: *******:*::.* :*  * *  :******
```
