# Supplementary material for: Mesoniviruses are mosquito-specific viruses with extensive geographic distribution and host range
Source: Virol J. 2014 May 20;11:97. doi: 10.1186/1743-422X-11-97 (PMC4038087; doi:10.1186/1743-422X-11-97)
Supplement: Additional file 3: Figure S3 — A Clustal X multiple sequence alignment of the polypeptides encoded in ORF2a (S proteins) of the mesoniviruses. A predicted signal peptide in MenoV and predicted transmembrane domains in all mesoniviruses are shaded in aqua, predicted N-glycosylation sites are shaded in green, cysteine residues are shaded in yellow and the sites of proteolytic cleavage to generate glycoproteins S1 and S2 and the unidentified N-terminal fragment are shaded in purple. [file 1743-422X-11-97-S3.pdf]

Supplementary Figure S3. A Clustal X multiple sequence alignment of the polypeptides encoded in ORF2a (S proteins) of the mesoniviruses. A predicted signal peptide in MenoV and predicted transmembrane domains in all mesoniviruses are shaded in aqua, predicted N-glycosylation sites are shaded in green, cysteine residues are shaded in yellow and the sites of proteolytic cleavage to generate glycoproteins S1 and S2 and the unidentified N-terminal fragment are shaded in purple.

```
HouV (V3982)         MINS-KCPLLFQTTTTSMPNQARRNRSGPPKILKTEMQWHQNHNANASS------SSKLHRSPLDNHPQ-----DNQNLNATNL
NDiV                 MINS-KCPLLFQTTTTSMPNQAHRNRPGPPIILKPETQWHQNHNANASS------SSKLHRSPLNNHPK-----DNQNLNATNL
NgeV (JKT_9982)      MINS-KCPLLFQTTTTSKPNQAHRNRPGPPIILKPETQWHQNHNANASS------SSKLHRSPLNNHPQ-----DNQNLNATNL
CavV                 MINS-ICQLQFQTPTTPMPNQALRNKTGPPKILKPEMQWHQNHNANANSR----SSSNQHSNLNNYHHQ-----ERQNKNATNL
KPhV (KP84_156)      MINS-KCQLTLRLPITQQPNKAPRS-PDNLKILTTVMPRLQNPNAKANSKTTLSSKTRLNSRINNNKNSRTVPISKQNHDVLSP
HanaV                MISS-QCQLLPTTRTTTKPSQAPHVKISSQKPLT-EMQWLQNRNASANKT--------IQQHHQNKHPQ-------VNLNKIAT
BBaV (JKT_9876)      MINSTKCPLTLRTPTTLMPIKAPRSKTVHHQTLITEMPRHQNLNAKASSRTNRLINANPISKTNSKNSKTNKVKPQQANLKRSV
KSaV (JKT_10701)     MINSTKCPLTLRTPTTSMPNKAPRSKTVHHQTLITEMPRHQNLSAKASSRINRLTKTSLNSKTNSKNNKTSKINLQQVSLRQSV
NseV                 MITS-PCRLQFHKIQLKLAPRNKANKTTQTTEQTNPQCHSDAILIETLG--------THNNQMLVRVCALNRIEQQAKPELVKH
MenoV                MITL-KCLLLITHLMLETAQASLSKINNLIRGFSKTPNKSVNLSTPFLA--------IEMGKPEAPIINQLRISQQTNPLLSNL
                     **.   * *         .

HouV (V3982)         MLQHLPSLRSRKQLQQAPTTPKPAVNFTKSEKNSMLETTWDGGKMKRLDQQSSSSLNLKWHPELTKSIIAINLRILLTISSILS
NDiV                 MLQHLPSLRSRKQLQQAPTTPKPAVNFTKSEKNSMLETTWDGGKMKRLDQQSSSSLNLKWHPELTKSTIAINLR-ILTISSILS
NgeV (JKT_9982)      MLQHLPSLRSRKQLQQAPTTPKPAVNFTKSEKNSMLETTWDGGKMKRLDQQSSSSLNLKWHPELTKSTIAINLR-ILTISSILS
CavV                 MLQHQRSLRLRKQLQQVPTTLKPMVNSIKSEKNSMLETIWDGGKMKRLAQQSSFYSNPKWHHELTKSTTVINLK-TLTTTSILS
KPhV (KP84_156)      ILHKICSSRLRNPLRQAPTTPKPMVNSTKLEKNSMLETIWDGERMKRLDPQSSSSLNLKWHPELTKSITVINSK-ILTISSILS
HanaV                HLRPHNSLRSRKQLQQAPTTPKLMENCTKSEKNSMQETIWDGDVMKRLDQPSSSSLNQKWLPELTRCTTVINLK-MQTITSIHL
BBaV (JKT_9876)      NQITMQSSQKLKSLHLAPTSPNPMVSATKSDLNITHETSWDGERMKRLDQVSSSSSSQKWYPALTKSTIGVKLK-EQTVFSILS
KSaV (JKT_10701)     NRTIQQSNQKLRNLQSVPTTPNPMVSATRSDLNIMHETTWDGERTKRLDQVSSSSSNQKWYPGLIKSTIGVNLK-EQTVISILS
NseV                 QINPRPTRKQRKQLPQAQITPKPMVSDIKSEKNSMLATIWDGDAMRKQDHQSSSSLNQKWLPELTRFITAVNLR-MLTIVSILS
MenoV                MVNPSS------QPQLVPTTQRPKESFTKLEPSLMLETTWDGDVTRKMDPLYNSSSSQKWPQELTKLTTESKMK-MTANMSKHL
                                     .  : .   .  : : .    * ***   ::     .   . **   * :     : :   :  *

HouV (V3982)         VLAYLFKTQPLSAMQFTTTKNSLLKKKMSMFVNYLTPSMQSSCVHVKHLTLVPFLLLLLMLP-NANCSTRIDLSTHHIVSYNKP
NDiV                 VLAYLFKTQPLSAMQFTTTKNSLLKKKMSTFVNYLMPSMQSSCVHVKHLTLVPFLLLLLMLP-NANCSTRIDLSTHHIVSYNKP
NgeV (JKT_9982)      VLAYLFKTQPLSAMQFTTIKNSLLKKKMSMFVNYLTPSMQSSCVHVKHLTLVPFLLLLLMLP-NANCSTRIDLSTHHIVSYNKP
CavV                 VLAYLFKIQPLSAMQFTTIPNSLPKKKMSTFVNYPTPSMQSSCVHVKPLTLVHFLLLLLMLP-NAHCSTRIDLSTHHIVSYNKP
KPhV (KP84_156)      VLEYLFKIQPLSVMQFSITKNSPMKKRMSTYENYQMPSMQSSCALAKLLVLVPFLLLLLMLP-SVNCSTRIDLSTHHIVSYNKP
HanaV                VLAYLFKIQPLNAMQFTIMQNSLLKKRMSMFVNYQMPSMQFSCVRAKLLIPMLCLISLLTLP-NVECSTRIDLSTHHIVSYNKP
BBaV (JKT_9876)      VLVYLFKTQPSNATQSSTMQTSRTTRSPTTSKTYLTHSMQFFSAHAMPLNLVPYLLLLLMLN-QANCSTRIDLSTHHIVSYNKP
KSaV (JKT_10701)     VLAYLFRTQNLNETQSLTTQTSRTTRSQNTFINYLMHSMQSFSVHATPFNLAPYLLLLLMLNQQANCATRIDLSTHHIVSYNKP
NseV                 GLGSLFKILLLSAMRSLNIQLFQRRKRENTYVNSPMPSTPSYCAHATPSKLQLSLLLLLTLS-SVTCLTRIDLSTHHIVSYNKP
MenoV                RSASLSKIPPLLAMPSSLPKTSNQMKRMPTYSTFLTHSTQSSLAPRLHSILIHSLLLLSMLN-TTHSLTRIDLKTHHIVSYNKP
                         * :                  :      .    *     .          *: *  *   . . *****.**********

HouV (V3982)         LIVVDDFLKTTLKYNFGTDLYNSAINYKTSFEQLLNNFKTPYQPLVDAFRVLFSYLGIEPVAHPFKDYYDADSPCPLQTTTT-T
NDiV                 LIVVDDFLKTTLKYNFGTDLYNSAINYKTSFEQLLNNFKTPYQPLVDAFRVLFSYLGIEPVAHPFKDYFNADSPCPLQTTTT-T
NgeV (JKT_9982)      LIVVDDFLKTTLKYNFGTDLYNSAINYKTSFEQLLNNFKTPYQPLVDAFRVLFSYLGIEPVAHPFKDYFNADSPCPLQTTTT-T
CavV                 LIAVDDFLKTTLKYNFGTDLYNSAINYKTSFEQLLNNFKTPYQPLVDAFRVLFSYLGIEPVAHPFKDYLNADSPCPLHTKSS-T
KPhV (KP84_156)      LIVVDDFLKTTLKYNFGTDLYNSAINYKTSFEQLLNNFKTPYQPLVDAFRVLFSYLGIEPVAHPFKDYLNADSACPLQTTTN-T
HanaV                LIVVDDFLKTTLKYNFGTDLYNSAINYKTSFEQLLNNFKTPYQPLVDAFRVLFSYLGIQPVAHPFNNYLNADSPCPLQTKTT-S
BBaV (JKT_9876)      LIVVDDFLKTTLKYNFGTDLYNSAINYKTSFELLLNNFKTPYQPLVDAFYILFSYLGITPVSHPFRDYLFETSPCPLKITTT-T
KSaV (JKT_10701)     LIVVDDFLKTTLKYNFGTDLYNSAINYKTSFELLLNNFKTPYQPLVDAFHILFSYLGITPVSHPFRDYLYADSPCPLKITTT-T
NseV                 LIVVDDFLKTTLRYNFGSDLYKSAINYKTSFEQLLNSFRTPYQPLVEAFHVLFGYLGIQPVKHPFNDYLNADSPCPLQTTTMPA
MenoV                LIAVDDFLKTTLKYNFGTDLYSSAIKYKESFEQLALNFRTPYQPLIEAFRVLFGYLGITPVTHPFSIFLYPTSPCPIEVTQP-G
                     **.*********:****:***.***:** *** *  .*:***:**::** :**.**** ** ***  :    *.**:. .

HouV (V3982)         GDVTTIGEHFQEILEDGNLELEPLASYWLRHTEDIFVYTRSQLWAFICPSEFAQASIFLPNYTEAIYNVSTTFCKTVYYDTTTN
NDiV                 GDVTTIGEHFQEILDDGNLELEPLASYWLRHTEDIFVYTRSQLWAFICPSEFAQASIFLPNYTEAIYNVSTAFCKTVYYDTPTN
NgeV (JKT_9982)      GDVTTIGEHFQEILDDGNLELEPLASYWLRHTEDIFVYTRSQLWAFICPSEFAQASIFLPNYTEAIYNVSTAFCKTVYYDTPTN
CavV                 GSLT-IGEHFQKVLDDGEFELEPLASYWLRHTEDISIYTRSQLWAFICPSEFAQASIYLPTYTEAIFKVATTFCKTVTYDTTKN
KPhV (KP84_156)      GDVTTIGEHFQEILDDGDLELEPLASYWLRHTEDIFVYTRSQLWAFICPSEFAQASIFLPNYTEHIYNSSTTFCKTVYYDTTIN
HanaV                GDVTTIGEHFQEILDDGNFQLEPLASYWLRHTEDIFMYTRSQLWAFICPSEFAQASIFLPNYTEAIYNVSITFCKDVHYDTAIN
BBaV (JKT_9876)      SNVTTIGAHFTKILEDGDLAMEPLASYWLRHTDDIHVYTRSQLWAFICPSEFAPASIFLPDYTPTIYNIATSFCKAISYDNTLS
KSaV (JKT_10701)     GDVTTIGAHFQKILEDGDLKMEPLASYWLRHTEEIYVYTRSHLWAFICPSEFAPASIFLPNYTPAIYNVATSFCKSVSYDNSLN
NseV                 GDVTTIGEHFQSILDDGNLQLEPLASYWLRHTEDIFVYTRSQLWAFICPTEYAQANMFLPQYTDAIYNASLAFCKDVIYNTTIN
MenoV                SPIQTIGQIFEEIITEGSYEYEALASYWLTHIEDLKFLTRQQVWAFICPSEFAQASIFLPNYSESIYNSSIAFCNSVGYDLKIS
                     . :  **  * .:: :*.   *.****** * ::: . **.::******:*:* *.::** *:  *:: : :**: : *:   .

HouV (V3982)         AFN---AEICNKVNFITPAKANKRSKRWDSSYVCGWPLVSSAAKVLGGECTTNIDIGSLKSSLTAIQNFSYANTELIHDLQSQL
NDiV                 AFN---AEICNKVNFITPAKAQKRSKRWDSSYVCGWPLVSSAAKVLGGECTTNIDIGTLKSSLNAIQNFSYANTELIHDLQSQL
NgeV (JKT_9982)      AFN---AEICNKVNFITPAKAQKRSKRWDSSYVCGWPLVSSAAKVLGGECTTNIDIGSLKSSLTAIQNFSYANTELIHDLQSQL
CavV                 AQN---AEICNKVNFITPEKALKRNKRWDSSYVCGWPLVSSAAKVLGGECTTNIDIGSLKSSLTAIQNFSYANTELIHDLQSQL
KPhV (KP84_156)      AFN---AEICNKVNFIVPTKSQKRTKRWDSSYVCGWPLVSSAAKVLGGECTTNIDIGSLKSSLTAIQNFSYANTELIHDLQSQL
HanaV                DFN---AEICNKVNFITPEKAQKRHKRWDSSYVCGWPLVSSAAKVLGGECTTNIDIGSLKSSLTAIQNFSYANSELIHDLQSQL
BBaV (JKT_9876)      DPE---AEVCNKVNFITPKRAQSRRKRWDSSYVCGWPLVSSTAKLLGGECTTNIDIGSLKSSLTAIQNFSAQNSELINDLQSQL
KSaV (JKT_10701)     NPD---AEVCNKVNFITPELAQSRRKRWDSSYVCGWPLVSSTAKLLGGECTTNIDIGSLKSSLTAIQNFSSQNTELINDLQSQL
```

```
NseV                SFN---GEICDKVNFITPTQSKIRKRRWDSSYVCGWPLVSSAAKVFGGECTTNIDVDSLKSSLTSMQQFSNANSELIQNLQSQL
MenoV               SYNDTSYQVCNKVNFLVLKAENRRQKRWDSSYVCGWPLVSSAAKVLGGECTTNIDVDSLKSNLHAIQNFSLQNTELIHNLQEQL
                      :    ::*:****:.      * :***************:**::*********:.:***.*  ::*:**   *:***::**.**

HouV (V3982)        SVVNARTNLHYNQLQQLVTAINDHQAKYVNDINNLINHIKNTTNTMENRININSIIMSYTNSLFRVYQNIVDYRFAYIETLSS
NDiV                SVVNARTNLHYNQLQQLVTAINDHQAKYVNDINNLINHIKNTTNTMENRINVNS-IIMSYTNSLFRVYQNIVDYRFAYIETLSS
NgeV (JKT_9982)     SVVNARTNLHYNQLQQLVTAINDHQAKYVNDINNLINHIKNTTNTMENRINVNS-IIMSYTNSLFRVYQNIVDYRFAYIETLSS
CavV                SVVNARTNLHYNQLQQLVTAINDHQAKYVTDINNLINHIENTTNTLENRINVNS-IIMSYTNSLFRVYQNIVDYRFAYIETLSS
KPhV (KP84_156)     SVVNARTNLHYNQLKQLVSAINDHQAKYVNDINNLINHIQNTTNTLENRINVNS-IIMSYTNSLFRVYQNIVDYRFAYIETLSS
HanaV               SVVNARTNLHYNQLQQLVTAINEHQIKYVNDINHLISEIKNTTNTLESRIAINS-IIMSYTNSLFRVYQNIVDYRFAYIETLSS
BBaV (JKT_9876)     SVVNARTNLHYNQLQQLVTDINEHQIKYVTDINNLISQIRNNTNRMKARVSINS-IIMSYTNSLFRVYQNIVDYRFAYIETLSS
KSaV (JKT_10701)    SVVNARTNLHYNQLQQLVTAINEHQSKYVSDINNLISQIRNNTNTMKARVSINS-IIMSYTNSLFRIYQNIVDYRFAYIETLSS
NseV                TIVNARTNLHYKQLQQLVTAINSHQERYISDINNLVEQVKNTTNSLESRISINS-IISSYTSSLFRVYQTIVDYRFAYIETLNS
MenoV               TIVNARTNLHYNQLLQLVNDINNHQARYIADINNLIEQVRNTTNVQTSQININS-IILSYTNSLFRVYQTIVDYRFSYIETLNS
                    ::*********:** ***. **.** :*: ***:*:..:.*.**    :: :** ** ***.****:**.******:*****.*

HouV (V3982)        IQQHYHFPSEHLHAFNVPLQAKLREHGFSIPIIDSNIPYSYGKVRYLNVTGINFYDLEFDIYIPVIKLIHEKDSNYYHSTLSAL
NDiV                IQQHYHFPSEHLHAFNVPLQAKLREHGFSIPIIDSNIPYSYGKVRYLNVTGINFYDLEFDIYIPVIKLIHEKDSKYYHSTLSAL
NgeV (JKT_9982)     IQQHYHFPSEHLHAFNVPLQAKLREHGFSIPIIDSNIPYSYGKVRYLNVTGINFYDLEFDIYIPVIKLIHEKDSNYYHSTLSAL
CavV                IQQHYHFPSEHLHAFNSPLQAKLKEHGFSIPIIDTNVPYSYGKVRYLNVTGINFYDLEFDIYIPVIKLIHEKDSKYFHSTLSAL
KPhV (KP84_156)     IQQHYHFPSEHLHAFNIPLQSKLKEHGFKIPIIESNIPYSYGKVRYLNVTGINFYDLEFDIYIPVIKLIHEKDSNFYHSTLSAL
HanaV               IQQHYHFPSEHLHAFNRPLLDKLKANGFSIPIIDSNIPYSYGKVRYLNVTGINFYDLEFDIYIPVIKLIHEKDSNYYHSTLSAL
BBaV (JKT_9876)     IQEHYHFPSEHLQAFNIPLLAKLREHGFSIPMINSNIPYSYGKVKYINVSGINFYDLEFDIYIPVIKLPHENDSSYFHSTLSAL
KSaV (JKT_10701)    IQEHYHFPSEHLQAFNIPPLTKLREHGFSIPMINNNIPYSYGKVRYINVTGINFYDLEFDIYIPVIKLQHENDSSYFHSTLSAL
NseV                IQQHYHFPSEHMRSFNSDLINKLKAFGFTIPVIDSNIPYSYGKVNYLNVTGINFYDLEFDIYIPITKLSHVNNDNFYYSTLSAL
MenoV               IQQHYHFPSEHMHSFNQELLDKLKAYGFTIPIIGNYIPYSYGVVRYLNVTGINFYDLEFDVYIPITRIHYEYDTKYWHSTLSAL
                    **:********:::**     **:  **.**:* . :***** *.*:**:*********:***: :: :  : .:::******

HouV (V3982)        PIGINTTLVTYNTYQGNAICTDTYCLESPINGFCREGESYWYCGQHYIRTLHKITSLYTKPTKFTESAMFIPPHTMYFVHNTTY
NDiV                PIGINTTLVTYNTYQGNAICTDTYCLESPINGFCREGESYWYCGQHYIRTLHKITSLYTKPTKFTXSAMFIPPHTMYFVHNTTY
NgeV (JKT_9982)     PIGINTTLVTYNTYQGNAICTDTYCLESPINGFCREGESYWYCGQHYIRTLHKITSLYTKPTKFTESAMFIPPHTMYFVHNTTY
CavV                PIGINNTLVTYNTYQGNAICTDTYCLESPINGFCREGESYWYCGQHYIRTLHKITSLYTRPTKFTDSAMFIPPHTMYFVRNTTY
KPhV (KP84_156)     PIGINTSLVTYNTYQGNAICTDTYCLESPISGFCREGESYWYCGQHYIRTLHKITSLYTKPTKFTESAMFIPPHTMYFVHNTTY
HanaV               PIGTNDSLVTYNTYQGNAICTDTYCLESPITGFCREGESYWYCGQHYIRTLHKITSLYTKPTKFTDSAMFIPPHTMYFVHNTTY
BBaV (JKT_9876)     PVGINNSLVTYNTYQGNAICTDTYCLESPVNGFCHEGEGYWYCGRHYIKTLHKITSLYTMPTKFAESAMFIPPHTMYFVHNTSY
KSaV (JKT_10701)    PVGINNTLVTYNTYQGNAICTDTYCLESPINGFCREGEGYWYCGRHYIKTLHKITSLYTRPTKFAERAMFIPPHTMYFVHNTSY
NseV                PIGINSTHVTYNNYQGSAICTDLYCLETPINGFCREGGSYWYCGQYYVDTLHKISKLYTSPTEYSAAAMFIPPHTMYFVKNTTY
MenoV               PIGINLTHVTYNQYQGSAICTDLYCLETPLTGFCREGENYWYCGQHYLKTLHRITNLYTNPTKNVQQAMFIPPHTVYFIRNTSY
                    *:* * : **** ***.***** ****:*:.***:** .*****::*: ***:*:.*** **:    ********:**::**:*

HouV (V3982)        SLNYG-ASLQALAGSILMLTCNSTVQIPGYSFNANDFVSCTDMNVNNVFIHPSLRVNDANFYIPPTRVDLLEKLYKRDIIPILN
NDiV                SLNYG-SSLQALAGSILMLTCNSTVQIPGYNFNSNDFVSCTDMNVNNVFIHPSLRVNDANFYIPPTRVDLLEKLYKRDIIPILN
NgeV (JKT_9982)     SLNYG-SSLQALAGSILMLTCNSTVQIPGYSFNSKDFVSCTDMNVNNVFIHPSLRVNDANFYIPPTRVDLLEKLYKRDIIPILN
CavV                SLNHG-SSLQALAGSVLMLTCNSTVQIPGYSFNANDFVSCTDMNVNNVFIHPSLRVNDANFYIPPTRVDLLEKLYKRDITPILK
KPhV (KP84_156)     SLNYG-SSLQALAGSILMLTCNSTVQIPGYSFNANDFVSCTDMNVNNVFIHPSLRVNDANFYIPPTRVDLLEKLYKRDITPILK
HanaV               SLNYG-SSLHALAGSVLMLTCNSTVQIPGYSFNANDFVSCTDMNVNNVFIHPSLRVNDTNFYIAPTQVDVLEKLYKRDVLPILK
BBaV (JKT_9876)     TINHG-SSLQALAGSVLMLNCNSTVQISGYSFNSSDFVSCTTMNENNVFIHPSLRQSDENFYIPPTRVDLLEKLYTRDVLPIIK
KSaV (JKT_10701)    TIDNG-NSLQALAGSVLMLTCSSTVQISGYSFNASDFVSCTTMNENNAFIHPSLRSSDEHFYIPPTRVDLLEKLYTRDVLPIIK
NseV                TLNEN-NIYTALTGSVLMLNCNSTVLVGRNYFNTSDFVECTNLNKNNVFIHNRLNTTDPDFYIGPSEINKLENYYLRDIDPIIK
MenoV               SLNHGPSNSTGLTGSVMMLTCNSSIQIPGYVFNAADFVSCSDITNNNVFIHGGLHPDDPNFYTFPSQVQQLEEFYNRDITPIIT
                    ::: .     .*:**::**.*.*:: :    **: ***.*: :. **.***  *.  * .**  *:.:: **: * **: **:.

HouV (V3982)        HIQKANDITIDTTADEELKQQYETLKNDFNAKYDALNIENRRIHALINSMHSIQSEPSYILYMVIAVIVFIVLKFLRII
NDiV                HIQKANDITIDTTADEELKQQYETLKSDFNAKYDALNIENRRIHALINSMHSIQSEPSYILYMVIAVIVFIVLKFLRII
NgeV (JKT_9982)     HIQKANDITIDTTADEELKQQYETLKSDFNAKYDALNIENRRIHALINSMHSIQSEPSYMLYMVIAVIVFIVLKFLRII
CavV                HIHKADDITIDTTSDEELKQQYETLKNDVNAKYDALIIENRRIHALINSMHSIQPEASYVLYLVIAVIVFIVLKFLRII
KPhV (KP84_156)     HVQ-ANDLVIDTTADEELKQQYETLKNDFNAKYDALNIENRRIHALINSMHSIKSEPSYFLYMVIAVIVFIVLKFLRIL
HanaV               HIRQYDSIKVDTTADKELQQQYETLKNDFNAKYEALIAENSRIYTLINSMHSINSESSYMLYLVIAVIIFIVLKFLRII
BBaV (JKT_9876)     DIQQDRSITIDTTDDEQLQQQYETLKNDFNAKYAALNIENKRIYALINSMHSIHNETSYVLYLVIAIIVFIVLKFIRLI
KSaV (JKT_10701)    DITQDQSITIDTTDDEQLQQQYETLKNDFNAKYAALNIENKRIYALINNMHSIHSETSYVLYLVIAIIVFIVLKFIRII
NseV                HINNTVKLRIDTSSDEILNQQYATLKDSLETKIKLLASENQRIIATIGNMHAIPSETPMFFYIILGVLLLFVLKLFRII
MenoV               NIHNYQDLFIDSSQDKNLTQQYETLRNDFDMKYKKLLLENQRILQLINNMHSIDSETPIYFYIIIGVLVLFILKLFRII
                    .:    .: : :: *: * *** **:...: *   *  ** **   *..**:*  *..  :*:::.:::::**::*::
```
