# Supplementary material for: Mesoniviruses are mosquito-specific viruses with extensive geographic distribution and host range
Source: Virol J. 2014 May 20;11:97. doi: 10.1186/1743-422X-11-97 (PMC4038087; doi:10.1186/1743-422X-11-97)
Supplement: Additional file 4: Figure S4 — A Clustal X multiple alignment of the sequences of putative polypeptides encoded on ORF4 which occurs in the 3’-terminal regions of all mesoniviruses except MenoV. To emphasize the alignment, the KSaV ORF4 protein has been shown to commence at the next available methionine residue located 34 amino acids downstream of the predicted initiation codon. [file 1743-422X-11-97-S4.pdf]

Supplementary Figure S4. A Clustal X multiple alignment of the sequences of putative polypeptides encoded on ORF4 which occurs in the 3’-terminal regions of all mesoniviruses except MenoV. To emphasize the alignment, the KSaV ORF4 protein has been shown to commence at the next available methionine residue located 34 amino acids downstream of the predicted initiation codon.

```
KSaV (JKT_10701)   MLDSTLDLVVGRKRSAHVYVSGPRPNLGFLYIR--TQGLEQADHSSKITIYF-----------
BBaV (JKT_9876)    MLESTLDLVVGRKRSAHVYVSGPRPNPRFLYIR--TQGLEQADHSSKITSTFDKVEPLNETNV
NgeV (JKT_9982)    MLESTLDLVVGRKRSAHVYVSGLGPNRRFLYIR--TQGLEQADHSSK----------------
NDiV               MLESTLDLVVGRKRSAHVYVSGLGPNRRFLYIR--TQGLEQADHSSK----------------
HouV (V3892)       MLDSMLDLVVGRKRSAHVYVSGLGPNLRFLYIR--TQGLEQANHSCKLTSL------------
KPhV (KP84_156)    MLESTLDLVVGRKRSAHVYVSGPRPNRRFLCIR--TQGLEQAEHSSK----------------
CavV               MLESMLDLVVGRKRSAHVYVSGLGPNRRFLYIR--TQGLEQADHSCKITSL------------
HanaV              MLESMLDLVVGRKRIAHVYVSGLGPNRRFLYIR--TQGLEQADHSCKLTSLEQSCTTE-----
NseV               ML----DLVVGRKYSAHVYVSGLGPNRRFLYIRYARPGTSRTFKLNNITLEES--SPLNENNV
                   **    *******  *******  **  ** **    * .:: :  :
```
