# Supplementary material for: Mesoniviruses are mosquito-specific viruses with extensive geographic distribution and host range
Source: Virol J. 2014 May 20;11:97. doi: 10.1186/1743-422X-11-97 (PMC4038087; doi:10.1186/1743-422X-11-97)
Supplement: Additional file 5: Figure S5 — A Clustal X multiple sequence alignment of region immediately downstream of the ORF1a/ORF1b RFS that has been predicted previously to adopt a stem-loop structure in NDiV. The alignment illustrates sequence variations in nucleotides predicted in NDiV to be involved in base pairs that sustain the structure. [file 1743-422X-11-97-S5.pdf]

Supplementary Figure S5. A Clustal X multiple sequence alignment of region immediately downstream of the ORF1a/ORF1b RFS that has been predicted previously to adopt a stem-loop structure in NDiV. The alignment illustrates sequence variations in nucleotides predicted in NDiV to be involved in base pairs that sustain the structure.

```
BBaV (JKT_9853)      GGATTTTCCAATTGGGCGCGTTCTTTGAGCAGGAATAGTAATGTCGCCCGCTATCACCAACTATGTACTAATACCACACATGGTATGCGACACAC
BBaV (JKT_9876)      GGATTTTCCAATTGGGCGCGTTCTTTGAGCAGGAATAGTAATGTCGCCCGCTATCACCAACTATGTACTAATACCACACATGGTATGCGACACAC
BBaV (JKT_9891)      GGATTTTCCAATTGGGCGCGTTCTTTGAGCAGGAATAGTAATGTCGCCCGCTATCACCAACTATGTACTAATACCACACATGGTATGCGACACAC
BBaV (JKT_7774)      GGATTTTCCAATTGGGCGCGTTCTTTGAGCAGGAATAGTAATGTCGCCCGCTATCATCAACTATGTACTAATACCACACATGGTATGCGACACAC
KSaV (JKT_10701)     GGATTTTCCTATTGGGCGCGTTCTTTGAGCAGGAATAGTAATGTCGCCCGCTACCACCAACTACGCACAAATACCAAAACTGGTATTCGACATAC
HouV (V3872)         GGATTTTCAAGGTGGGCTCGTACTCTAAGCAGGAACAGTAATGTAGCCCGATATCACCAACTATGTACCACCACCAATGCTGGTATAAGACACAC
HouV (V3982)         GGATTTTCAAGGTGGGCTCGTACTCTAAGCAGGAACAGTAATGTAGCCCGATATCACCAACTATGTACCACCACCAATGCTGGTATAAGACACAC
HouV (16740)         GGATTTTCAAGGTGGGCTCGTACTCTAAGCAGGAACAGTAATGTAGCCCGATATCACCAACTATGTACCACCACCAATGCTGGTATAAGACACAC
HouV (16757)         GGATTTTCAAGGTGGGCTCGTACTCTAAGCAGGAACAGTAATGTAGCCCGATATCACCAACTATGTACCACCACCAATGCTGGTATAAGACACAC
NgeV (JKT_9982)      GGATTTTCAAGGTGGGCTCGTACTCTAAGCAGGAACAGTAATGTAGCCCGATATCACCAACTATGTACCACCACTAATGCTGGTATAAGACACAC
NDiV                 GGATTTTCAAGGTGGGCTCGTACTCTAAGCAGGAACAGTAATGTAGCCCGATATCACCAACTATGTACCACCACTGATGCTGGTATAAGACACAC
HanaV                GGATTTTCTAAGTGGGCTCGTACTTTAAGCAGGAACAGTAATGTAGCCCGATATCATCAGCTTTGCACCAACACTAATGTAGGTACAAGATATAC
CavV                 GGATTTTCAAAGTGGGCTCGTACTCTAAGCAGGAACAGTAATGTAGCCCGATATCACCAATTATGTACCAATACGAATAATGGTATACGCCACAC
KPhV (KP84_0156)     GGATTTTCCACTTGGGCGCGTTCTTTAAGCAGGAATAGTAATGTTGCCCGTTATCACCAACTATGTACCAATACTAAGCATGGTACACGCCACAC
KPhV (KP84_0192)     GGATTTTCCACTTGGGCGCGTTCTTTAAGCAGGAATAGTAATGTTGCCCGTTATCACCAACTATGTACCAATACTAAGCATGGTACACGCCACAC
KPhV (KP84_0344)     GGATTTTCCACTTGGGCGCGTTCTTTAAGCAGGAATAGTAATGTTGCCCGTTATCACCAACTATGTACCAATACTAAGCATGGTACACGCCACAC
MenoV                GGATTTTCAAAATGGGCACGTTCATTAAGCAGGAATACCAACGTCGCCCGATATCATCAAATCCGTACTGTTAGCAAGCAAAATGTTCGCTATAC
NseV                 GGATTTTCAAAATGGGCTCGTACTTTAAGCAGGAATAGTAATGTAGCCCGTTACCATCAATTACACACTAAGAGATTAGGTGTTGAGCGCTATAC
                     ********    ***** *** *  * ******** *  ** ** ***** ** ** **  *    **    *          *    *  * **
```
